# The Satellite Cell Niche Regulates the Balance between Myoblast Differentiation and Self-Renewal via p53

**DOI:** 10.1016/j.stemcr.2018.01.007

**Published:** 2018-02-08

**Authors:** Valentina Flamini, Rachel S. Ghadiali, Philipp Antczak, Amy Rothwell, Jeremy E. Turnbull, Addolorata Pisconti

**Affiliations:** 1 Department of Biochemistry, Institute of Integrative Biology, University of Liverpool, Liverpool L69 7ZB, UK; 2 Department of Functional Genomics, Institute of Integrative Biology, University of Liverpool, Liverpool L69 7ZB, UK; 3 Computational Biology Facility, Institute of Integrative Biology, University of Liverpool, Liverpool L69 7ZB, UK

**Keywords:** satellite cells, muscle stem cells, niche, p53, satellite cell quiescence, self-renewal, asymmetric satellite cell division, gene expression regulation, differentiation, transcriptomics

## Abstract

Satellite cells are adult muscle stem cells residing in a specialized niche that regulates their homeostasis. How niche-generated signals integrate to regulate gene expression in satellite cell-derived myoblasts is poorly understood. We undertook an unbiased approach to study the effect of the satellite cell niche on satellite cell-derived myoblast transcriptional regulation and identified the tumor suppressor p53 as a key player in the regulation of myoblast quiescence. After activation and proliferation, a subpopulation of myoblasts cultured in the presence of the niche upregulates p53 and fails to differentiate. When satellite cell self-renewal is modeled *ex vivo* in a reserve cell assay, myoblasts treated with Nutlin-3, which increases p53 levels in the cell, fail to differentiate and instead become quiescent. Since both these Nutlin-3 effects are rescued by small interfering RNA-mediated p53 knockdown, we conclude that a tight control of p53 levels in myoblasts regulates the balance between differentiation and return to quiescence.

## Highlights

• The satellite cell niche drives a transcriptional program that promotes self-renewal
• An asymmetric increase in p53 inhibits differentiation in niche-associated myoblasts
• A sustained increase in p53 promotes quiescence at the expense of differentiation

## Graphical Abstract

**Figure:**
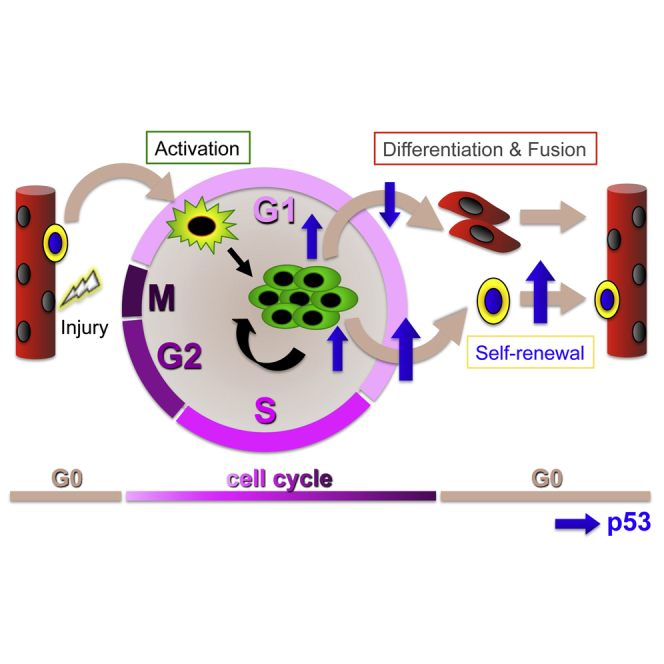

## Teaser

In this article, Pisconti et al. use an unbiased approach to show that the transcriptional program that drives myoblast cell fate transitions is directly regulated by the nature of the surrounding niche. The authors identify p53 as a niche-induced transcriptional regulator that increases in a subset of myoblasts upon cell-cycle exit, promoting quiescence at the expense of differentiation.

## Introduction

Satellite cells (SCs) are quiescent muscle stem cells residing in a specialized anatomical niche located between the plasma membrane of the muscle fiber and the surrounding basal lamina (Mashinchian et al., 2017). In response to muscle damage, SCs become activated and re-enter the cell cycle. After one or more rounds of proliferation, the vast majority of SC-derived muscle progenitors (called myoblasts) exit the cell cycle and enter a terminal G0 phase that leads to differentiation, followed by fusion to existing damaged muscle fibers to repair them or one-another to generate new muscle fibers. During this process, a small portion of myoblasts do not differentiate and rather enters a reversible G0 phase of the cell cycle, effectively replenishing the pool of quiescent SCs (Olguín and Pisconti, 2012). The mechanisms that regulate these fate decisions have been investigated, and at least two models have been proposed. In the first model, activated SCs divide asymmetrically upon activation giving rise to a daughter cell that self-renews and another daughter cell that becomes a myoblast and gives rise to a myogenic progeny (Dumont et al., 2015, Kuang et al., 2007, Troy et al., 2012). In a second model, proliferating myoblasts are induced to overexpress *Pax7*, which in turn inhibits myogenin expression and promotes entry into a mitotically quiescent state (Olguín and Olwin, 2004, Wen et al., 2012). In both cases, a key role appears to be played by the extracellular environment, called the SC niche (Mashinchian et al., 2017).

The SC niche is the complex set of molecules surrounding the SC in its anatomical location and the receptors that are expressed on its surface. Several of these molecules play important roles in driving SC fate (Mashinchian et al., 2017). However, it is also well established that when SCs are completely stripped of their native niche and cultured on a proteinaceous substrate, usually collagen, laminin, or gelatin, they retain the capacity to recapitulate the fate choices normally made in the presence of the niche, including proliferation, differentiation, fusion, and generation of a population of quiescent cells resembling self-renewed SCs (Olguín and Olwin, 2004). These observations raise two questions: (1) Are SCs primed to follow the myogenic program regardless of the presence of the niche? (2) Is the transcriptional program that drives these fate decisions in SCs the same in the presence of the niche and its absence?

Here we attempt to answer these questions by investigating gene expression in SC-derived myoblasts cultured under two different conditions: in the presence of their native niche (on isolated myofibers) or in its absence (on gelatin-coated dishes). We show how myoblast gene expression is affected by the presence of the niche and identify the p53 gene network as a key regulator of myoblast fate in the presence of the SC niche. Lastly, we show that a sustained increase in p53 during myoblast cell-cycle exit inhibits myoblast differentiation while promoting quiescence.

## Results

### Dispersed and Myofiber-Associated Myoblasts Exit the Cell Cycle and Initiate Differentiation with Similar Timing

**Figure 1:**
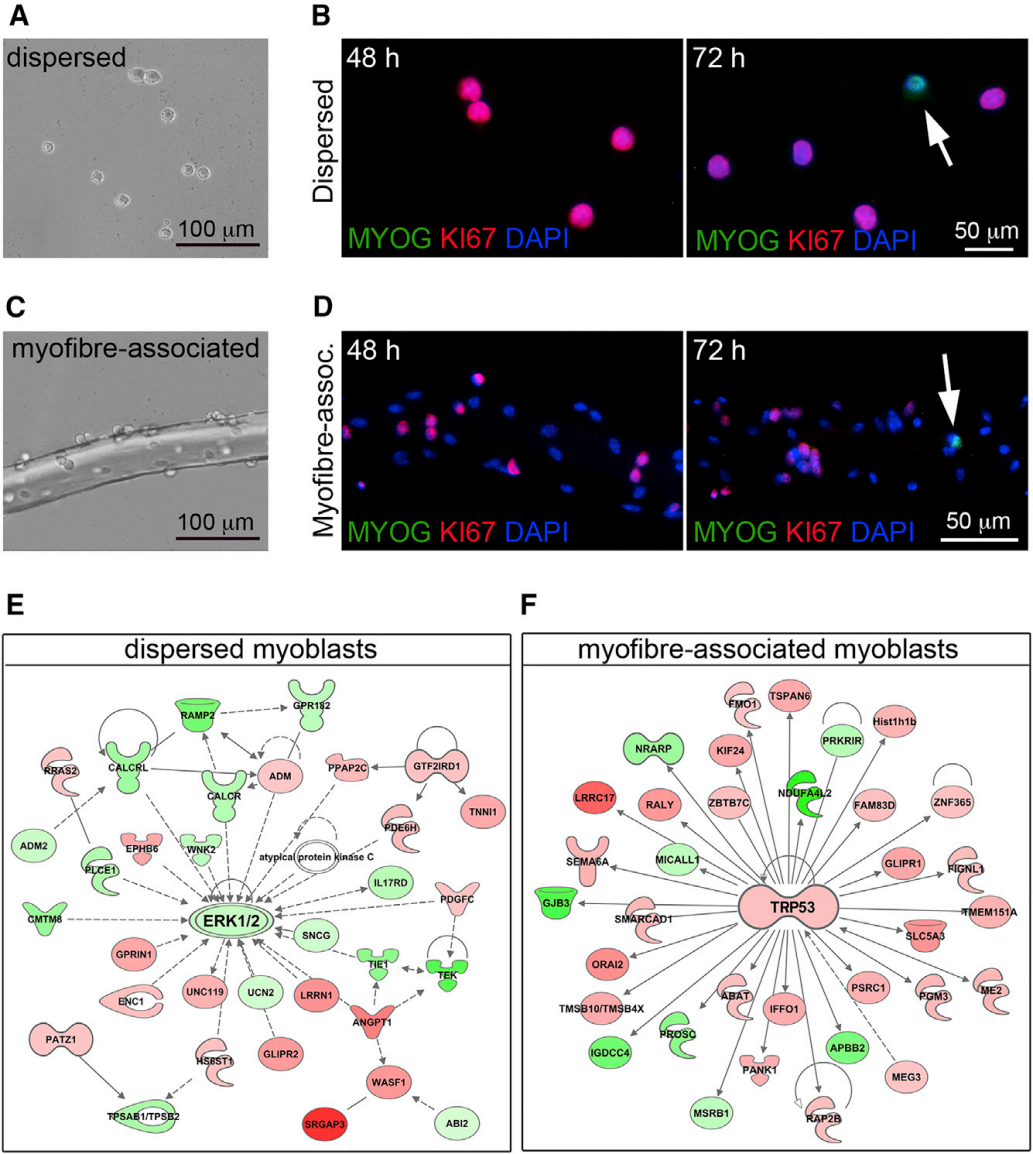
Cell-Cycle Exit and Terminal Differentiation Are Induced in Both Myofiber-Associated and Dispersed Myoblasts between 48 and 72 hr after Isolation (A and B) Dispersed myoblasts cultured on gelatin-coated plates show a rounded morphology (A) and proliferate extensively in the first 2–3 days as revealed by positive staining for the cell-cycle marker KI67+. No differentiating cells are detected at 48 hr after isolation (B). As early as 72 hr post-isolation occasionally MYOG+ cells are detected in dispersed cultures (B), arrow. (C and D) For the first 2 days myofiber-associated myoblasts (C) proliferate as revealed by positive staining for KI67+ and absence of differentiating (MYOG+) cells (D). At 72 hr after isolation a few MYOG+ cells are occasionally detected (D), arrow. (E and F) Genes differentially expressed between 48 and 72 hr in dispersed (E) and myofiber-associated (F) myoblasts were mapped to canonical gene networks using IPA, revealing that the top most enriched gene network in dispersed myoblasts is centered around *Erk1/2* downregulation (E), while the top most enriched network in myofiber-associated myoblasts is centered around *Trp53* upregulation (F). Genes labeled in green are downregulated, genes labeled in red are upregulated at 72 hr compared to 48 hr. The color intensity is proportional to the extent of up- or downregulation.

When SCs are isolated from the muscle tissue and cultured on gelatin-coated dishes, they extensively proliferate for the first 2–3 days in culture (Figures 1A, 1B, and S1). Proliferating SCs express *Pax7*, *Myf5*, and *MyoD1* and are often referred to as myoblasts. On the fourth day in culture, a few myotubes can be already observed (Figure S1). Indeed, myogenin-positive (MYOG+) cells are occasionally observed on the third day in culture (Figure 1B), suggesting that SC-derived myoblasts in dispersed cultures begin to exit the cell cycle and undergo terminal differentiation between 48 and 72 hr after isolation. Similarly, on day 3 in culture, MYOG+ cells are observed amongst myofiber-associated myoblasts (Figures 1C and 1D), which are cultured in the same medium as dispersed myoblasts. This suggests that the timing of myoblast cell-cycle exit and entry into terminal differentiation are comparable regardless of the presence of the niche. To test whether these comparable timings were driven by comparable transcriptional programs, we carried out a global gene expression analysis of SC-derived myoblasts cultured either in dispersed cultures or on explanted myofibers. We profiled gene expression in myoblasts from both cell culture types at 48 and 72 hr after isolation, when cell-cycle exit and commitment to terminal differentiation appear to occur under both culture conditions (Figures 1A–1D).

### Myoblast Cell-Cycle Exit Is Associated with Different Transcriptional Signatures in the Presence or Absence of the SC Niche

We collected four biological replicates for each time point (48 and 72 hr) in each culture condition and analyzed gene expression by microarray technology. The extent of reproducibility across replicates was excellent (Figures S2A and S2B). By contrast, the myoblast transcriptome at 48 hr was remarkably different from the transcriptome at 72 hr under both culture conditions, as evidenced by the large number of differentially expressed genes (at q < 0.01) detected between 48 and 72 hr under either culture conditions: 1,810 in dispersed myoblasts and 1,999 in myofiber-associated myoblasts. Interestingly, when we compared the 72 hr/48 hr fold changes between the two culture conditions, it appeared evident that gene expression changes between 48 and 72 hr were different in the two culture conditions (Figure S2C). To gain insight into the molecular mechanisms that were associated with these dramatic changes in the transcriptional signature of myoblasts between 48 and 72 hr in either dispersed or myofiber-associated cultures, we mapped the differentially expressed genes to known gene networks using Ingenuity Pathway Analysis (IPA). The top most enriched network to which differentially expressed genes from dispersed myoblasts mapped, was centered around a decrease in the intracellular kinases *Erk1* and *Erk2* (Figure 1E). In contrast, the top most enriched network to which differentially expressed genes from myofiber-associated myoblasts mapped, was centered around an increase in the tumor suppressor *Trp53* (p53) (Figure 1F). ERK1/2 are key promoters of myoblast proliferation (Jones et al., 2001) and, similarly, an increase in p53 levels is expected to lead to cell-cycle arrest (Levine, 1997). Thus, these results are consistent with our initial hypothesis that between 48 and 72 hr both dispersed and myofiber-associated myoblasts prepare to exit the cell cycle, though via different molecular mechanisms.

### The Signaling Pathways that Regulate Cell-Cycle Exit in the Presence or Absence of the Niche Are Different

**Figure 2:**
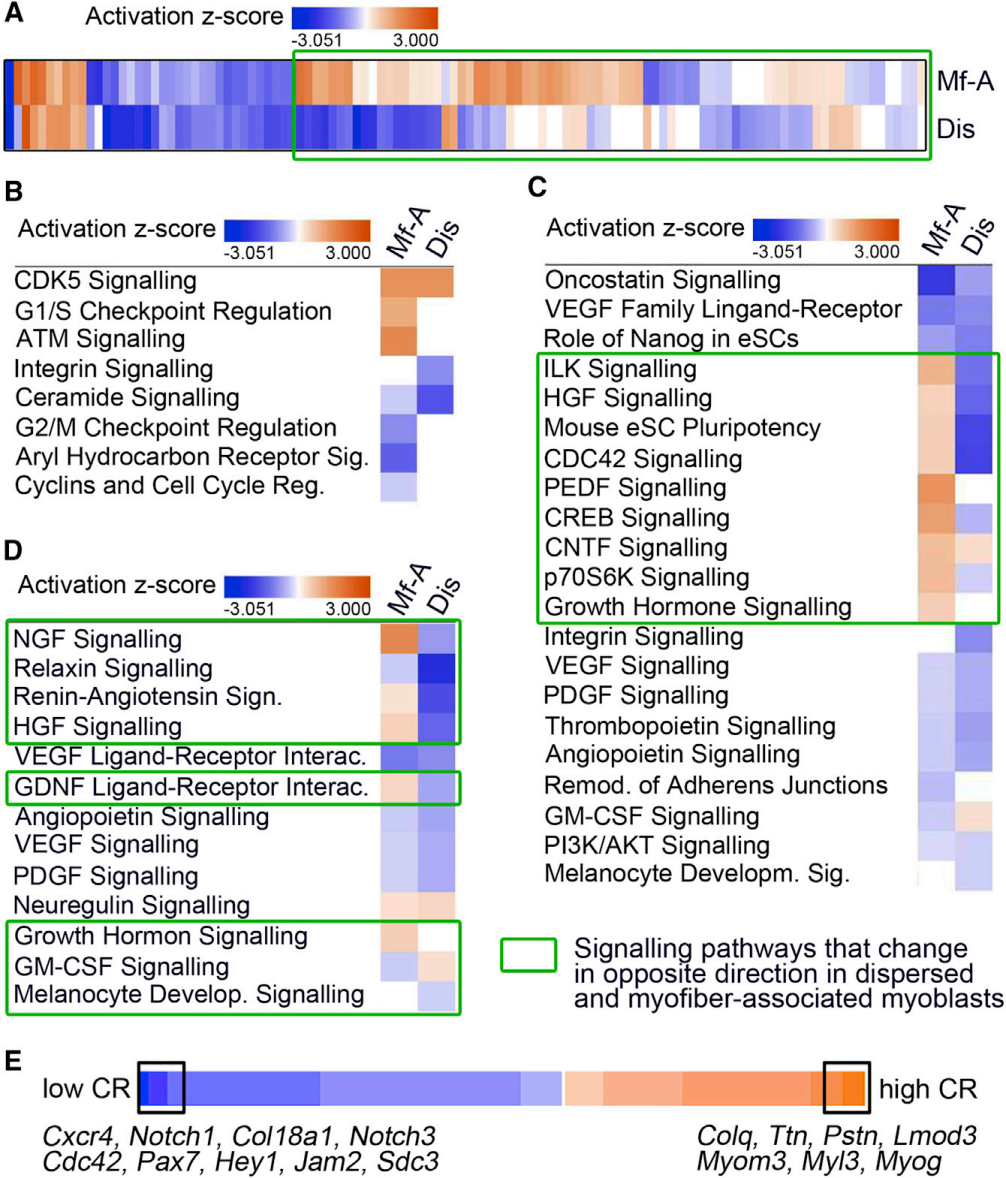
Canonical Signaling Pathways Are Differentially Activated in Dispersed and Myofiber-Associated Myoblasts (A) Genes differentially expressed between 48 and 72 hr post-isolation in dispersed (Dis) and myofiber-associated (Mf-A) myoblasts were functionally mapped to all canonical signaling pathways listed by IPA. For each signaling pathway, an enrichment p value and a *Z* score of activation were calculated and the pathways with enrichment p value < 0.01 (–log[p value] > 1.3) are plotted as heatmaps, were orange represents a positive *Z* score (= activation), blue is a negative *Z* score (deactivation), and white is *Z* = 0. Canonical signaling pathways that were not enriched enough to show a *Z* score were excluded. Canonical signaling pathways that change in opposite direction in dispersed and myofiber-associated myoblasts are highlighted by a green box. (B–D) Heatmaps obtained as in (A) for canonical signaling pathways mapping to the IPA categories: Cell Cycle (B), Cell Growth and Proliferation (C), and Growth Factor Signaling (D). (E) Heatmap distribution of the comparative ratio (CR) = ([72 hr/48 hr]_dispersed_/[72 hr/48 hr]_myofiber-associated_) for gene families that are involved in myogenesis (manual annotation, see Experimental Procedures section).

To further our understanding of the molecular mechanisms regulating SC gene expression in the presence and absence of the SC niche, we analyzed the canonical signaling pathways that were enriched between 48 and 72 hr in dispersed and myofiber-associated myoblasts using IPA, which assigns an activation score to a signaling pathway based on the direction and extent of change of the differentially expressed genes mapped to that signaling pathway. The first observation was that the vast majority of canonical signaling pathways moved in different directions between 48 and 72 hr in myofiber-associated versus dispersed myoblasts (Figure 2A).

When we then analyzed in more detail the IPA category *Cell Cycle*, the most striking feature was a strong activation of *Cdk5 signaling* (Figure 2B) observed in both culture conditions, indicating cell-cycle arrest and preparation to differentiate (Lazaro et al., 1997, Sarker and Lee, 2004). In myofiber-associated myoblasts this was accompanied by an even stronger activation of *ATM signaling*, which is upstream of p53 and also induces cell-cycle arrest (Figure 2B). Consistently, signaling pathways that promote proliferation, such as *Ceramide signaling* (Gangoiti et al., 2012), *G2/M transition signaling*, *Cyclin signaling*, and *Aryl hydrocarbon receptor signaling* (Barouki et al., 2007, Yin et al., 2016), were inactivated in myofiber-associated myoblasts (Figure 2B). Similarly, dispersed myoblasts showed a marked inactivation of *Integrin signaling*, also pointing toward inhibition of proliferation (Figure 2B). Thus, canonical signaling pathways related to cell-cycle regulation are activated or deactivated in both culture conditions in a manner that supports preparation to exit the cell cycle. The only exception to this was the *G1/S checkpoint regulation* signaling pathway, which was activated in myofiber-associated but not dispersed myoblasts (Figure 2B). Similarly, the IPA category *Cell Growth and Proliferation* showed a series of pro-proliferative signaling pathways (ILK, HGF, PDGF, CREB, P70S6K, and CDC42 signaling pathways) that were activated in myofiber-associated myoblasts but deactivated in dispersed myoblasts (Figure 2C). Since proliferation and cell-cycle exit are mutually exclusive, the fact that both pro-proliferation and pro-cell-cycle exit pathways are activated in myofiber-associated myoblasts, supports previous findings that myofiber-associated myoblasts are heterogeneous (Ono et al., 2010).

Lastly, we analyzed the IPA category *Growth Factor Signaling* (Figure 2D). Amongst the signaling pathways that were differentially activated between dispersed and myofiber-associated myoblasts we found activation of pro-proliferation signaling (*HGF*) and self-renewal signaling (GDNF signaling, which requires syndecan-3 (SDC3), and therefore is expected to promote self-renewal, Bespalov et al., 2011, Pisconti et al., 2010) in myofiber-associated but not in dispersed myoblasts.

### Comparative Analysis of Gene Expression Suggests a Role for the Niche in Driving Self-Renewal

To understand the differences in myoblast gene expression changes due to culture conditions we compared ratios of 72 hr/48 hr mRNA levels within each culture condition with each other (Figure S2C) and generated a fold-change ratio using the formula:

$$\text{Comparative}\;\text{ratio}\left(\text{CR}\right)=\frac{\left(\text{mRNA}\;\text{at}\;72\;\text{hr/mRNA}\;\text{at}\;48\;\text{hr}\right)\text{dispersed}}{\left(\text{mRNA}\;\text{at}\;72\;\text{hr/mRNA}\;\text{at}\;48\;\text{hr}\right)\text{myofiber-associated}}$$

Genes that score high CR values (>1) are genes that increase in dispersed but not in myofiber-associated myoblasts between 48 and 72 hr, while genes that score low CR values (<1) are genes that increase in myofiber-associated but not in dispersed myoblasts between 48 and 72 hr. We identified 2,583 genes with CR > 1 and 3,120 genes with CR < 1 at 1% false discovery rate (FDR), suggesting very different time-dependent responses between the two culture conditions. After filtering the resulting list of genes to include only genes with a q value < 0.01, we then functionally analyzed these genes by mapping them to the gene ontology (GO) category *Biological Process* using the online tool DAVID (Tables S1 and S2). Table S1 shows the GO annotation of genes that had CR > 2, while Table S2 shows the GO annotation of genes that had CR < 0.5.

The most significant GO terms to which genes that increase in dispersed but not in myofiber-associated myoblasts (CR > 2) map are associated with cell movement (*Taxis*) and muscle differentiation (*Striated Muscle Differentiation*) (Table S1). Interestingly, the term *Negative regulation of transport* also included genes that promote muscle differentiation, such as *Il-6* and *Nos1* (De Palma et al., 2010, Hoene et al., 2013). In contrast, the genes that increase between 48 and 72 hr in myofiber-associated myoblasts but not in dispersed myoblasts (CR < 0.05) map to *Cell Adhesion, Differentiation*, and *Cell Fate Commitment* (Table S2). The latter appears to be related mostly to maintenance of stemness as it contains genes that are associated with stemness in SCs (*Notch1, Notch3, Pax7,* and *Sox8*; Bjornson et al., 2012, Gopinath et al., 2014, Olguín and Olwin, 2004, Olguín and Pisconti, 2012, Pisconti et al., 2010, Schmidt et al., 2003).

Lastly, we organized the differentially expressed genes according to their CR and filtered the gene list through a manually curated list of gene families that have been shown to play a role in myogenesis (see Experimental Procedures for details). This analysis showed that genes associated with muscle differentiation were increased in dispersed but not in myofiber-associated myoblasts (Table S3; Figure 2E: *Myog, Myl3, Myom3, Lmod3, Postn, Ttn,* and *Colq*). By contrast, genes associated with self-renewal were increased in myofiber-associated but not dispersed myoblasts (Table S3; Figure 2E: *Cxcr4, Col18a1, Notch1, Notch3, Cdc42, Pax7, Hey1, Jam2,* and *Sdc3*).

To summarize the data shown so far: *Erk1/2* downregulation in dispersed myoblasts is mostly associated with activation of signaling pathways that lead to cell-cycle arrest and the onset of differentiation. In contrast, *Trp53* upregulation in myofiber-associated myoblasts is associated with a more heterogeneous transcriptomic signature in which signaling leading to cell-cycle arrest, differentiation, self-renewal, and proliferation co-exist. Since the role of p53 in myogenesis is complex and not well understood, we decided to focus on understanding how an increase in p53 levels might affect myoblast cell fate.

### An Asymmetric Increase in p53 Protein Levels Is Incompatible with Myogenic Differentiation in Myofiber-Associated Myoblasts

The expression of p53 is rapidly and transiently upregulated in the first hours of C2C12 myoblast differentiation induced by serum deprivation (Halevy, 1993), and is important for myogenic differentiation, as expression of a p53 dominant-negative mutant leads to impaired differentiation (Soddu et al., 1996), but is not involved in differentiation-induced apoptosis (Cerone et al., 2000). Thus, it could be speculated that the upregulation of p53 that we observe in myofiber-associated myoblasts drives differentiation. However, it has been recently shown that a sustained increase in p53 levels, such as that due to genotoxic stress, or treatment with the MDM2 inhibitor Nutlin-3, impairs myogenic differentiation, possibly via direct inhibition of myogenin expression (Walsh et al., 2015, Yang et al., 2015). Moreover, p53 mediates hypoxia-induced inhibition of myoblast differentiation (Wang et al., 2015).

**Figure 3:**
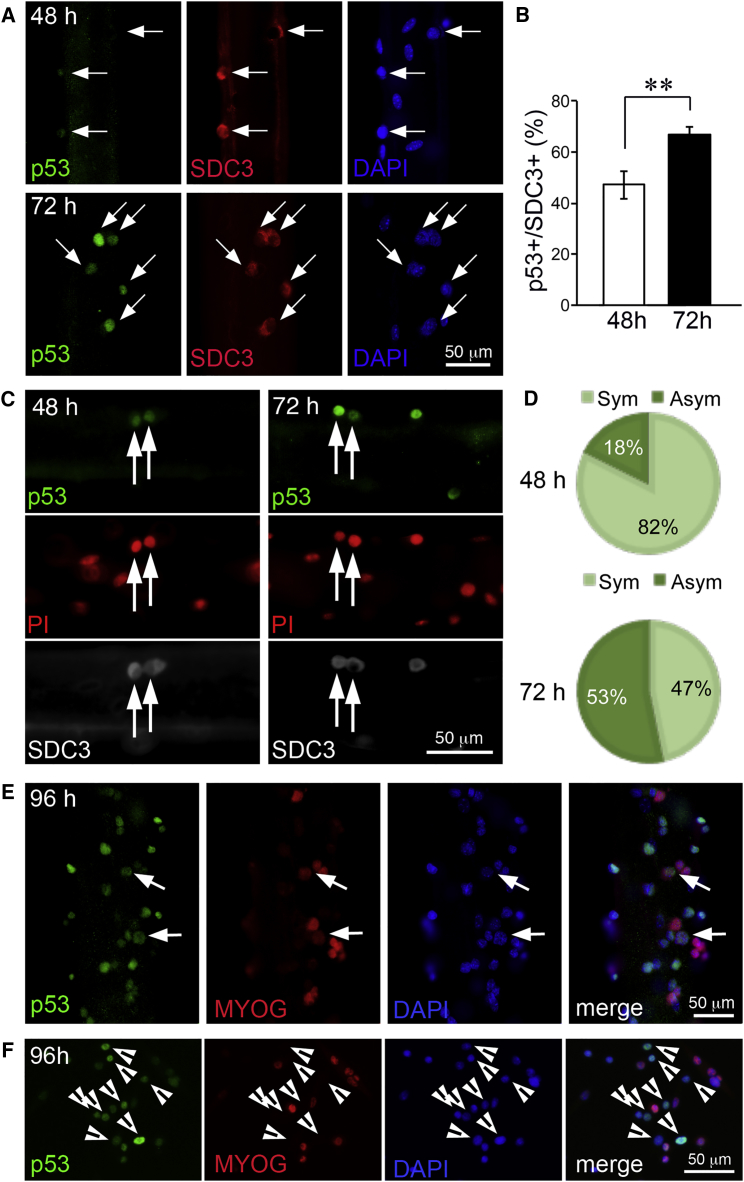
p53 Increases over Time in a Subset of Myofiber-Associated Myoblasts and Is Asymmetrically Distributed (A) Individual myofibers were isolated and cultured in suspension for 48 and 72 hr prior to fixation and immunostaining to detect p53 (green), the myoblast marker syndecan-3 (SDC3, red), and DNA (DAPI, blue). Arrows indicate SDC3+ SCs. (B) Quantification of (A) where at least 15 myofibers across 3 independent experiments had been scored, and the percentage of p53+ cells over the total number of SDC3+ cells plotted as average ±SEM. ^∗∗^p < 0.01. (C) Individual myofibers were isolated and cultured as in (A) then immunostained to detect p53 (green), SDC3 (white), and DNA (PI, red). Arrows indicate doublets of dividing cells where p53 is distributed either symmetrically (48 hr, left panels) or asymmetrically (72 hr, right panels). (D) Quantification of (C) where at least 15 fibers across 3 independent experiments had been scored. (E and F) p53+/MYOG– myoblasts are very abundant in myofiber cultures (E) but less in dispersed cultures (F). Individual myofibers or primary myoblasts were isolated and cultured for 96 hr, then fixed and immunostained to detect p53 (green), myogenin (MYOG, red), and DNA (DAPI, blue). Arrows in (E) indicate p53+/MYOG+ cells, while all the other cells are either p53+/MYOG– or p53-/MYOG+. Arrowheads in (F) indicate p53+/MYOG– cells. All the other cells are p53+/MYOG+.

To investigate the role of the increase in p53 gene expression detected by microarray in myofiber-associated myoblasts, we first tested whether such an increase in mRNA levels was accompanied by an increase in p53 protein levels and whether it was associated with differentiation. Indeed, p53 levels appeared to increase in myofiber-associated myoblasts, identified by the general myoblast marker SDC3 (Cornelison et al., 2001) between 48 and 72 hr in culture, as measured by an increase in the proportion of p53+/SDC3+ myoblasts (Figures 3A and 3B). Intriguingly, we occasionally observed an asymmetric distribution of p53 in myoblast doublets (Figure 3C), which was more prominent at 72 hr (Figure 3D), and was accompanied by an almost perfect inverse correlation with myogenin (MYOG) expression at 96 hr (Figure 3E). When we measured p53 protein levels across the same 3-day time course in dispersed myoblasts, we found only a small and not significant increase in p53 protein levels over time (Figure S3). Moreover, immunofluorescence analysis of primary dispersed myoblasts showed that p53 protein was still present in MYOG+ dispersed myoblasts, although often at lower levels than in MYOG– cells (Figure 3F). These results suggest that, when myoblasts are cultured in the presence of their native niche, p53 protein levels increase over time dramatically and selectively in some cells, and in these cells myogenic differentiation is inhibited (Figure 3E). When the niche is absent, such a dramatic and sustained increase occurs in fewer cells, while the majority of cells maintain lower levels of p53, which are permissive of myogenic differentiation (Figure 3F).

**Figure 4:**
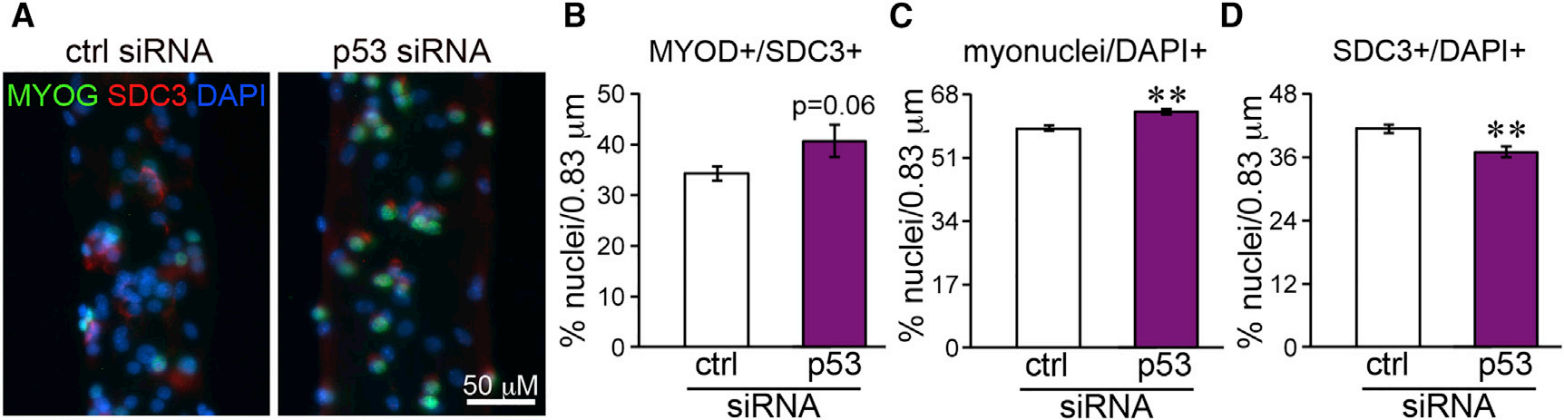
p53 Knockdown Promotes Myoblast Differentiation in Myofiber Cultures (A) Individual myofibers were isolated and cultured in suspension for 48 hr prior to transfection with either a specific p53 siRNA or a scrambled (ctrl) siRNA and 4 hr later fixed and immunostained to detect myogenin (MYOG, green), syndecan-3 (SDC3, red), and DNA (DAPI, blue). (B) Differentiated myoblasts were scored as percentage of MyoG+/SDC3+ myoblasts over total nuclei per unit length across at least eight myofibers/experiment in three independent experiments (N > 24), and plotted as average ± SEM. (C) The number of myonuclei per unit length was measured as total number of DAPI+ nuclei minus the number of nuclei contained in SDC3+ cells (since SDC3 marks SCs and myoblasts at all stages in myogenesis) per unit length across at least eight myofibers/experiment in three independent experiments (N > 24), and plotted as average ± SEM. (D) The percentage of myoblasts was calculated as percentage of nuclei contained in SDC3+ cells over the total number of DAPI+ nuclei per unit length across at least eight myofibers/experiment in three independent experiments (N > 24), and plotted as average ± SEM. ^∗∗^p < 0.01 when comparing the indicated population scored in control siRNA-transfected cultures with the same population scored in p53 siRNA-transfected cultures.

If it is true that the presence of the SC niche promotes p53 upregulation in a subpopulation of myofiber-associated myoblasts and that this in turn inhibits myogenic differentiation in these cells, then decreasing p53 in myofiber-associated myoblasts via RNAi should promote myoblast differentiation in myofiber cultures. To test this hypothesis, we transfected myofiber cultures with either p53 small interfering RNA (siRNA) or with control (scrambled) siRNA at 48 hr post-isolation. Transfection of C2C12 myoblasts was used to validate p53 knockdown efficiency (Figure S4). Two days after transfection, we fixed and immunostained the myofibers to detect and quantify MYOG+ myoblasts (Figure 4A). As predicted, the percentage of MYOG+/SDC3+ cells was increased in p53 siRNA-transfected cultures compared with control siRNA-transfected cultures, although the difference did not reach statistical significance (Figure 4B). The fact that the difference was small, could be due to the high propensity of differentiated myofiber-associated myoblasts to immediately fuse with the underlying fiber (Kuang et al., 2007), which removes them from the equation when MYOG+/SDC3+ cells are scored as a measure of differentiation. Indeed, the number of myonuclei per unit length was significantly increased (Figure 4C), while the frequency of myoblasts (SDC3+ cells) per unit length was decreased (Figure 4D) upon p53 knockdown, supporting the idea that p53 inhibits differentiation in a subpopulation of myofiber-associated myoblasts.

The next question was: what is the fate of these myoblasts that show high p53 protein levels and fail to differentiate? Since high levels of p53 are incompatible with proliferation, we hypothesized that a sustained increase in p53 levels in myoblasts would lead to apoptosis, senescence, or quiescence.

### An Increase in p53 Protein Levels Promotes Myoblast Quiescence

Nutlin-3, a small molecule compound that blocks the interaction between p53 and the E3 ligase MDM2, is often used to increase the levels of p53 protein in the cell (Vassilev, 2005). Nutlin-3 treatment of C2C12 myoblasts leads to a generalized increase in p53 protein levels and both proliferation and differentiation inhibition (Walsh et al., 2015), thus supporting our hypothesis that a sustained increase in p53 levels in primary myoblasts might lead to apoptosis, senescence, or quiescence, as well as differentiation inhibition. To test this hypothesis we treated primary dispersed myoblasts with increasing concentrations of Nutlin-3 and verified that indeed Nutlin-3 inhibits proliferation (Figure S5A). However, Nutlin-3 treatment does not seem to increase apoptosis in primary myoblasts (Figure S5B), which is consistent with the absence of a role for p53 in serum deprivation-induced myoblast apoptosis (Cerone et al., 2000). Since Nutlin-3 does not promote differentiation, in fact it inhibits differentiation (Figure S6), the lack of apoptosis suggests that Nutlin-3-mediated inhibition of proliferation is due to induction of either quiescence or senescence.

**Figure 5:**
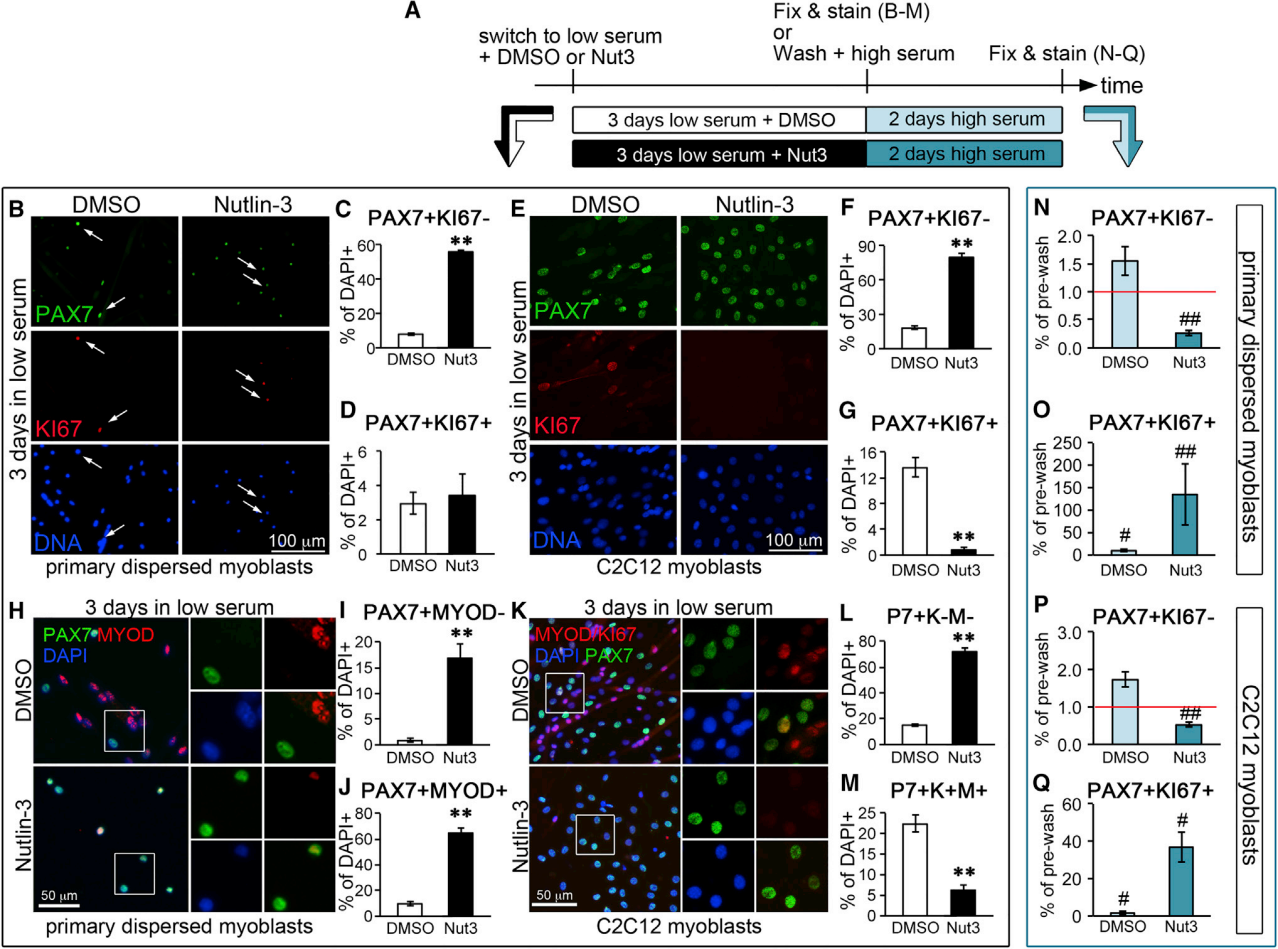
Nutlin-3 Promotes Reserve Cell Generation in Serum-Deprived Myoblasts (A) Schematic representation of the reserve cell assay experimental design. Primary and C2C12 myoblast cultures were switched to low serum medium to induce cell-cycle exit in the presence of either 20 μM Nutlin-3 (Nut3) or vehicle (DMSO). To distinguish between quiescence and senescence, myoblast cultures that had been maintained in low serum supplemented with either DMSO or Nutlin-3 for 3 days were re-exposed to high serum for 2 days. The experiment was repeated 3 times independently and each time 10–15 technical replicates were scored. (B–D) Primary myoblast cultures were treated as in (A) and, after 3 days in low serum, fixed, immunostained to detect PAX7 (green), KI67 (red), and DNA (DAPI, blue), and scored for the percentages of PAX7+/KI67– (C) and PAX7+/KI67+ (D) cells. In (B), one representative image for each treatment is shown. In (C) and (D) quantitative analyses of the indicated cell subpopulations across all three independent experiments were plotted as average ± SEM. The arrows in (B) indicate PAX7+/KI67+ cells. (E–G) C2C12 myoblasts cultures were treated as in (A) and, after 3 days in low serum, fixed, and immunostained as in (B). In (E) one representative image for each treatment is shown. In (F) and (G) quantitative analyses of the indicated cell subpopulations across all three independent experiments were plotted as average ± SEM. (H–M) Primary (H and J) and C2C12 (K–M) myoblasts that had been maintained in low serum supplemented with either DMSO or Nutlin-3 for 3 days were fixed and immunostained to detect PAX7 (green), KI67, and/or MYOD (red) and DNA (DAPI, blue). In (H) and (K) one representative image for each treatment is shown. Insets are enlarged on the side of the main image to show mutual exclusion or co-localization of PAX7 and MYOD (H) or PAX7 and MYOD+ KI67 (K). In (I), (J), (L), and (M) quantitative analyses of the indicated cell subpopulations across all three independent experiments were plotted as average ± SEM. (N–Q) In order to quantify true reserve cells, after 3 days in low serum, primary (N and O) and C2C12 (P and Q) myoblast cultures were re-exposed to high serum as depicted in (A) for 2 days, then fixed and immunostained to detect PAX7, KI67, and DNA. The percentages of PAX7+/KI67– (N and P) and PAX7+/KI67+ (O and Q) were calculated and plotted as fold change of the same population in cultures that had been maintained in low serum and then re-exposed to high serum (we will call it “post-wash” for simplicity) versus the same population in cultures maintained for only 3 days in low serum (indicated as “pre-wash”). The average of the post-wash/pre-wash fold change for each subpopulation across 3 independent biological replicates (each one scored for 10–15 technical replicates) was calculated and plotted ± SEM. In (C) and (D), (F) and (G), (I) and (J), and (L) and (M): ^∗∗^p < 0.01, where p is the p value of the average percentage of each cell subpopulation in Nut-3-treated cultures versus DMSO-treated cultures. In (N)–(Q), ^#^p < 0.05 and ^##^p < 0.01, where p is the p value of the fold change calculated as “subpopulation percentage in post-wash/subpopulation percentage in pre-wash” within each treatment (DMSO or Nut-3).

To discriminate between quiescence and senescence we performed a reserve cell assay. Cultures of both primary dispersed myoblasts and C2C12 myoblasts provide a validated *ex vivo* model of SC self-renewal: upon serum lowering, the vast majority of myoblasts exit the cell cycle with a portion of them downregulating PAX7 and undergoing terminal differentiation, and another portion maintaining high levels of PAX7 and becoming “reserve cells” (Yoshida et al., 1998). In reserve cell assays (Figure 5A), Nutlin-3 treatment of both primary and C2C12 myoblast cultures dramatically increased the percentage of putative reserve cells (PAX7+/KI67–) generated by serum lowering (Figures 5B, 5C, 5E, and 5F). This was accompanied by a decrease in the percentage of proliferating myoblasts (PAX7+/KI67+) in C2C12 cultures (Figures 5E and 5G), although no significant changes in PAX7+/KI67+ cells were detected in primary myoblast cultures (Figures 5B and 5D).

In addition to being mitotically quiescent, reserve cells also show decreased levels of MYOD (Yoshida et al., 1998), thus “true” reserve cells are PAX7+/KI67–/MYOD– (Yue et al., 2016). Although we could effectively label C2C12 myoblasts to simultaneously detect PAX7, KI67, and MYOD, this could not be achieved with primary myoblasts (triple PAX7/KI67/MYOD staining of primary myoblasts showed cytoplasmic positivity for MYOD and/or KI67, even at very low antibody concentration, which is not observed when either KI67 or MYOD antibodies are used in a double staining of C2C12 cells with PAX7). Thus, we were forced to label primary myoblasts separately for PAX7 and KI67 (as discussed above) and PAX7 and MYOD. Nutlin-3 treatment dramatically reduced MYOD protein levels and led to an increase in the frequency of true reserve cells (PAX7+/KI67–/MYOD–) in C2C12 myoblast cultures (Figures 5K–5L), which was accompanied by a decrease in proliferating myoblasts (PAX7+/MYOD+/KI67+; Figures 5K and 5M). Similarly, Nutlin-3 treatment produced a 20-fold increase in the numbers of uncommitted myoblasts (PAX7+/MYOD– cells; Figures 5H and 5I), but only a 6-fold increase in the percentage of committed myoblasts (PAX7+/MYOD+; Figures 5H and 5J) in primary myoblast cultures. Although in the absence of a triple PAX7/MYOD/KI67 staining it is not possible to establish whether Nutlin-3 promotes true reserve cell generation in primary myoblast cultures as it does in C2C12 cultures, the observations that: (1) the increase in uncommitted myoblasts produced by Nutlin-3 is over 3 times greater than the increase in committed myoblasts (20- versus 6-fold), and (2) PAX7+/KI67+ myoblasts do not increase in response to Nutlin-3 in primary cultures (Figure 5D), while PAX7+/KI67– cells increase 7-fold (Figure 5C), suggest that the percentage of true reserve cells (PAX7+/KI67–/MYOD–) is likely to significantly increase in primary myoblast cultures as it does in C2C12 cultures in response to Nutlin-3 treatment.

In contrast to differentiated and senescent cells, which are in an irreversible G0 phase, reserve cells are quiescent, in a reversible G0 phase, and therefore can be induced to re-enter the cell cycle by adding serum to the culture medium (Yoshida et al., 1998). To discriminate whether the putative reserve cells generated in myoblast cultures in response to Nutlin-3 treatment were truly quiescent or senescent, we exposed again to high serum the cultures that had been maintained for 3 days in low serum supplemented with either Nutlin-3 or DMSO, and 2 days later we measured the percentages of cells that had re-entered the cell cycle (Figure 5A). In both C2C12 and primary myoblast cultures washed and re-exposed to high serum, the percentage of PAX7+/KI67+ cells increased more dramatically if the cultures had been previously treated with Nutlin-3 compared with vehicle (Figures 5O and 5Q). Interestingly, the percentage of PAX7+/KI67– cells did not significantly change upon serum re-exposure in cultures that had been previously treated with vehicle, but significantly decreased in cell cultures that had been previously treated with Nutlin-3 (Figures 5N and 5P). These results indicate that the percentage of *true* reserve cells is normally very low in our reserve cell assay, regardless of the origin of the myoblasts used (primary or C2C12). However, this percentage of *true* reserve cells is dramatically increased by Nutlin-3 treatment.

### An Increase in p53 Levels Is Directly Responsible for the Increased Reserve Cell Generation in Response to Nutlin-3

**Figure 6:**
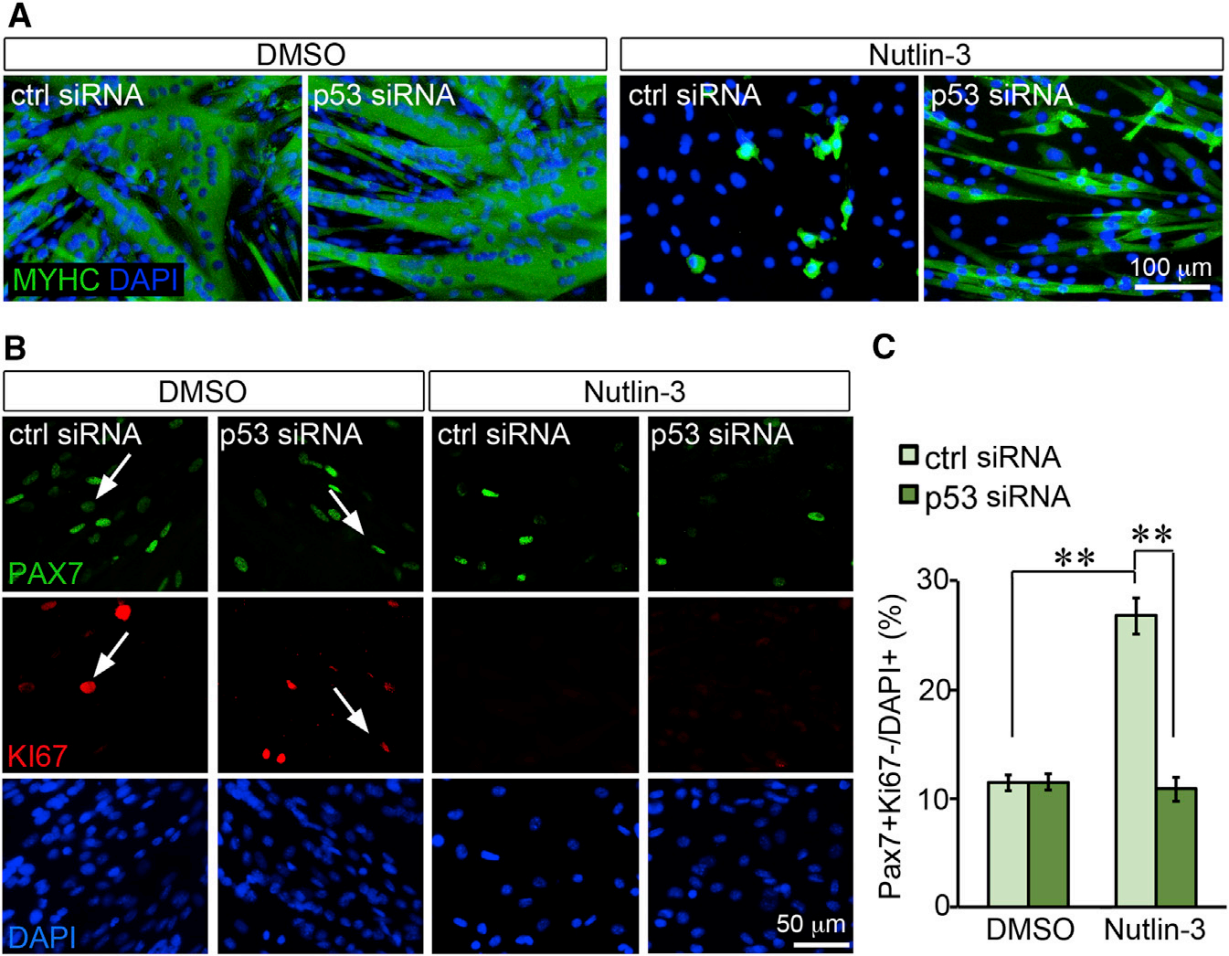
p53 Knockdown Rescues Reduced Differentiation and Increased Reserve Cell Generation in Myoblasts Treated with Nutlin-3 (A) Differentiation of C2C12 myoblasts is reduced by Nutlin-3 treatment and rescued by p53 knockdown. C2C12 myoblasts were transfected with either control or p53 siRNA, then maintained for 3 days in low serum supplemented with either DMSO or 20 μM Nutlin-3 prior to fixation and immunostaining to detect myosin heavy chain (MYHC, green) and DNA (DAPI, blue). (B) Reserve cell (PAX7+/KI67–) generation is increased by Nutlin-3 treatment and rescued by p53 knockdown. C2C12 myoblasts were transfected and cultured as in (A) prior to fixation and immunostaining to detect PAX7 (green), KI67 (red), and DNA (DAPI, blue). Arrows point to PAX7+/KI67+ cells. All PAX7+ cells not labeled by an arrow are KI67–. All KI67+ cells that are not labeled by an arrow are PAX7–. (C) Quantification of (B) where the average percentage of PAX7+/KI67– cells (over total DAPI+ cells) is calculated across 10–15 technical replicates for 3 independent experiments (N = 35) and plotted. Error bars are SEM. ^∗∗^p < 0.01.

To test whether Nutlin-3 promoted reserve cell generation and inhibited differentiation directly via p53, we transfected C2C12 myoblasts with either a control siRNA or with an anti-p53 siRNA, and 3 hr later we replaced the transfection medium with low serum medium (to induce cell-cycle exit followed by myotube and reserve cell generation) supplemented with either Nutlin-3 or vehicle. Although p53 siRNA transfection did not appear to alter the differentiation capacity of C2C12 myoblasts treated with vehicle, it partly rescued the differentiation levels of cells treated with Nutlin-3 (Figure 6A). More importantly, the percentage of PAX7+/KI67– reserve cells that was increased by Nutlin-3 in cultures transfected with control siRNA, was then rescued by transfection with a specific anti-p53 siRNA (Figures 6B and 6C), further indicating a direct role for p53 in regulating the balance between differentiation and reserve cell generation. Interestingly, the decrease in the percentage of PAX7+/KI67– reserve cells caused by p53 knockdown in Nutiln-3-treated cultures was not due to a rescue of the number of proliferating (PAX7+/KI67+) myoblasts (Figure 6B), but to a decrease in differentiation. This suggests that Nutlin-3 promotes reserve cell generation via p53 by affecting directly the cell fate decision to either differentiate or become quiescent, rather than the earlier decision to either divide or exit the cell cycle.

## Discussion

**Figure 7:**
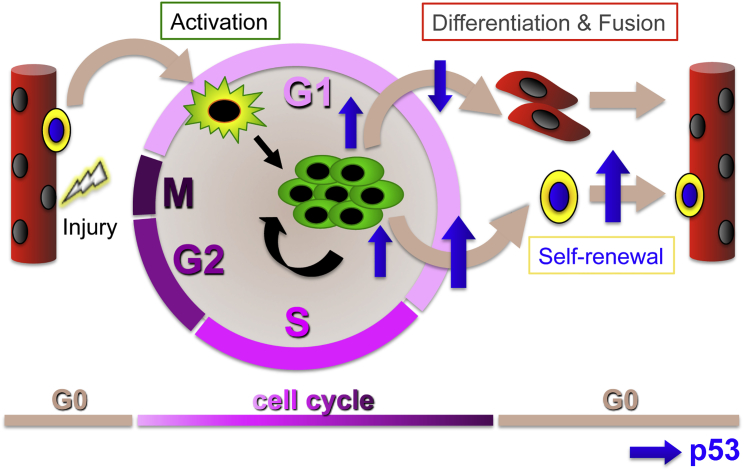
Schematic Representation of the Role of p53 in SC-Mediated Myogenesis In uninjured muscle (red myofiber on the left), SCs (oval yellow cell on the myofiber) are quiescent, in a reversible G0 phase of the cell cycle. Upon injury (bolt) SCs become activated and re-enter the cell cycle in G1 (yellow/green, star-shaped cell). At each division cycle the two daughter cells (myoblasts, green rounded cells) choose between three main fates: divide again (and complete the cell cycle), or exit the cell cycle and then either differentiate (red elongated cells), or become quiescent (again oval yellow cell). Although a transient increase in p53 levels (upward blue arrow) allows cell-cycle exit, p53 must return to basal levels in order for differentiation to proceed (downward blue arrow). In contrast, a further and sustained increase in p53 levels (larger upward blue arrow) leads to differentiation inhibition and promotion of self-renewal (return to a quiescent, undifferentiated state).

In this study, we show that primary myoblasts cultured *ex vivo* carry out different transcriptional programs to regulate their cell fate transitions according to whether they are cultured in the presence or absence of their native niche. In the absence of the niche, the vast majority of myoblasts activate a transcriptional program dominated by *Erk1/2* downregulation and differentiate. In the presence of the niche, the transcriptional program is dominated by an upregulation of *Trp53*. Validation experiments revealed that a sustained p53 increase in myofiber-associated myoblasts is restricted to a subpopulation of cells that fail to differentiate. These myoblasts showing high levels of p53 are more rare in the absence of the niche, which is likely why p53 upregulation is not significantly detected in the transcriptomic analysis of the dispersed myoblasts. Thus, our data suggest that the presence of the niche leads to a greater percentage of myoblasts showing a sustained increase in p53 levels and associated fail to differentiate. We then used gain- and loss-of-function experiments in a culture context that promotes cell-cycle exit to determine the fate of these high p53 myoblasts that fail to differentiate, and showed that for the most part these myoblasts become quiescent. Our data therefore point toward p53 signaling as a regulator of the balance between differentiation and self-renewal: although a transient increase in p53 levels occurs in the early hours that precede differentiation and is likely necessary for cell-cycle exit, p53 must quickly return to basal levels upon cell-cycle exit in order for differentiation to occur (Figure 7). However, in some cells, p53 levels further increase and remain high over time, leading to differentiation inhibition and promotion of self-renewal (Figure 7). The latter process appears to be promoted when myogenesis occurs in the presence of the SC niche.

Our findings open up new questions regarding the molecular mechanisms that underlie p53-mediated regulation of the balance between differentiation and self-renewal. One possibility is that p53 regulates such balance via direct inhibition of myogenin expression (Yang et al., 2015) and consequently inhibition of differentiation, upregulation of PAX7, and activation of a program that resembles *in vivo* quiescence (Olguín and Olwin, 2004). By contrast, a direct upregulation of PAX7 by p53 appears unlikely (Yang et al., 2015). Interestingly, mice lacking the p53 target gene PW1 are born with fewer quiescent SCs (Nicolas et al., 2005). Since it is established that neonatal SCs are derived during development form MYOD+ myoblasts (Kanisicak et al., 2009), the finding that PW1 mutants lack quiescent SCs at birth further supports our view that p53 promotes generation of a population of quiescent SCs from proliferating myoblasts.

Although it had been previously shown that p53 inactivation via expression of a dominant-negative mutant decreases myoblast differentiation (Soddu et al., 1996) and that differentiation is decreased in primary *Trp53*^*−/−*^ myoblasts (Porrello et al., 2000), when we decreased p53 levels in myoblasts by transfecting a specific anti-p53 siRNA we did not observe altered differentiation in the absence of Nutlin-3. This might be due to the fact that we lowered serum levels prior to an effective knock down of p53 (the medium was switched only 3 hr after transfection). Indeed, also reserve cell generation was not affected by p53 knockdown in cultures that had not been treated with Nutlin-3. Thus, it is possible that a transient peak of p53 expression still occurred in cultures that had not been treated with Nutlin-3 prior to effective p53 knockdown by siRNA, thus ensuring a normal distribution between differentiation and quiescence. Vice versa, in cultures treated with Nutlin-3, p53 levels either rapidly increased in response to Nutlin-3 above the normal transient peak and remained high for the entire duration of the experiment (control siRNA), or, in the absence of Nutlin-3, were prevented from increasing further than the normal transient peak by p53 siRNA, and in this second case the distribution between differentiation and quiescence was maintained at basal levels. Thus, our data suggest that, after cell-cycle exit, the levels of p53 in the cell determine whether the cell is going to enter a terminal G0 phase and differentiate (p53 levels returning low; Figure 7), or whether the cell is going to remain in a reversible G0 phase and effectively renew the pool of undifferentiated, quiescent SCs (p53 levels further increasing and remaining high; Figure 7).

Overall our study supports a model whereby myogenic differentiation is the preferred fate choice once SCs have been removed from their anatomical niche and cultured as myoblasts, whereas the presence of the niche provides signals that promote quiescence and self-renewal via multiple mechanisms, one of which leads to p53 upregulation. Further research is necessary in order to identify the molecular mechanisms through which the SC niche regulates p53 protein levels in myoblasts; however, our results strongly support the notion that p53 plays an important physiological role in muscle stem cell biology.

## Experimental Procedures

### Mice

Male C57Bl/6J mice were purchased from Charles River and housed until used (at 12–13 weeks of age) in a pathogen-free facility at the University of Liverpool, in accordance with the Animals (Scientific Procedures) Act 1986 and the EU Directive 2010/63/EU, and after local ethical review and approval by the Animal Welfare and Ethical Review Body.

### Myofiber Cultures

Single myofibers were isolated and cultured as described previously (Pisconti et al., 2016). Further details can be found in Supplemental Experimental Procedures.

### Primary Dispersed Myoblast Cultures

Dispersed myoblasts were prepared and cultured as described previously (Pisconti et al., 2010). Further details can be found in Supplemental Experimental Procedures.

### C2C12 Cell Cultures

C2C12 myoblasts (Yaffe and Saxel, 1977) were cultured as described previously (Arecco et al., 2016). Further details can be found in Supplemental Experimental Procedures.

### Immunofluorescence

Myofibers, dispersed primary, and C2C12 myoblasts were fixed for either 20 min (myofibers) or 10 min (primary and C2C12 myoblasts) at room temperature with 4% paraformaldehyde prior to being processed for immunofluorescence as described previously (Pisconti et al., 2016). For a list of primary and secondary antibodies, the concentrations used and microscopy details, refer to Supplemental Experimental Procedures.

### Isolation of Myoblasts from Myofiber Cultures

Forty-eight and 72 hr after isolation, myofibers were collected, washed twice with PBS, always collecting the supernatant which contained loosely attached myoblasts, then treated with 0.05% trypsin for 10 min at 37°C. The action of trypsin was then stopped by addition of 2 volumes of primary SC growth medium and the myofibers separated from stripped myoblasts by centrifugation (3 min at 100 × *g*): the supernatant was collected and combined with the two previous PBS washes. Myoblasts were then collected by centrifugation at 500 × *g*, the pellet washed once in PBS, and then immediately lysed in RLT buffer (RNeasy Kit, QIAGEN). This method yielded highly pure preparations of myofiber-associated myoblasts (Figures S6A–S6E) and high-quality RNA (Figures S6F and S6G), which did not contain contaminant RNA from the myofiber (see Supplemental Information for details).

### RNA Extraction and Quality Control

RNA was extracted using the RNeasy Kit (QIAGEN) according to the manufacturer’s instructions. The quality and concentration of the RNA was assessed using a Bioanalyzer (Agilent 2100, Agilent) or a Nanodrop (Thermo Fisher).

### Microarrays

Sample labeling and microarray hybridizations were carried out according to Agilent's One-Colour Microarray-Based Gene Expression Analysis Protocol, v.6.6 (manual part number G4140-90040). Further details can be found in the Supplemental Experimental Procedures. Raw microarray data are available from the GEO public depository under the accession number: GEO: GSE109052.

### Microarray Analysis

Raw Agilent text files were read using the read. Agilent function in the marray R package (Yang, Paquet, and Dudoit, Exploratory analysis for two-color spotted microarray data. R package v.1.50.0. http://www.maths.usyd.edu.au/u/jeany/). Data were background corrected and loess normalized (using the normalizeWithinArrays function within the Limma package, followed by a quantile normalization using the normalizeBetweenArrays function) against their respective 48-hr data point to allow for a direct comparison between the two culture systems at both time points. Data were then summarized at the gene level by first averaging identical probes and then selecting a representative probe per gene name that showed the highest average intensity across the dataset. Genes with a median intensity level <5 across all arrays were removed. Differentially expressed genes were identified using the samr package (Tusher et al., 2001) for each of the three comparisons: (1) 72 versus 48 hr myofiber-associated myoblasts (Figure S2A), (2) 72 versus 48 hr dispersed myoblasts (Figure S2B), and (3) 72 hr/48 hr dispersed versus 72 hr/48 hr myofiber-associated myoblasts (Figure S2C). Genes significant at 1% FDR in each comparison were extracted and used for further analysis.

### Manual Annotation of Gene Families Involved in Myogenesis

Gene families involved in myogenesis were identified manually via screening of literature published in PubMed between 01/01/1986 and 01/01/2016. A gene family was annotated as being involved in myogenesis when at least one member of the family was described as involved in myogenesis in at least one peer-reviewed publication. This list included but was not limited to: myogenesis regulatory factors, Pax genes, skeletal muscle sarcomeric proteins, various extracellular matrix components, calcitonin receptor, Wnt pathway, Notch pathway, receptor tyrosine kinase pathways (fibroblast growth factor, hepathocyte growth factor, epidermal growth factor, insulin growth factor, etc.), transforming growth factor β family pathways, interleukins, cytokines, chemokines, mitogen-activated protein kinases, p53 family, cyclins, cyclin-dependent kinases, etc. The comparative analysis filtered through this manually curated list is reported in full in Table S3.

### Transfection

Myofiber and C2C12 cell cultures were transfected as described previously (Ghadiali et al., 2016). Further details can be found in Supplemental Experimental Procedures.

### Western Blotting

Primary and C2C12 myoblasts were lysed in modified RIPA buffer (50 mM Tris-HCl [pH 7.5], 150 mM NaCl, 1% IGEPAL, 0.5% SDS, and 0.1% sodium deoxycholate) and western blotting performed as described previously (Arecco et al., 2016). A list of primary and secondary antibodies and concentrations used can be found in the Supplemental Experimental Procedures. Quantitative analysis of western blots was performed using the “Analyze Gel” function of ImageJ.

### Statistical Analysis of Cell and Biochemistry Experiments

Experiments shown in Figures 3, 4, 5, and 6 were statistically analyzed using a t test as the data distributed normally. A p value < 0.05 was considered significant.

## Author Contributions

V.F. designed and performed experiments shown in Figures 1, 2, and S2. R.S.G. designed and performed experiments shown in Figures 4 and S3. P.A. performed the microarray analysis and the CR analysis. A.R. performed experiments shown in Figure 5. J.E.T. contributed to experimental design. A.P. contributed to experimental design and execution throughout the study and drafted the manuscript. All authors contributed to manuscript preparation by providing critical feedback.

## Supplemental Information

Document S1. Supplemental Experimental Procedures, Figures S1–S7, and Tables S1–S3

Document S2. Article plus Supplemental Information
