# Supplementary material for: The Satellite Cell Niche Regulates the Balance between Myoblast Differentiation and Self-Renewal via p53
Source: Stem Cell Reports. 2018 Feb 8;10(3):970–83. doi: 10.1016/j.stemcr.2018.01.007 (PMC5918193; doi:10.1016/j.stemcr.2018.01.007)
Supplement: Document S1. Supplemental Experimental Procedures, Figures S1–S7, and Tables S1–S3 [file mmc1.pdf]

Stem Cell Reports, Volume *10*

Supplemental Information

The Satellite Cell Niche Regulates the Balance between Myoblast Differentiation and Self-Renewal via p53

Valentina Flamini, Rachel S. Ghadiali, Philipp Antczak, Amy Rothwell, Jeremy E. Turnbull, and Addolorata Pisconti

The satellite cell niche regulates the balance between myoblast differentiation and self-renewal via p53

Valentina Flamini, Rachel S. Ghadiali, Philipp Antczak, Amy Rothwell, Jeremy E. Turnbull and Addolorata Pisconti

Supplementary Information

SUPPLEMENTARY METHODS

Myofibre cultures

Mice were culled under a CO_2 raising gradient and the gastrocnemius muscles dissected, incubated with 400 U/mL collagenase type I (*Worthington*) for 90 min then individual myofibres were picked using a glass Pasteur pipette whose tip had been flame-polished and flushed with fresh primary myoblast growth medium (F12 + 0.4 mM $CaCl_2$ + 15% horse serum + 1% penicillin + 1% streptomycin + 2 mM GlutaMax) in a cell culture incubator (humidified 37 °C, atmospheric O_2 and 5% CO_2). After 3 subsequent passages in fresh primary myoblast growth medium to remove debris and contaminant fibroblasts, FGF2 was added at the final concentration of 2 nM in the last passage. 2nM FGF2 was added again at 24 h and then the medium was changed once at 48 hours supplementing again with 2nM FGF2. Although it is still debated whether SCs derived from different myofibre types are also different and retain these different properties once cultured *ex vivo*, we chose to use the gastrocnemius muscle for myofibre preparations, rather than the most commonly used extensor digitorum longus (EDL) muscle, because the gastrocnemius muscle contains all myofibre types and therefore a myofibre-associated myoblast preparation obtained from the gastrocnemius muscle is more representative of all possible "types" of SCs than if the preparation was obtained from the EDL muscle, which contains almost exclusively fast twitch myofibres. This was especially important because the dispersed myoblast preparations were obtained from whole limb muscles and therefore they too contained all "types" of SCs. The same batch of horse serum was used throughout the work presented in this manuscript.

Myofibre-associated myoblast RNA quality control

We ran preliminary experiments to test for the presence of RNA from myofibre debris in our myofibre-associated myoblast preparations. We re-suspended the final pellet obtained after myoblast stripping into primary myoblast growth medium, plated this cell suspension on gelatin-coated dishes and the day after we extracted RNA from both the adherent cells and the supernatant. If significant amounts of contaminant RNA from the myofibres had been present in the final myoblast pellet, we should have been able to extract it and detect it from the supernatant of the plated myoblasts. Instead we only detected RNA from the adherent cell population not from the supernatant, which, once analysed by Bioanalyzer, showed only non-RNA contaminants (Fig. S7D-E), indicating that the final pellet obtained from myoblast isolation from myofibres is highly pure and does not contain myofibre RNA.

Preparation of dispersed myoblasts

Mice were culled under a CO_2 raising gradient and all the hind-limb muscles dissected, finely minced with scissors and then incubated with 400 U/mL collagenase type I for 60 minutes at 37 °C. After centrifuging at low speed (30 xg) for 3 minutes to pellet larger tissue debris, the supernatant containing mono-nucleated cells was collected, diluted with two volumes of primary myoblast growth medium (F12 + 0.4 mM $CaCl_2$ + 15% horse serum + 1% penicillin + 1% streptomycin + 2 mM GlutaMax) and then centrifuged again at 500 xg to pellet the mono-nucleated cells. Fibroblasts were removed via two subsequent pre-plating steps (2h + 1h, each one on gelatin-coated dishes, at humidified 37 °C, atmospheric O_2 and 5% CO_2). After the second pre-plating the floating cells were plated on gelatin-coated dishes in primary myoblast growth medium, supplemented with 2 nM FGF2 and incubated for 48 or 72 hours, with one medium change at 48 hours. This protocol yielded a preparation of

great purity (over 95% MYF5+/PAX7+ cells). For the microarray experiments, at 48 and 72 h after plating, cells where washed with phosphate buffered saline (PBS) and lysed with RLT buffer (Qiagen) prior to RNA extraction. For the reserve cell assays, 48 h after plating cells were washed twice with F12C (F12 + 0.4 mM $CaCl_2$) and then switched to primary myoblast low serum medium (F12 + 0.4 mM $CaCl_2$ + 3% horse serum + 1% penicillin/streptomycin + 2 mM GlutaMax) for 3 days. After 3 days, cells were either fixed and immunostained or "washed" with F12C and incubated again in primary myoblast growth medium (containing 15% horse serum) for 2 days, prior to being fixed and immunostained. The same batch of horse serum was used throughout the work presented in this manuscript.

C2C12 myoblast cultures

C2C12 myoblasts (Yaffe and Saxel, 1977) were cultured in C2C12 growth medium (DMEM + 10% foetal bovine serum + 2 mM L-glutamine + 1% penicillin + 1% streptomycin) up to 90% confluence then washed twice with DMEM and switched to C2C12 low serum medium (DMEM + 3% horse serum + 2 mM L-glutamine + 1% penicillin + 1% streptomycin) to induce differentiation and reserve cell generation (Yoshida et al., 1998) in the presence/absence of either 20 μM Nutlin-3 (Sigma Aldrich) or an equal volume of vehicle (DMSO, Sigma Aldrich) for an additional 3 days. After 3 days, cells were either immediately fixed and immunostained or "washed" with DMEM and re-incubated in C2C12 growth medium (containing 10% foetal bovine serum) for an additional 2 days prior to being fixed and immunostained. The same batch of foetal bovine serum was used throughout the work presented in this manuscript.

Antibodies

For immunofluorescence, primary antibodies used were: mouse anti-Pax7 (DSHB) 1:100, mouse anti-p53 (Cell Signaling, Cat No. 2524) 1:100, rabbit anti-myogenin (SCBT, Cat. No. sc-576) 1:500, rabbit anti-SDC3 (kindly donated by Prof Brad Olwin, University of Colorado (Cornelison et al., 2001) 1:500, rabbit anti-KI67 (Abcam, Cat. No. ab15580) 1:400, mouse anti-myogenin (DSHB, clone F5D) 1:100, mouse anti-myosin heavy chain (DSHB, clone MF20) 1:100, rabbit anti-MYF5 (SCBT, Cat. No. sc-302) 1:500, rabbit anti-MYOD (SCBT, Cat. No. sc-760) 1:600. Secondary antibodies conjugated to Alexa488, Alexa555 or Alexa647 (Invitrogen) were used at 1:500. DAPI (Invitrogen) was used at 2 μM in PBS.

For western blotting, primary antibodies used were: mouse anti-p53 (Cell Signaling, Cat No. 2524) 1:1,000; mouse anti-GAPDH (Sigma Aldrich, Cat. No. G8795) 1:3,000; mouse anti-myogenin (DSHB, clone F5D) at 1:1,000 and mouse anti-sarcomeric myosin (DSHB, clone MF20) at 1:3,000. Secondary antibodies were from Santa Cruz Biotechnology and used at 1:10,000. Chemiluminescence was detected on a LAS 4,000 (GE) image doc system.

Microscopy

Microscope images were acquired on an EVOS FL imaging system (Life Technologies) with a 10X, a 20X or a 40X objective and consistent imaging parameters.

Microarrays

Fluorescently labelled amplified complementary RNA (cRNA) was generated using a Low Input Quick Amp Labelling kit, One-Colour (Agilent). The method utilises an oligo (dT) primer bearing a T7 promoter, and MMLV-RT, producing double stranded

cDNA from mRNA; the cDNA then serves as template for *in vitro* transcription with T7 RNA Polymerase, which linearly amplifies target material whilst simultaneously incorporating cyanine 3-labelled CTP. For each labelling reaction, 70 ng of total RNA was used as input, along with appropriately diluted Spike Mix from the One-Colour RNA Spike-In kit (Agilent). cRNA was purified using an RNeasy Mini Kit (Qiagen) and quantified on a NanoDrop ND-1000 Spectrophotometer version 3.3.0. For array hybridisation, 600 ng of each cyanine 3-labelled cRNA, was combined with 5 µl of 10X Gene Expression Blocking Agent and 1 µl of 25X Fragmentation Buffer (both from the Agilent Gene Expression Hybridisation kit) in a total volume of 25 µl. Target mixtures were then incubated at 60 °C for 30 min to fragment the RNA to approximately 150 nucleotides. Fragmentation was terminated by cooling on ice for 1 min followed by addition of 25 µl of 2X Hi-RPM Hybridisation Buffer (Agilent). Agilent SurePrint 8X 60K Mouse Gene Expression microarrays (Design ID 028005) were loaded and hybridised using Agilent hardware, namely: gasket slides, SureHyb chambers and hybridisation oven. Hybridisation was carried out at 65 °C with 10 rotations per minute for 17 h. After this time, microarrays were washed using an Agilent Gene Expression Wash Buffer Kit according to the manufacturer's instructions. Arrays were scanned immediately at a resolution of 3 µm using an Agilent DNA Microarray Scanner to generate 20 bit tiff images. Data was extracted and QC reports generated using Agilent Feature Extraction version 11.0.1.1. Array quality was assessed by visual inspection of each tiff and analysis of the associated QC report, which indicates the dynamic range of the experiment, hybridisation and background uniformity, as well as an evaluation of metrics associated with the RNA spike-ins added to the labelling reactions.

Transfection

C2C12 myoblast cultures that had reached 90% confluence or myofibre cultures were washed twice with PBS and switched to OptiMem before receiving a 1:3, volume:volume mixture of siRNA (diluted in OptiMem for a final 30 or 60 nM):Lipofectamine2000. After addition of the siRNA:Lipofectamine2000 mixture, plates where incubated for three hours in a cell culture incubator (humidified 37 °C, atmospheric O_2 and 5% CO_2), then the medium was replaced with low serum medium that contained either 20 µM Nutlin-3 or an equal volume of DMSO and incubated for another day (for western blot analysis of C2C12 cells) or three days (for immunofluorescence analysis of C2C12 cells) or 2 days (for immunofluorescence analysis of myofibre cultures). siRNAs were either control (scrambled) siRNA (Sigma Aldrich) or anti-p53 siRNA (Sigma Aldrich, SASI_Mm02_00310137).

SUPPLEMENTARY FIGURES

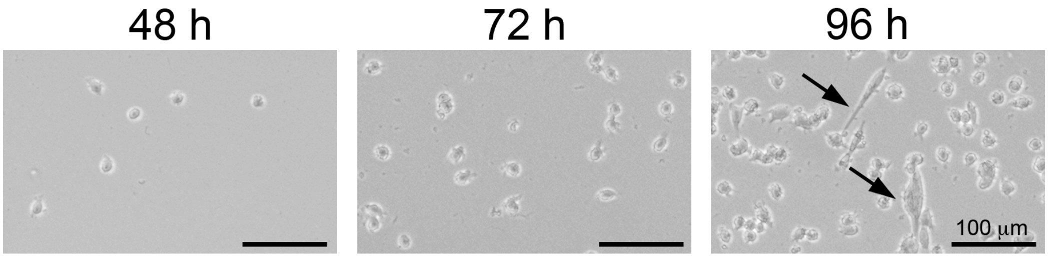

<u>Supplementary Figure S1 – supplementing Fig. 1A-B</u>: **Dispersed myoblasts cultured on gelatin in the presence of high serum and FGF2 undergo spontaneous differentiation.** Primary SC-derived myoblasts were cultured on gelatin-coated plates in primary myoblast growth medium supplemented with 2 nM FGF2 for 4 days. During the first 2-3 days cells mostly proliferated, around the third day they underwent spontaneous differentiation such that myotubes were visible at day 4 (black arrows).

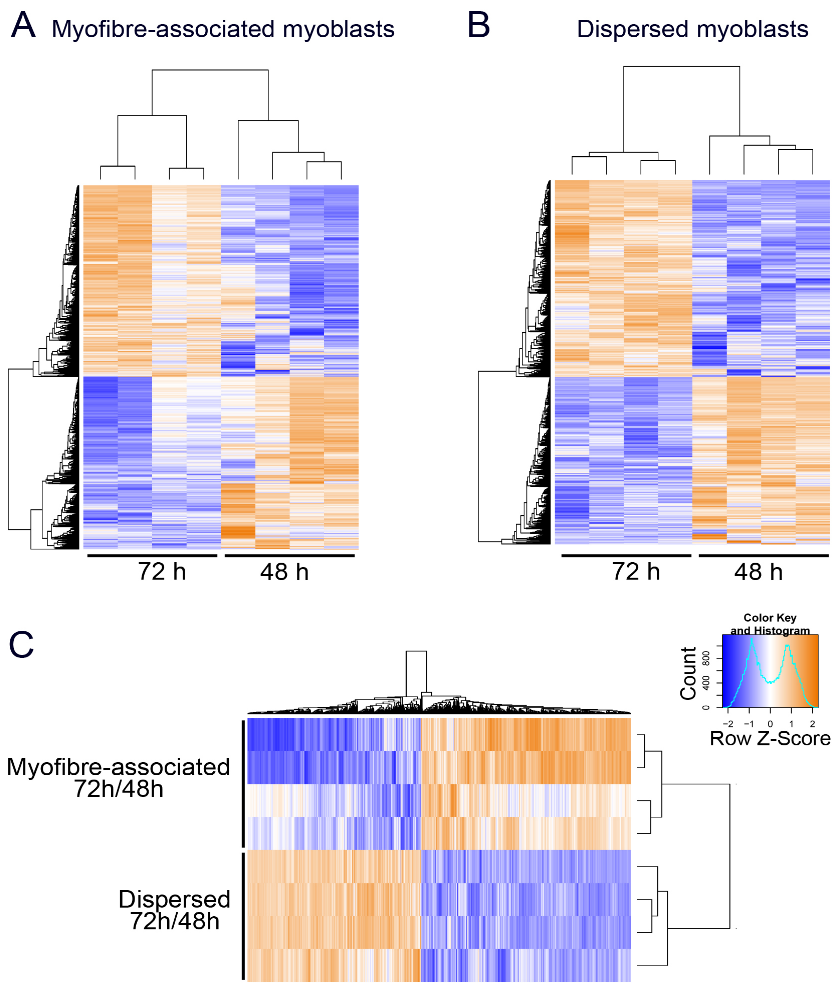

<u>Supplementary Figure S2 – supplementing Fig. 1E</u>: Replicate reproducibility was excellent in both dispersed and myofibre-associated myoblast cultures, which appeared very different in their transcriptome signature during myogenic progression. A-B) Hierarchical clustering of the Top 200 differentially expressed genes between 48 and 72 hours in culture in myofibre-associated (A) and dispersed (B) myoblasts. For each condition the biological replicates clustered together suggesting high reproducibility and high quality of the data. Moreover, as visually shown by the heatmaps, the transcriptomic signature at 48 hours is very different from the transcriptomic signature at 72 hours for both culture conditions. **C)** Gene expression regulation between 48 and 72 hours in myofibre-associated myoblasts and in dispersed myoblasts are different.

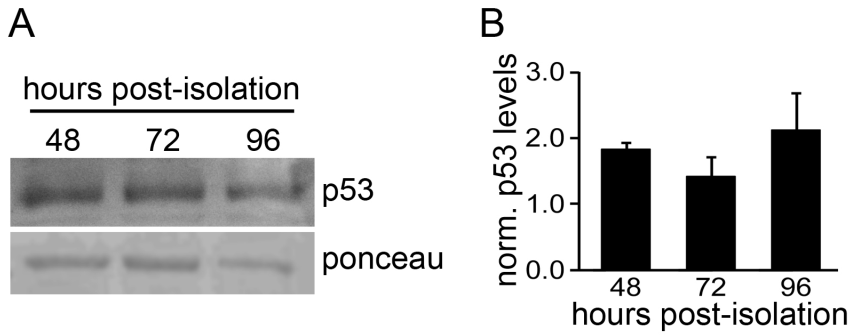

<u>**Supplementary Figure S3 – supplementing Fig. 3**</u>**: p53 protein levels do not significantly increase over time in primary dispersed myoblast cultures. A-B)** Primary dispersed myoblasts were cultured for up to 96 hours post-isolation, lysed at three time points: 48, 72 and 96 hours, then total proteins extracted and analysed by western blot to detect p53 levels. Ponceau staining was used as loading control. A representative western blot image is shown in (A) while (B) is the quantification of three independent experiments.

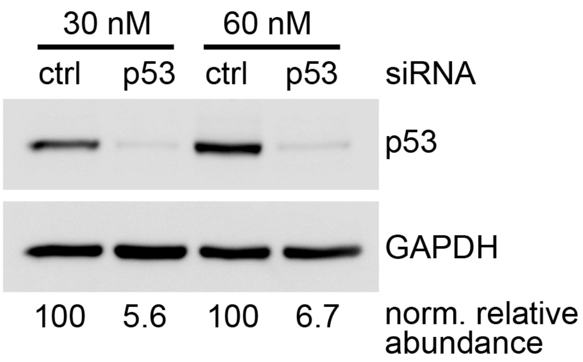

Supplementary Figure S4 – supplementing Fig. 4: siRNA-mediated p53 knockdown is highly efficient. C2C12 myoblast were cultured in growth medium until they reached 90% confluence then transfected with either control siRNA (30 nM, lane 1; 60 nM, lane 3) or anti-p53 siRNA (30 nM, lane 2; 60 nM, lane 4), 3 h later switched to low serum medium supplemented with either DMSO or 20 µM Nutlin-3 and cultured for an additional 24 h prior to being lysed and analysed by blot to reveal p53 (top gel) and GAPDH (bottom gel) abundance. The intensity of the p53 bands normalised to the intensity of the GAPDH bands are reported below the gel images as percentage of the control band for each one of the two siRNA concentrations tested. Since the level of knockdown was comparable between 30 nM and 60 nM siRNA, all subsequent experiments were carried out using 30 nM siRNA.

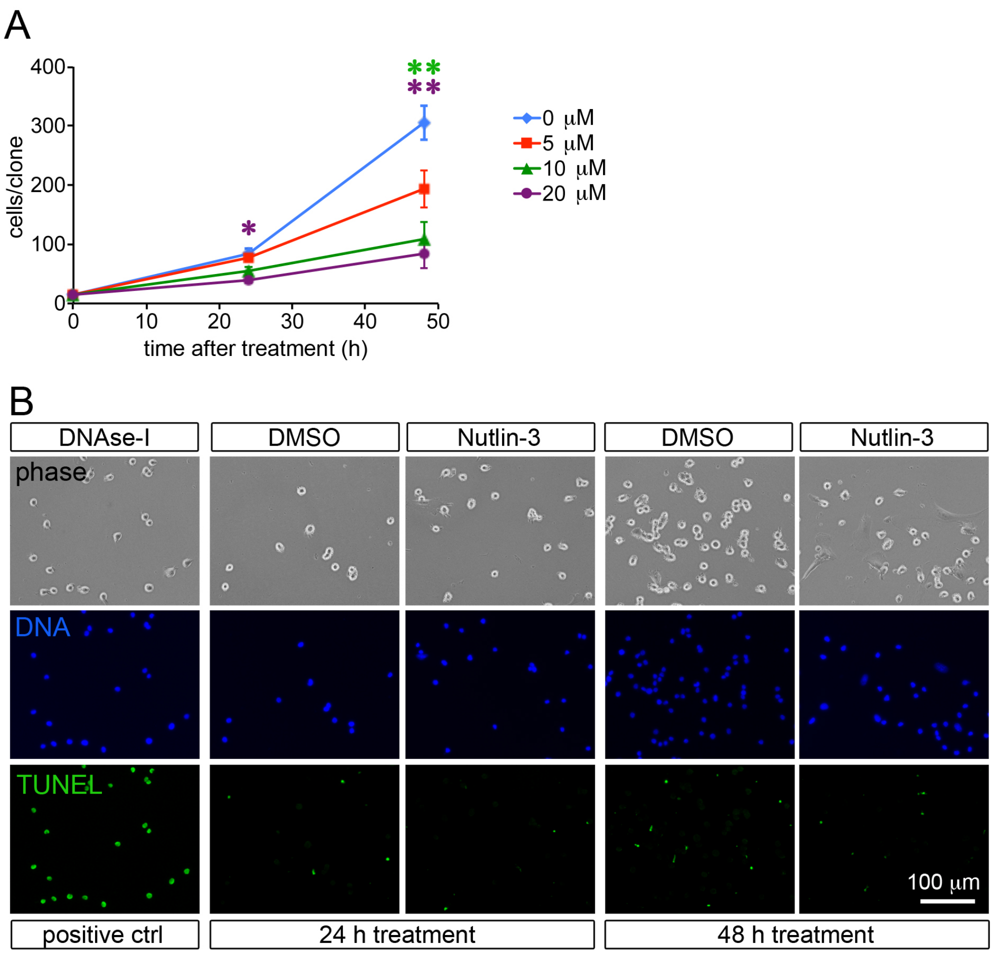

Supplementary Figure S5 – supplementing Fig. 5: Nutlin-3 treatment of primary dispersed myoblasts inhibits proliferation without increasing cell death. A) Primary dispersed myoblasts were expanded for two days prior to being detached and re-plated at clonal density for an additional three days. The day after plating (time 0 treatment) culture medium was supplemented with either DMSO or Nutlin-3 at 3 different concentrations: 5 µM, 10 µM or 20 µM. Samples were fixed either the day after (24 h treatment) or 2 days later (48 h treatment) and the numbers of cells/clone scored and plotted as average across 3 independent experiments ± S.E.M. At 24 h, only 20 µM Nutlin-3 significantly reduces proliferation (* = $p < 0.05$), at 48 h both 10 µM and 20 µM Nutlin-3 significantly reduce proliferation (** = $p < 0.01$). **B)** Primary dispersed myoblasts were plated and expanded for one day before being treated with either DMSO or 20 µM Nutlin-3, then fixed either the day after (24 h treatment) or 2

days later (48 h treatment), processed for TUNEL assay (green) and counter-stained with DAPI to detect DNA (blue). At each time point, a non-treated control was also fixed and treated with DNAse-I before TUNEL assay to induce DNA breakage – this is labelled as DNAse-I/positive control. Although in the positive control all cells are TUNEL+ indicating that the TUNEL assay worked, in both the DMSO and Nutlin-3-treated samples, at both time points, only very few TUNEL+ spots are detected, which do not co-localise with DAPI staining (hence the lack of quantification) and might therefore be an artifact or might be late apoptotic cells whose DNA was so extremely fragmented that no DAPI staining was detectable any more. In any case, there appear to be no difference between the DMSO-treated and the Nutlin-3-treated samples.

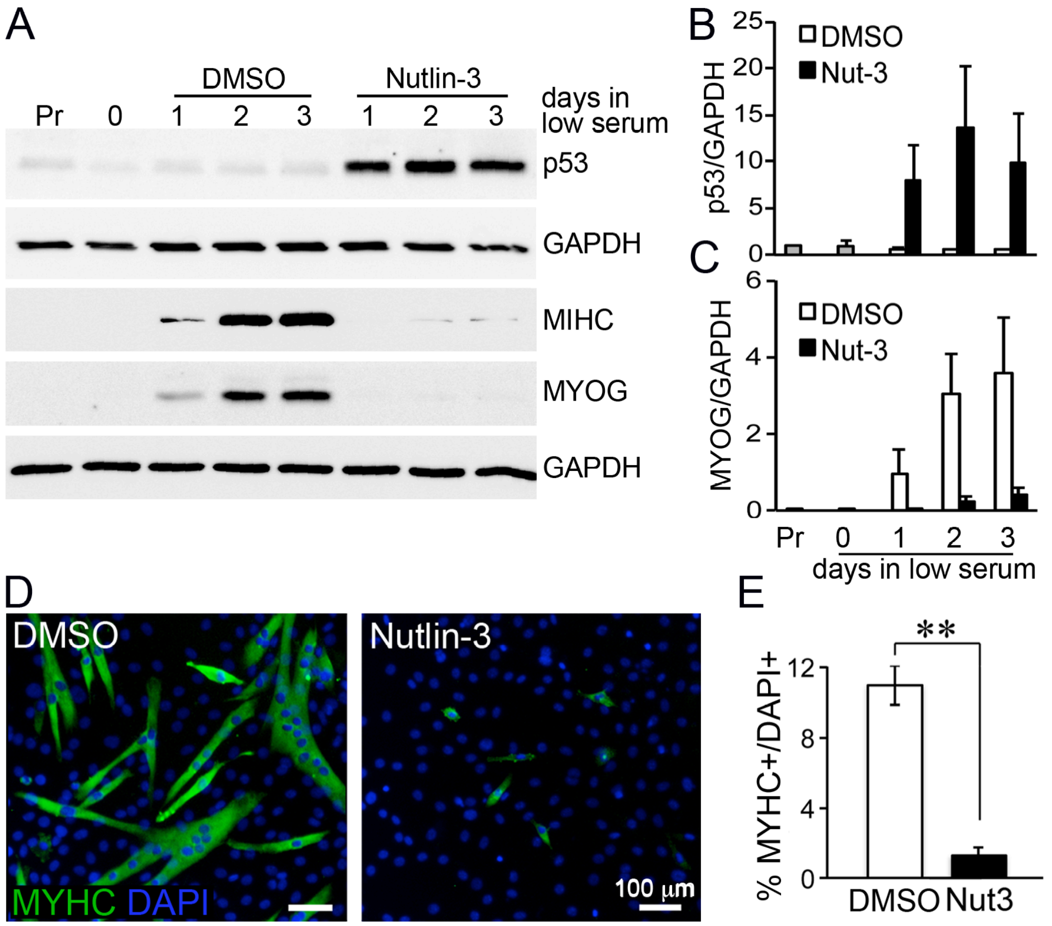

<u>Supplementary Figure S6 – supplementing Fig. 5 and Fig. 6</u>: A sustained increase in p53 levels reduces C2C12 myoblast differentiation. A-C) C2C12 myoblast cultures were grown to 90% confluence then switched to low serum medium (DMEM + 3% horse serum + 1% pen/strep + 2 mM L-glutamine) either in the presence of 20 µM Nutlin-3 or in the presence of an equal volume of DMSO and lysed at the following time points: Pr (= proliferating) is the day before reaching 90% confluence; 0 is the day when 90% confluence was reached; 1, 2 and 3 are the days subsequent to medium switch. At each time point cells were lysed and then all time points loaded onto a 10% SDS-PAGE for western blot analysis to reveal the abundance of myosin heavy chain (MYHC), myogenin (MYOG), p53 and GAPDH. Representative gels from three independent experiments are shown in (A) where to top GAPDH gel is related to the p53 gel, while the bottom GAPDH gel is related to

the MYHC and MYOG gel. In (B-C) is the quantification of three independent experiments carried out as in (A) where the average band intensity for p53 (B) or MYHC (C) normalised to the band intensity for its related GAPDH at each time point is plotted. Error bars are S.E.M. across the three independent experiments. **D-E)** Immunofluorescence analysis of C2C12 myoblasts switched to low serum medium when 90% confluence was reached and treated with either 20 μM Nutlin-3 or DMSO for 3 days prior to fixation and immunostaining to detect MYHC (green) and DNA (DAPI, blue) reveals that differentiation is reduced by Nutlin-3 treatment. In (D) one representative of several (> 10) independent experiments is shown. In (D) quantitation of three independent experiments across 10 technical replicates for each one of three biological replicates (N = 30) as the percentage of MYHC+ cells over the total numbers of DAPI+ cells is plotted. Error bars are S.E.M. ** = $p < 0.01$.

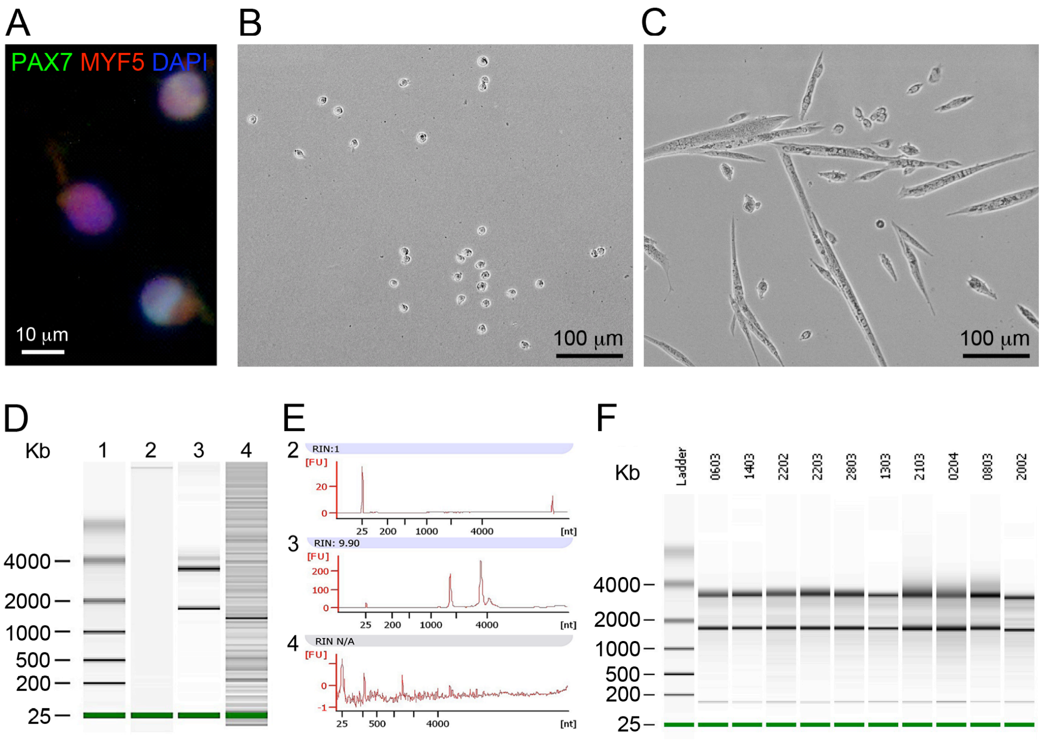

G

Sample name	Myoblas Prep	RNA extraction and purification method	Quantification method	Concentration (ng/ul)	RIN	NanoDrop 260/280 ratio
0603 (72h)	myofibre	Qiagen RNeasy kit	Bioanalyser	32.3	8.2	N.A.
1403 (72h)	myofibre	Qiagen RNeasy kit	Bioanalyser	15.7	8.9	N.A.
2202 (72h)	myofibre	Qiagen RNeasy kit	Bioanalyser	17.3	7.7	N.A.
1303 (72h)	myofibre	Qiagen RNeasy kit	Bioanalyser	8	7.5	N.A.
2203 (48h)	myofibre	Qiagen RNeasy kit	Bioanalyser	3.83	8.6	N.A.
2803 (48h)	myofibre	Qiagen RNeasy kit	Bioanalyser	7.7	8	N.A.
2103 (48h)	myofibre	Qiagen RNeasy kit	Bioanalyser	8.4	8.8	N.A.
0204 (48h)	myofibre	Qiagen RNeasy kit	Bioanalyser	11.6	7.4	N.A.
2002 (48h)	myofibre	Qiagen RNeasy kit	Bioanalyser	4.02	7.4	N.A.
1604 (48h)	myofibre	Qiagen RNeasy kit	Bioanalyser	3.2	8.7	N.A.
S1 (48h)	dispersed	Qiagen RNeasy kit	Nanodrop	12.2	N.A.	2.2
S2 (72h)	dispersed	Qiagen RNeasy kit	Nanodrop	22.0	N.A.	2.2
S3 (48h)	dispersed	Qiagen RNeasy kit	Nanodrop	25.0	N.A.	2.1
S4 (72h)	dispersed	Qiagen RNeasy kit	Nanodrop	32.9	N.A.	2.2
S5 (48h)	dispersed	Qiagen RNeasy kit	Nanodrop	35.6	N.A.	2.1
S6 (72h)	dispersed	Qiagen RNeasy kit	Nanodrop	39.2	N.A.	2.1
S7 (48h)	dispersed	Qiagen RNeasy kit	Nanodrop	47.2	N.A.	2.2
S8 (72h)	dispersed	Qiagen RNeasy kit	Nanodrop	66.6	N.A.	2.1

Supplementary Figure S7 – cited in *Experimental Procedures*: Validation of the protocol to isolate myofibre-associated myoblasts. A) Myofibre-associated myoblasts were stripped off myofibres using trypsin as described in the *Experimental Procedures* section then cytospun and immunostained to detect the SC/myoblast markers PAX7 and MYF5: 100% of the cells cytspun were positive for at least one of these two markers. **B-C)** Isolated myofibre-associated myoblasts were tested for viability and functionality by plating them on gelatin-coated dishes in primary

myoblast growth medium. Three days later isolated myofibre-stripped myoblasts had extensively proliferated in culture (B) and after switching them to low serum medium they underwent differentiation into myotubes (C). **D-E)** Bioanalyzer analysis of the RNA extracted from: negative control (water, lane 2); adherent cells (lane 3); supernatant of isolated myofibre-associated myoblasts plated on gelatin-coated dishes immediately after isolation (lane 4). Please not the massive differences in the Y-axis scales in the chromatograms shown in (E). This analysis reveals that no RNA was present in the supernatant indicating that no RNA was carried over from myofibre debris into the RNA preparations that were used for microarray analysis. **F)** Bioanlyzer analysis of the RNA extracted from all the biological replicates of myofibre-associated myoblasts at 48 hours and 72 hours post-myofibre isolation. Only for one of the 4 replicates of myofibre-associated myoblasts at 48 h, two distinct preparations were pooled to produce one biological replicate. In all other cases each biological replicate corresponded to one independent preparation. **G)** Table reporting, for each RNA sample used in the microarray experiment: sample name, type of myoblast prep, system for preparation, method of quantification, concentration, RIN where applicable and 260/280 ratio where applicable. All RNA samples were eluted in the same volume (14 μL) therefore the concentrations reported are a direct measure of the total amount of RNA extracted.

REFERENCES

Cornelison, D.D., Filla, M.S., Stanley, H.M., Rapraeger, A.C., Olwin, B.B., 2001. Syndecan-3 and syndecan-4 specifically mark skeletal muscle satellite cells and are implicated in satellite cell maintenance and muscle regeneration. Dev Biol 239, 79–94. doi:10.1006/dbio.2001.0416

Yaffe, D., Saxel, O., 1977. Serial passaging and differentiation of myogenic cells isolated from dystrophic mouse muscle. Nature 270, 725–727.

Yoshida, N., Yoshida, S., Koishi, K., Masuda, K., Nabeshima, Y., 1998. Cell heterogeneity upon myogenic differentiation: down-regulation of MyoD and

Myf-5 generates “reserve cells.” J Cell Sci 111 (Pt 6), 769–779.

Supplementary Table 1 – Cited in Results, section 4: Top three GO terms that are most significantly enriched in genes with CR > 2.

Term	p-value	Genes	Fold enrichment
Negative regulation of transport	0.00002	*Lif, Il6, Nos1, Snph, Edn1, Pkia, Adora1, Htr2a*	8.99
Striated muscle development	0.00004	*Musk, Actc1, Myl2, Tnc, Myog, Ttn, Neurl2*	10.48
Taxis	0.00005	*Ccr8, Ccl2, Cxcl3, Cxcl2, Ecscr, Amot, Ccl5, Ccl7, Slit2*	6.68

Supplementary Table 2 – Cited in Results, section 4: Top three GO terms that are most significantly enriched in genes with CR < 0.5.

Term	p-value	Genes	Fold enrichment
Cell adhesion	0.0004	*Col18a1, Selp, Pcdhb7, Pcdhb6, Pcdhb3, Pcdhb4, Itgb4, Cdhr5, Vtn, Pcdh17, Megf10, Dchs1, Cldn15, Aplp1, Wnt7b, Lamb3, Itga6, Lama5, Tro, Otog, Msln*	2.42
Cell morphogenesis involved in differentiation	0.0016	*Col18a1, Ablim1, Sema6a, Notch1, Slc1a3, Rtn4rl1, Cxcr4, Sema3a, Gas1, Ngfr, Cxcl12*	3.35
Cell fate commitment	0.0019	*Notch3, Fgfr4, Notch1, Tbx2, Pax7, Pparg, Cyp26b1, Gas1, Sox8*	3.96

Supplementary Table 3 – *Cited in Experimental Procedures and in Results, section 4*: Comparative analysis of genes that change between 48 and 72 hours according to the formula: $[(72h/48h)_{dispersed}]/[(72h/48h)_{myofibre\text{-}associated}]$ filtered through a manually curated list of genes involved in myogenesis.

Gene Name	Fold Change	q-value
Diras2	0.054013395	0.0000000
Mc4r	0.093310186	0.0000000
Aqp5	0.096597149	0.0000000
Ngfr	0.105205253	0.0000000
Cd5l	0.179134772	0.0000000
Sema6b	0.228863483	0.0000000
Trpc3	0.233711819	0.0235839
Fzd6	0.235680061	0.0000000
Fgfr4	0.236169718	0.0000000
Epha1	0.250161594	0.0000000
Itgb4	0.25121833	0.0000000
Pitx3	0.255210552	0.0000000
Itga6	0.263171022	0.0000000
Notch1	0.277726352	0.0000000
Col18a1	0.278990429	0.0000000
Tmem119	0.290233444	0.0000000
Notch3	0.291949448	0.0000000
Col10a1	0.300396372	0.0000000
Cxcr4	0.315090189	0.0000000
Cdkn2a	0.316454481	0.0000000
Ly6a	0.316960439	0.0000000
Sox8	0.323270186	0.0000000
Tmem44	0.328784523	0.0000000
Megf10	0.335582833	0.0000000
Cdc42ep1	0.337906165	0.0000000
Hes6	0.338134183	0.0000000
Pax7	0.344352698	0.0000000
Hey1	0.345761151	0.0000000
Cxcl12	0.348607325	0.0000000
Sema6a	0.349215321	0.0000000
Rnf152	0.350092944	0.0000000
Tmem191c	0.350341532	0.0235839
Pparg	0.361863203	0.0000000
Rnf144a	0.400662004	0.0000000
Cdkn2b	0.401434376	0.0000000
Lama5	0.412932128	0.0000000
Sema3a	0.41859219	0.0000000
Tcf7	0.423415284	0.0000000
Pdgfc	0.427927325	0.0235839
Tmem176a	0.428260942	0.0235839
Jam2	0.43211217	0.0000000
Pdgfrl	0.436975917	0.0000000
Sdc3	0.437947492	0.0000000

Timp4	0.442403723	0.0000000
Cnnm2	0.442786114	0.0000000
Mmp15	0.449408686	0.0000000
Rnf125	0.454254155	0.0000000
Lamb3	0.455321565	0.0000000
Rnf208	0.463874638	0.0000000
Tnfaip8l1	0.465211468	0.0000000
Tmem30b	0.471209484	0.0235839
Cd200	0.472625308	0.0000000
Fzd2	0.481798531	0.0000000
Ace	0.485118212	0.0000000
Lrp12	0.487980149	0.0000000
Wnt7b	0.491717209	0.0000000
Gper	0.493160426	0.0000000
Creb3l1	0.50032382	0.0000000
Tcf4	0.502701894	0.0000000
Tnfrsf25	0.511265133	0.0235839
Trim62	0.511388518	0.0000000
Megf9	0.512288348	0.0235839
Tmem176b	0.512579672	0.0335022
Chrna1	0.512895398	0.0000000
Ephb4	0.513271937	0.0000000
Cdh15	0.513597528	0.0000000
Igfbp4	0.518688547	0.0000000
Megf6	0.520352228	0.0335022
Tmem195	0.524521151	0.0000000
Cd276	0.526542761	0.0000000
Traf5	0.534764828	0.0235839
Map3k11	0.541734759	0.0000000
Cd2ap	0.550591348	0.0000000
Tmem121	0.56017007	0.0235839
Smad6	0.56064096	0.0000000
Tmem9	0.562422561	0.0000000
Il20rb	0.563193529	0.0000000
Tmem229b	0.571979361	0.0000000
Wnt6	0.579395389	0.0000000
Ctnnb1	0.58218307	0.0235839
Pth1r	0.583389597	0.0000000
Hspa12b	0.58409558	0.0000000
Il16	0.587001345	0.0235839
Cdc42ep4	0.591780882	0.0000000
Cd97	0.595509301	0.0000000
Jak3	0.600945954	0.0000000
Cd38	0.603198555	0.0235839
Tmem86a	0.606710437	0.0000000

Il18rap	0.608983054	0.0335022
Mmp11	0.61109568	0.0000000
F2r	0.618226226	0.0000000
Cdk19	0.618693237	0.0000000
Il34	0.619225504	0.0000000
Mmp17	0.62185378	0.0235839
Map4k2	0.623623769	0.0000000
Cxcr7	0.624860007	0.0000000
Hmgb3	0.624875968	0.0000000
Tmem173	0.630723339	0.0000000
Six2	0.631694902	0.0335022
Ctnnbip1	0.63742738	0.0335022
Fzd3	0.639107577	0.0335022
Socs2	0.64750778	0.0335022
Hmgn5	0.648216068	0.0235839
Stat2	0.658367534	0.0235839
Cdk2ap2	0.659850247	0.0000000
Met	0.665855282	0.0000000
Sdc4	0.667108623	0.0235839
Tcf3	0.668449518	0.0000000
Trim12	0.671382361	0.0335022
Ilkap	0.675550964	0.0335022
Ccna2	0.675932308	0.0235839
Mapk3	0.686093157	0.0000000
Itga3	0.688256775	0.0000000
Ilf2	0.689515315	0.0000000
Grb10	0.691704377	0.0000000
Tmem110	0.694182038	0.0335022
Itm2c	0.701841327	0.0335022
Cd3eap	0.704417076	0.0335022
Rnf38	0.704939689	0.0335022
Nfkbil2	0.705436177	0.0335022
Map3k12	0.711453353	0.0235839
Mybl2	0.712538267	0.0235839
Cdh8	0.733437491	0.0235839
Cdkn2c	0.733704766	0.0235839
Cd151	0.734003656	0.0235839
Casp2	0.740118071	0.0335022
Cdk10	0.742337344	0.0235839
Atoh8	0.743759899	0.0235839
Cdk2ap1	0.759884675	0.0335022
Tmem179b	0.790983217	0.0335022
Tmem50a	0.791326965	0.0335022
Col9a2	0.805013649	0.0235839
Nos2	0.808038002	0.0235839

Rnf160	1.182986633	0.0335022
Ppara	1.226111384	0.0235839
Rnf103	1.265119029	0.0000000
Abl2	1.28007608	0.0235839
Rnf181	1.28600617	0.0335022
Sod2	1.288438354	0.0235839
Tmem126a	1.297795278	0.0335022
Large	1.304309246	0.0335022
Tmem9b	1.31584281	0.0335022
Rnf170	1.32832226	0.0235839
Lamc1	1.338170117	0.0335022
Crybg3	1.352691383	0.0235839
Rnf19b	1.377284374	0.0335022
Nfix	1.428716223	0.0335022
Cd300lg	1.487985697	0.0335022
Itga2b	1.518339592	0.0335022
Cdc42ep3	1.520005509	0.0335022
Trim23	1.53151972	0.0335022
Cdkn1c	1.568324588	0.0235839
Ccng1	1.608196047	0.0335022
Tmem143	1.681525317	0.0000000
Ar	1.706092125	0.0235839
Mef2d	1.71779398	0.0000000
Tmem51	1.720317928	0.0335022
Myc	1.727137888	0.0235839
Tmem14a	1.741341997	0.0000000
Rnf113a2	1.748848333	0.0000000
Traf3	1.749643353	0.0000000
Rnf128	1.758049346	0.0000000
Col4a5	1.764058974	0.0235839
Hes2	1.807606777	0.0335022
Rnf123	1.822805482	0.0335022
Itga5	1.824026319	0.0335022
Tmem65	1.844739221	0.0000000
Mapk14	1.877026513	0.0000000
Tmem25	1.877475243	0.0000000
Tmem170	1.890353302	0.0335022
Myh7	1.904240751	0.0335022
Hdac9	2.039262001	0.0335022
Cd93	2.077909983	0.0335022
Ang2	2.119929417	0.0000000
Fgf10	2.136513435	0.0335022
Rnf39	2.142887692	0.0235839
Rnf115	2.185271495	0.0335022
Tmem74	2.189673882	0.0235839

Wnt5a	2.225643565	0.0000000
Tgfbr3	2.243237532	0.0335022
Nos1	2.244337119	0.0000000
Tnfaip3	2.370856078	0.0335022
Tmem22	2.382163203	0.0335022
Timp3	2.389738255	0.0000000
Grb14	2.450073063	0.0335022
Icam1	2.480875001	0.0000000
Cd34	2.502741332	0.0235839
Fgf13	2.522457048	0.0335022
Tnfrsf26	2.526705886	0.0335022
Cxcl1	2.571387986	0.0335022
Rnf150	2.613735241	0.0000000
Cxcr6	2.666414714	0.0235839
Hspb1	2.671942324	0.0335022
Igf2bp1	2.685073278	0.0335022
Lama4	2.694176556	0.0000000
Col11a1	2.81007029	0.0000000
Il1rl1	2.883004106	0.0235839
Angpt2	2.889406621	0.0000000
Ctgf	2.917124574	0.0000000
Trim55	2.943127311	0.0235839
Sema3c	2.962051241	0.0000000
Itga11	3.097928862	0.0335022
Smad3	3.143103306	0.0335022
Itgb7	3.180213711	0.0000000
Des	3.276458094	0.0335022
Wnt9a	3.473096788	0.0000000
Tmem132e	3.637282601	0.0000000
Tnfaip6	3.705210584	0.0000000
Crym	3.715182105	0.0335022
Pdgfra	3.799874363	0.0000000
Tmem56	3.824028259	0.0000000
Stac3	4.096755699	0.0000000
Itga2	4.151801886	0.0000000
Myl2	4.478819312	0.0000000
F3	4.53209333	0.0000000
Itgb1bp2	5.052382919	0.0000000
Cxcl2	5.21740209	0.0000000
Il6	5.883417321	0.0000000
Cxcl5	7.072889179	0.0335022
Mmp10	8.091003904	0.0000000
Mmp3	9.798800323	0.0000000
Csf2	10.41366572	0.0000000
Postn	13.13746879	0.0000000

Fgf21	13.98192167	0.0000000
Cxcl3	15.22060208	0.0000000
Myl3	18.83977572	0.0000000
Myog	20.03261426	0.0000000
Mmp13	28.20644517	0.0000000
