# Supplementary material for: The Satellite Cell Niche Regulates the Balance between Myoblast Differentiation and Self-Renewal via p53
Source: Stem Cell Reports. 2018 Feb 8;10(3):970–83. doi: 10.1016/j.stemcr.2018.01.007 (PMC5918193; doi:10.1016/j.stemcr.2018.01.007)
Supplement: Document S2. Article plus Supplemental Information [file mmc2.pdf]

Stem Cell Reports
Article
OPEN ACCESS

The Satellite Cell Niche Regulates the Balance between Myoblast Differentiation and Self-Renewal via p53

Valentina Flamini,[1] Rachel S. Ghadiali,[1] Philipp Antczak,[2,3] Amy Rothwell,[1] Jeremy E. Turnbull,[1] and Addolorata Pisconti[1,*]

[1]Department of Biochemistry, Institute of Integrative Biology, University of Liverpool, Liverpool L69 7ZB, UK
[2]Department of Functional Genomics, Institute of Integrative Biology, University of Liverpool, Liverpool L69 7ZB, UK
[3]Computational Biology Facility, Institute of Integrative Biology, University of Liverpool, Liverpool L69 7ZB, UK
*Correspondence: pisconti@liverpool.ac.uk
https://doi.org/10.1016/j.stemcr.2018.01.007

SUMMARY

Satellite cells are adult muscle stem cells residing in a specialized niche that regulates their homeostasis. How niche-generated signals integrate to regulate gene expression in satellite cell-derived myoblasts is poorly understood. We undertook an unbiased approach to study the effect of the satellite cell niche on satellite cell-derived myoblast transcriptional regulation and identified the tumor suppressor p53 as a key player in the regulation of myoblast quiescence. After activation and proliferation, a subpopulation of myoblasts cultured in the presence of the niche upregulates p53 and fails to differentiate. When satellite cell self-renewal is modeled *ex vivo* in a reserve cell assay, myoblasts treated with Nutlin-3, which increases p53 levels in the cell, fail to differentiate and instead become quiescent. Since both these Nutlin-3 effects are rescued by small interfering RNA-mediated p53 knockdown, we conclude that a tight control of p53 levels in myoblasts regulates the balance between differentiation and return to quiescence.

INTRODUCTION

Satellite cells (SCs) are quiescent muscle stem cells residing in a specialized anatomical niche located between the plasma membrane of the muscle fiber and the surrounding basal lamina (Mashinchian et al., 2017). In response to muscle damage, SCs become activated and re-enter the cell cycle. After one or more rounds of proliferation, the vast majority of SC-derived muscle progenitors (called myoblasts) exit the cell cycle and enter a terminal G0 phase that leads to differentiation, followed by fusion to existing damaged muscle fibers to repair them or one-another to generate new muscle fibers. During this process, a small portion of myoblasts do not differentiate and rather enters a reversible G0 phase of the cell cycle, effectively replenishing the pool of quiescent SCs (Olguín and Pisconti, 2012). The mechanisms that regulate these fate decisions have been investigated, and at least two models have been proposed. In the first model, activated SCs divide asymmetrically upon activation giving rise to a daughter cell that self-renews and another daughter cell that becomes a myoblast and gives rise to a myogenic progeny (Dumont et al., 2015; Kuang et al., 2007; Troy et al., 2012). In a second model, proliferating myoblasts are induced to overexpress *Pax7*, which in turn inhibits myogenin expression and promotes entry into a mitotically quiescent state (Olguín and Olwin, 2004; Wen et al., 2012). In both cases, a key role appears to be played by the extracellular environment, called the SC niche (Mashinchian et al., 2017).

The SC niche is the complex set of molecules surrounding the SC in its anatomical location and the receptors that are expressed on its surface. Several of these molecules play important roles in driving SC fate (Mashinchian et al., 2017). However, it is also well established that when SCs are completely stripped of their native niche and cultured on a proteinaceous substrate, usually collagen, laminin, or gelatin, they retain the capacity to recapitulate the fate choices normally made in the presence of the niche, including proliferation, differentiation, fusion, and generation of a population of quiescent cells resembling self-renewed SCs (Olguín and Olwin, 2004). These observations raise two questions: (1) Are SCs primed to follow the myogenic program regardless of the presence of the niche? (2) Is the transcriptional program that drives these fate decisions in SCs the same in the presence of the niche and its absence?

Here we attempt to answer these questions by investigating gene expression in SC-derived myoblasts cultured under two different conditions: in the presence of their native niche (on isolated myofibers) or in its absence (on gelatin-coated dishes). We show how myoblast gene expression is affected by the presence of the niche and identify the p53 gene network as a key regulator of myoblast fate in the presence of the SC niche. Lastly, we show that a sustained increase in p53 during myoblast cell-cycle exit inhibits myoblast differentiation while promoting quiescence.

This is an open access article under the CC BY license (http://creativecommons.org/licenses/by/4.0/).

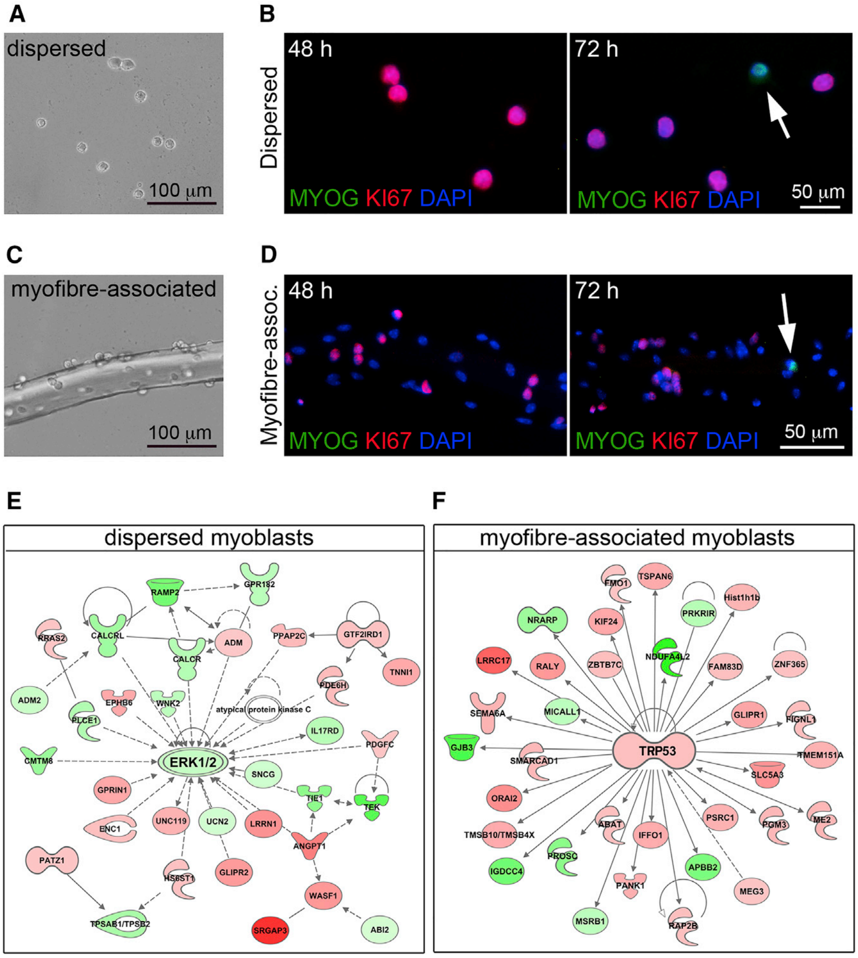

Figure 1. Cell-Cycle Exit and Terminal Differentiation Are Induced in Both Myofiber-Associated and Dispersed Myoblasts between 48 and 72 hr after Isolation

(A and B) Dispersed myoblasts cultured on gelatin-coated plates show a rounded morphology (A) and proliferate extensively in the first 2–3 days as revealed by positive staining for the cell-cycle marker KI67+. No differentiating cells are detected at 48 hr after isolation (B). As early as 72 hr post-isolation occasionally MYOG+ cells are detected in dispersed cultures (B), arrow.

(C and D) For the first 2 days myofiber-associated myoblasts (C) proliferate as revealed by positive staining for KI67+ and absence of differentiating (MYOG+) cells (D). At 72 hr after isolation a few MYOG+ cells are occasionally detected (D), arrow.

(E and F) Genes differentially expressed between 48 and 72 hr in dispersed (E) and myofiber-associated (F) myoblasts were mapped to canonical gene networks using IPA, revealing that the top most enriched gene network in dispersed myoblasts is centered around *Erk1/2* downregulation (E), while the top most enriched network in myofiber-associated myoblasts is centered around *Trp53* upregulation (F). Genes labeled in green are downregulated, genes labeled in red are upregulated at 72 hr compared to 48 hr. The color intensity is proportional to the extent of up- or downregulation.

RESULTS

Dispersed and Myofiber-Associated Myoblasts Exit the Cell Cycle and Initiate Differentiation with Similar Timing

When SCs are isolated from the muscle tissue and cultured on gelatin-coated dishes, they extensively proliferate for the first 2–3 days in culture (Figures 1A, 1B, and S1). Proliferating SCs express *Pax7*, *Myf5*, and *MyoD1* and are often referred to as myoblasts. On the fourth day in culture, a few myotubes can be already observed (Figure S1). Indeed, myogenin-positive (MYOG+) cells are occasionally observed on the third day in culture (Figure 1B), suggesting that SC-derived myoblasts in dispersed cultures begin to exit the cell cycle and undergo terminal differentiation between 48 and 72 hr after isolation. Similarly, on day 3 in culture, MYOG+ cells are observed amongst myofiber-associated myoblasts (Figures 1C and 1D), which are cultured in the same medium as dispersed myoblasts. This suggests that the timing of myoblast cell-cycle exit and entry into terminal differentiation are comparable regardless of the presence of the niche. To test whether these comparable timings were driven by comparable transcriptional programs, we carried out a global gene expression analysis of SC-derived myoblasts cultured either in dispersed cultures or on explanted myofibers. We profiled gene expression in myoblasts from both cell culture types at 48 and 72 hr after isolation, when cell-cycle exit and commitment to terminal differentiation appear to occur under both culture conditions (Figures 1A–1D).

Myoblast Cell-Cycle Exit Is Associated with Different Transcriptional Signatures in the Presence or Absence of the SC Niche

We collected four biological replicates for each time point (48 and 72 hr) in each culture condition and analyzed gene expression by microarray technology. The extent of reproducibility across replicates was excellent (Figures S2A and S2B). By contrast, the myoblast transcriptome at 48 hr was remarkably different from the transcriptome at 72 hr under both culture conditions, as evidenced by the large

number of differentially expressed genes (at $q < 0.01$) detected between 48 and 72 hr under either culture conditions: 1,810 in dispersed myoblasts and 1,999 in myofiber-associated myoblasts. Interestingly, when we compared the 72 hr/48 hr fold changes between the two culture conditions, it appeared evident that gene expression changes between 48 and 72 hr were different in the two culture conditions (Figure S2C). To gain insight into the molecular mechanisms that were associated with these dramatic changes in the transcriptional signature of myoblasts between 48 and 72 hr in either dispersed or myofiber-associated cultures, we mapped the differentially expressed genes to known gene networks using Ingenuity Pathway Analysis (IPA). The top most enriched network to which differentially expressed genes from dispersed myoblasts mapped, was centered around a decrease in the intracellular kinases *Erk1* and *Erk2* (Figure 1E). In contrast, the top most enriched network to which differentially expressed genes from myofiber-associated myoblasts mapped, was centered around an increase in the tumor suppressor *Trp53* (p53) (Figure 1F). ERK1/2 are key promoters of myoblast proliferation (Jones et al., 2001) and, similarly, an increase in p53 levels is expected to lead to cell-cycle arrest (Levine, 1997). Thus, these results are consistent with our initial hypothesis that between 48 and 72 hr both dispersed and myofiber-associated myoblasts prepare to exit the cell cycle, though via different molecular mechanisms.

The Signaling Pathways that Regulate Cell-Cycle Exit in the Presence or Absence of the Niche Are Different

To further our understanding of the molecular mechanisms regulating SC gene expression in the presence and absence of the SC niche, we analyzed the canonical signaling pathways that were enriched between 48 and 72 hr in dispersed and myofiber-associated myoblasts using IPA, which assigns an activation score to a signaling pathway based on the direction and extent of change of the differentially expressed genes mapped to that signaling pathway. The first observation was that the vast majority of canonical signaling pathways moved in different directions between 48 and 72 hr in myofiber-associated versus dispersed myoblasts (Figure 2A).

When we then analyzed in more detail the IPA category *Cell Cycle*, the most striking feature was a strong activation of *Cdk5 signaling* (Figure 2B) observed in both culture conditions, indicating cell-cycle arrest and preparation to differentiate (Lazaro et al., 1997; Sarker and Lee, 2004). In myofiber-associated myoblasts this was accompanied by an even stronger activation of *ATM signaling*, which is upstream of p53 and also induces cell-cycle arrest (Figure 2B). Consistently, signaling pathways that promote proliferation, such as *Ceramide signaling* (Gangoiti et al., 2012), *G2/M transition signaling*, *Cyclin signaling*, and *Aryl hydrocarbon receptor signaling* (Barouki et al., 2007; Yin et al., 2016), were inactivated in myofiber-associated myoblasts (Figure 2B). Similarly, dispersed myoblasts showed a marked inactivation of *Integrin signaling*, also pointing toward inhibition of proliferation (Figure 2B). Thus, canonical signaling pathways related to cell-cycle regulation are activated or deactivated in both culture conditions in a manner that supports preparation to exit the cell cycle. The only exception to this was the *G1/S checkpoint regulation* signaling pathway, which was activated in myofiber-associated but not dispersed myoblasts (Figure 2B). Similarly, the IPA category *Cell Growth and Proliferation* showed a series of pro-proliferative signaling pathways (ILK, HGF, PDGF, CREB, P70S6K, and CDC42 signaling pathways) that were activated in myofiber-associated myoblasts but deactivated in dispersed myoblasts (Figure 2C). Since proliferation and cell-cycle exit are mutually exclusive, the fact that both pro-proliferation and pro-cell-cycle exit pathways are activated in myofiber-associated myoblasts, supports previous findings that myofiber-associated myoblasts are heterogeneous (Ono et al., 2010).

Lastly, we analyzed the IPA category *Growth Factor Signaling* (Figure 2D). Amongst the signaling pathways that were differentially activated between dispersed and myofiber-associated myoblasts we found activation of pro-proliferation signaling (*HGF*) and self-renewal signaling (GDNF signaling, which requires syndecan-3 (SDC3), and therefore is expected to promote self-renewal, Bespalov et al., 2011; Pisconti et al., 2010) in myofiber-associated but not in dispersed myoblasts.

Comparative Analysis of Gene Expression Suggests a Role for the Niche in Driving Self-Renewal

To understand the differences in myoblast gene expression changes due to culture conditions we compared ratios of 72 hr/48 hr mRNA levels within each culture condition with each other (Figure S2C) and generated a fold-change ratio using the formula:

$$\text{Comparative ratio(CR)} = \frac{(\text{mRNA at 72 hr/mRNA at 48 hr})\text{dispersed}}{(\text{mRNA at 72 hr/mRNA at 48 hr})\text{myofiber-associated}}$$

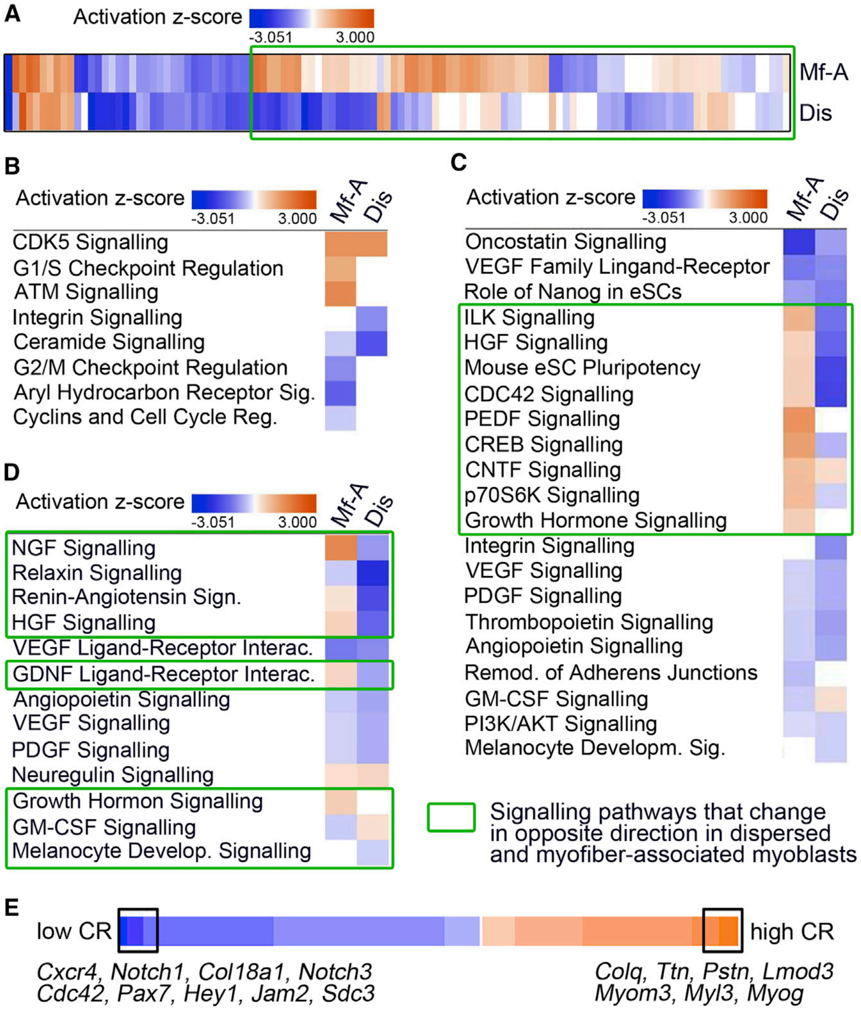

Figure 2. Canonical Signaling Pathways Are Differentially Activated in Dispersed and Myofiber-Associated Myoblasts

(A) Genes differentially expressed between 48 and 72 hr post-isolation in dispersed (Dis) and myofiber-associated (Mf-A) myoblasts were functionally mapped to all canonical signaling pathways listed by IPA. For each signaling pathway, an enrichment p value and a Z score of activation were calculated and the pathways with enrichment p value < 0.01 (−log[p value] > 1.3) are plotted as heatmaps, were orange represents a positive Z score (= activation), blue is a negative Z score (deactivation), and white is $Z = 0$. Canonical signaling pathways that were not enriched enough to show a Z score were excluded. Canonical signaling pathways that change in opposite direction in dispersed and myofiber-associated myoblasts are highlighted by a green box.

(B–D) Heatmaps obtained as in (A) for canonical signaling pathways mapping to the IPA categories: Cell Cycle (B), Cell Growth and Proliferation (C), and Growth Factor Signaling (D).

(E) Heatmap distribution of the comparative ratio (CR) = ([72 hr/48 hr]$_{dispersed}$/[72 hr/48 hr]$_{myofiber\text{-}associated}$) for gene families that are involved in myogenesis (manual annotation, see Experimental Procedures section).

Genes that score high CR values (>1) are genes that increase in dispersed but not in myofiber-associated myoblasts between 48 and 72 hr, while genes that score low CR values (<1) are genes that increase in myofiber-associated but not in dispersed myoblasts between 48 and 72 hr. We identified 2,583 genes with CR > 1 and 3,120 genes with CR < 1 at 1% false discovery rate (FDR), suggesting very different time-dependent responses between the two culture conditions. After filtering the resulting list of genes to include only genes with a q value < 0.01, we then functionally analyzed these genes by mapping them to the gene ontology (GO) category *Biological Process* using the online tool DAVID (Tables S1 and S2). Table S1 shows the GO annotation of genes that had CR > 2, while Table S2 shows the GO annotation of genes that had CR < 0.5.

The most significant GO terms to which genes that increase in dispersed but not in myofiber-associated myoblasts (CR > 2) map are associated with cell movement (*Taxis*) and muscle differentiation (*Striated Muscle Differentiation*) (Table S1). Interestingly, the term *Negative regulation of transport* also included genes that promote muscle differentiation, such as *Il-6* and *Nos1* (De Palma et al., 2010; Hoene et al., 2013). In contrast, the genes that increase between 48 and 72 hr in myofiber-associated myoblasts but not in dispersed myoblasts (CR < 0.05) map to *Cell Adhesion, Differentiation*, and *Cell Fate Commitment* (Table S2). The latter appears to be related mostly to maintenance of stemness as it contains genes that are associated with stemness in SCs (*Notch1, Notch3, Pax7,* and *Sox8*; Bjornson et al., 2012; Gopinath et al., 2014; Olguín and Olwin, 2004; Olguín and Pisconti, 2012; Pisconti et al., 2010; Schmidt et al., 2003).

Lastly, we organized the differentially expressed genes according to their CR and filtered the gene list through a manually curated list of gene families that have been shown to play a role in myogenesis (see Experimental Procedures for details). This analysis showed that genes associated with muscle differentiation were increased

in dispersed but not in myofiber-associated myoblasts (Table S3; Figure 2E: *Myog, Myl3, Myom3, Lmod3, Postn, Ttn,* and *Colq*). By contrast, genes associated with self-renewal were increased in myofiber-associated but not dispersed myoblasts (Table S3; Figure 2E: *Cxcr4, Col18a1, Notch1, Notch3, Cdc42, Pax7, Hey1, Jam2,* and *Sdc3*).

To summarize the data shown so far: *Erk1/2* downregulation in dispersed myoblasts is mostly associated with activation of signaling pathways that lead to cell-cycle arrest and the onset of differentiation. In contrast, *Trp53* upregulation in myofiber-associated myoblasts is associated with a more heterogeneous transcriptomic signature in which signaling leading to cell-cycle arrest, differentiation, self-renewal, and proliferation co-exist. Since the role of p53 in myogenesis is complex and not well understood, we decided to focus on understanding how an increase in p53 levels might affect myoblast cell fate.

An Asymmetric Increase in p53 Protein Levels Is Incompatible with Myogenic Differentiation in Myofiber-Associated Myoblasts

The expression of p53 is rapidly and transiently upregulated in the first hours of C2C12 myoblast differentiation induced by serum deprivation (Halevy, 1993), and is important for myogenic differentiation, as expression of a p53 dominant-negative mutant leads to impaired differentiation (Soddu et al., 1996), but is not involved in differentiation-induced apoptosis (Cerone et al., 2000). Thus, it could be speculated that the upregulation of p53 that we observe in myofiber-associated myoblasts drives differentiation. However, it has been recently shown that a sustained increase in p53 levels, such as that due to genotoxic stress, or treatment with the MDM2 inhibitor Nutlin-3, impairs myogenic differentiation, possibly via direct inhibition of myogenin expression (Walsh et al., 2015; Yang et al., 2015). Moreover, p53 mediates hypoxia-induced inhibition of myoblast differentiation (Wang et al., 2015).

To investigate the role of the increase in p53 gene expression detected by microarray in myofiber-associated myoblasts, we first tested whether such an increase in mRNA levels was accompanied by an increase in p53 protein levels and whether it was associated with differentiation. Indeed, p53 levels appeared to increase in myofiber-associated myoblasts, identified by the general myoblast marker SDC3 (Cornelison et al., 2001) between 48 and 72 hr in culture, as measured by an increase in the proportion of p53+/SDC3+ myoblasts (Figures 3A and 3B). Intriguingly, we occasionally observed an asymmetric distribution of p53 in myoblast doublets (Figure 3C), which was more prominent at 72 hr (Figure 3D), and was accompanied by an almost perfect inverse correlation with myogenin (MYOG) expression at 96 hr (Figure 3E). When we measured p53 protein levels across the same 3-day time course in dispersed myoblasts, we found only a small and not significant increase in p53 protein levels over time (Figure S3). Moreover, immunofluorescence analysis of primary dispersed myoblasts showed that p53 protein was still present in MYOG+ dispersed myoblasts, although often at lower levels than in MYOG– cells (Figure 3F). These results suggest that, when myoblasts are cultured in the presence of their native niche, p53 protein levels increase over time dramatically and selectively in some cells, and in these cells myogenic differentiation is inhibited (Figure 3E). When the niche is absent, such a dramatic and sustained increase occurs in fewer cells, while the majority of cells maintain lower levels of p53, which are permissive of myogenic differentiation (Figure 3F).

If it is true that the presence of the SC niche promotes p53 upregulation in a subpopulation of myofiber-associated myoblasts and that this in turn inhibits myogenic differentiation in these cells, then decreasing p53 in myofiber-associated myoblasts via RNAi should promote myoblast differentiation in myofiber cultures. To test this hypothesis, we transfected myofiber cultures with either p53 small interfering RNA (siRNA) or with control (scrambled) siRNA at 48 hr post-isolation. Transfection of C2C12 myoblasts was used to validate p53 knockdown efficiency (Figure S4). Two days after transfection, we fixed and immunostained the myofibers to detect and quantify MYOG+ myoblasts (Figure 4A). As predicted, the percentage of MYOG+/SDC3+ cells was increased in p53 siRNA-transfected cultures compared with control siRNA-transfected cultures, although the difference did not reach statistical significance (Figure 4B). The fact that the difference was small, could be due to the high propensity of differentiated myofiber-associated myoblasts to immediately fuse with the underlying fiber (Kuang et al., 2007), which removes them from the equation when MYOG+/SDC3+ cells are scored as a measure of differentiation. Indeed, the number of myonuclei per unit length was significantly increased (Figure 4C), while the frequency of myoblasts (SDC3+ cells) per unit length was decreased (Figure 4D) upon p53 knockdown, supporting the idea that p53 inhibits differentiation in a subpopulation of myofiber-associated myoblasts.

The next question was: what is the fate of these myoblasts that show high p53 protein levels and fail to differentiate? Since high levels of p53 are incompatible with proliferation, we hypothesized that a sustained increase in p53 levels in myoblasts would lead to apoptosis, senescence, or quiescence.

An Increase in p53 Protein Levels Promotes Myoblast Quiescence

Nutlin-3, a small molecule compound that blocks the interaction between p53 and the E3 ligase MDM2, is often used to increase the levels of p53 protein in the cell (Vassilev,

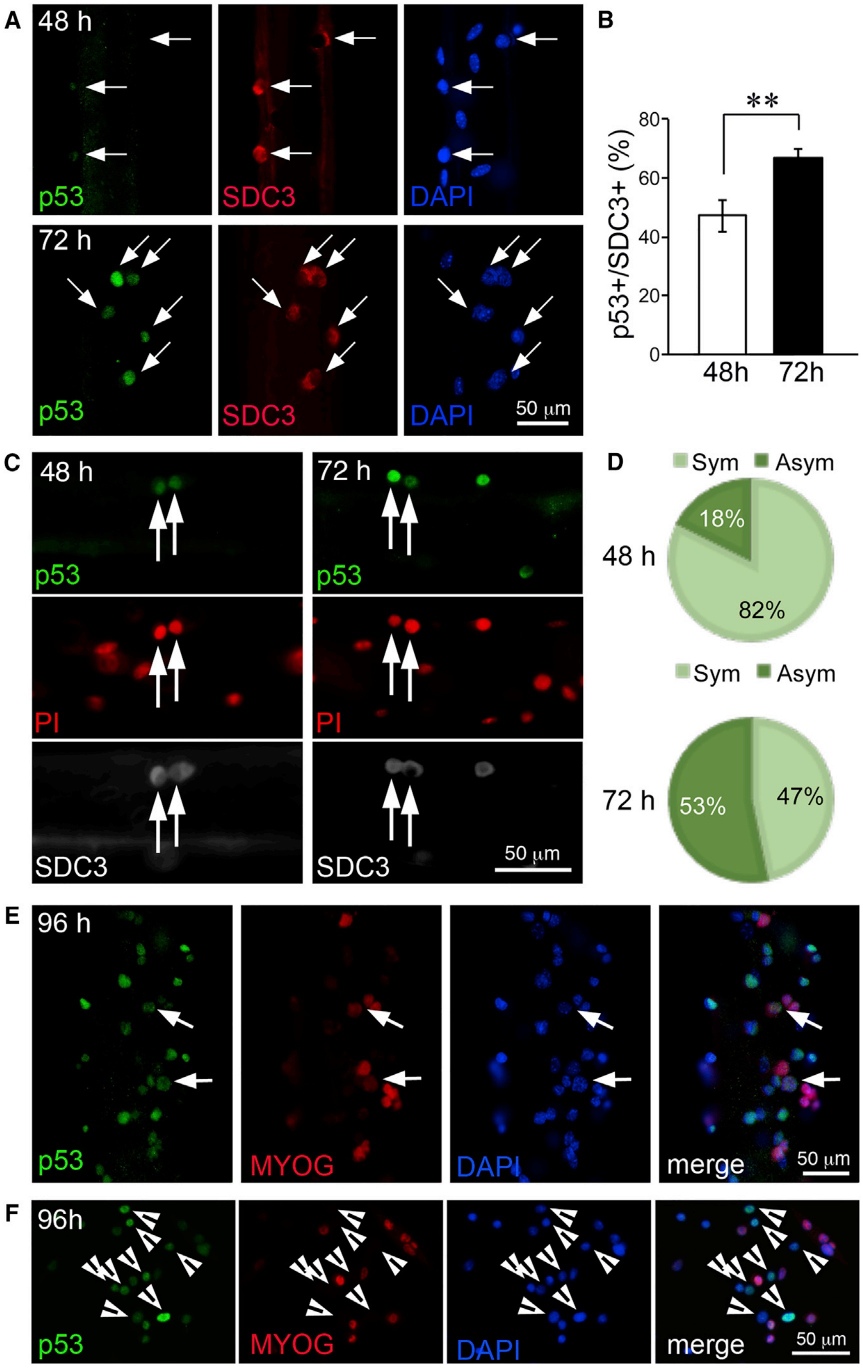

Figure 3. p53 Increases over Time in a Subset of Myofiber-Associated Myoblasts and Is Asymmetrically Distributed

(A) Individual myofibers were isolated and cultured in suspension for 48 and 72 hr prior to fixation and immunostaining to detect p53 (green), the myoblast marker syndecan-3 (SDC3, red), and DNA (DAPI, blue). Arrows indicate SDC3+ SCs.

(B) Quantification of (A) where at least 15 myofibers across 3 independent experiments had been scored, and the percentage of p53+ cells over the total number of SDC3+ cells plotted as average ±SEM. $^{**}p < 0.01$.

(C) Individual myofibers were isolated and cultured as in (A) then immunostained to detect p53 (green), SDC3 (white), and DNA (PI, red). Arrows indicate doublets of dividing cells where p53 is distributed either symmetrically (48 hr, left panels) or asymmetrically (72 hr, right panels).

(D) Quantification of (C) where at least 15 fibers across 3 independent experiments had been scored.

(E and F) p53+/MYOG− myoblasts are very abundant in myofiber cultures (E) but less in dispersed cultures (F). Individual myofibers or primary myoblasts were isolated and cultured for 96 hr, then fixed and immunostained to detect p53 (green), myogenin (MYOG, red), and DNA (DAPI, blue). Arrows in (E) indicate p53+/MYOG+ cells, while all the other cells are either p53+/MYOG− or p53-/MYOG+. Arrowheads in (F) indicate p53+/MYOG− cells. All the other cells are p53+/MYOG+.

2005). Nutlin-3 treatment of C2C12 myoblasts leads to a generalized increase in p53 protein levels and both proliferation and differentiation inhibition (Walsh et al., 2015), thus supporting our hypothesis that a sustained increase in p53 levels in primary myoblasts might lead to apoptosis, senescence, or quiescence, as well as differentiation inhibition. To test this hypothesis we treated primary dispersed myoblasts with increasing concentrations of Nutlin-3 and verified that indeed Nutlin-3 inhibits proliferation (Figure S5A). However, Nutlin-3 treatment does not seem to increase apoptosis in primary myoblasts (Figure S5B), which is consistent with the absence of a role for p53 in serum deprivation-induced myoblast apoptosis (Cerone et al., 2000). Since Nutlin-3 does not promote differentiation, in fact it inhibits differentiation (Figure S6), the lack of apoptosis suggests that Nutlin-3-mediated inhibition of

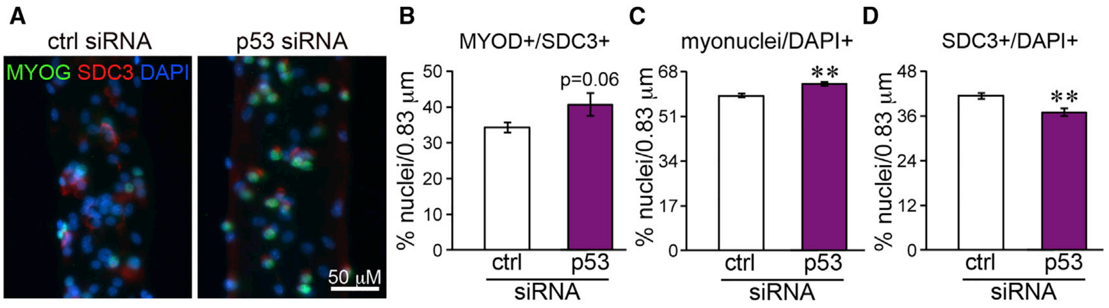

Figure 4. p53 Knockdown Promotes Myoblast Differentiation in Myofiber Cultures
(A) Individual myofibers were isolated and cultured in suspension for 48 hr prior to transfection with either a specific p53 siRNA or a scrambled (ctrl) siRNA and 4 hr later fixed and immunostained to detect myogenin (MYOG, green), syndecan-3 (SDC3, red), and DNA (DAPI, blue).
(B) Differentiated myoblasts were scored as percentage of MyoG+/SDC3+ myoblasts over total nuclei per unit length across at least eight myofibers/experiment in three independent experiments (N > 24), and plotted as average ± SEM.
(C) The number of myonuclei per unit length was measured as total number of DAPI+ nuclei minus the number of nuclei contained in SDC3+ cells (since SDC3 marks SCs and myoblasts at all stages in myogenesis) per unit length across at least eight myofibers/experiment in three independent experiments (N > 24), and plotted as average ± SEM.
(D) The percentage of myoblasts was calculated as percentage of nuclei contained in SDC3+ cells over the total number of DAPI+ nuclei per unit length across at least eight myofibers/experiment in three independent experiments (N > 24), and plotted as average ± SEM.
**p < 0.01 when comparing the indicated population scored in control siRNA-transfected cultures with the same population scored in p53 siRNA-transfected cultures.

proliferation is due to induction of either quiescence or senescence.

To discriminate between quiescence and senescence we performed a reserve cell assay. Cultures of both primary dispersed myoblasts and C2C12 myoblasts provide a validated *ex vivo* model of SC self-renewal: upon serum lowering, the vast majority of myoblasts exit the cell cycle with a portion of them downregulating PAX7 and undergoing terminal differentiation, and another portion maintaining high levels of PAX7 and becoming “reserve cells” (Yoshida et al., 1998). In reserve cell assays (Figure 5A), Nutlin-3 treatment of both primary and C2C12 myoblast cultures dramatically increased the percentage of putative reserve cells (PAX7+/KI67–) generated by serum lowering (Figures 5B, 5C, 5E, and 5F). This was accompanied by a decrease in the percentage of proliferating myoblasts (PAX7+/KI67+) in C2C12 cultures (Figures 5E and 5G), although no significant changes in PAX7+/KI67+ cells were detected in primary myoblast cultures (Figures 5B and 5D).

In addition to being mitotically quiescent, reserve cells also show decreased levels of MYOD (Yoshida et al., 1998), thus “true” reserve cells are PAX7+/KI67–/MYOD– (Yue et al., 2016). Although we could effectively label C2C12 myoblasts to simultaneously detect PAX7, KI67, and MYOD, this could not be achieved with primary myoblasts (triple PAX7/KI67/MYOD staining of primary myoblasts showed cytoplasmic positivity for MYOD and/or KI67, even at very low antibody concentration, which is not observed when either KI67 or MYOD antibodies are used in a double staining of C2C12 cells with PAX7). Thus, we were forced to label primary myoblasts separately for PAX7 and KI67 (as discussed above) and PAX7 and MYOD. Nutlin-3 treatment dramatically reduced MYOD protein levels and led to an increase in the frequency of true reserve cells (PAX7+/KI67–/MYOD–) in C2C12 myoblast cultures (Figures 5K–5L), which was accompanied by a decrease in proliferating myoblasts (PAX7+/MYOD+/KI67+; Figures 5K and 5M). Similarly, Nutlin-3 treatment produced a 20-fold increase in the numbers of uncommitted myoblasts (PAX7+/MYOD– cells; Figures 5H and 5I), but only a 6-fold increase in the percentage of committed myoblasts (PAX7+/MYOD+; Figures 5H and 5J) in primary myoblast cultures. Although in the absence of a triple PAX7/MYOD/KI67 staining it is not possible to establish whether Nutlin-3 promotes true reserve cell generation in primary myoblast cultures as it does in C2C12 cultures, the observations that: (1) the increase in uncommitted myoblasts produced by Nutlin-3 is over 3 times greater than the increase in committed myoblasts (20- versus 6-fold), and (2) PAX7+/KI67+ myoblasts do not increase in response to Nutlin-3 in primary cultures (Figure 5D), while PAX7+/KI67– cells increase 7-fold (Figure 5C), suggest that the percentage of true reserve cells (PAX7+/KI67–/MYOD–) is likely to significantly increase in primary myoblast cultures as it does in C2C12 cultures in response to Nutlin-3 treatment.

In contrast to differentiated and senescent cells, which are in an irreversible G0 phase, reserve cells are quiescent,

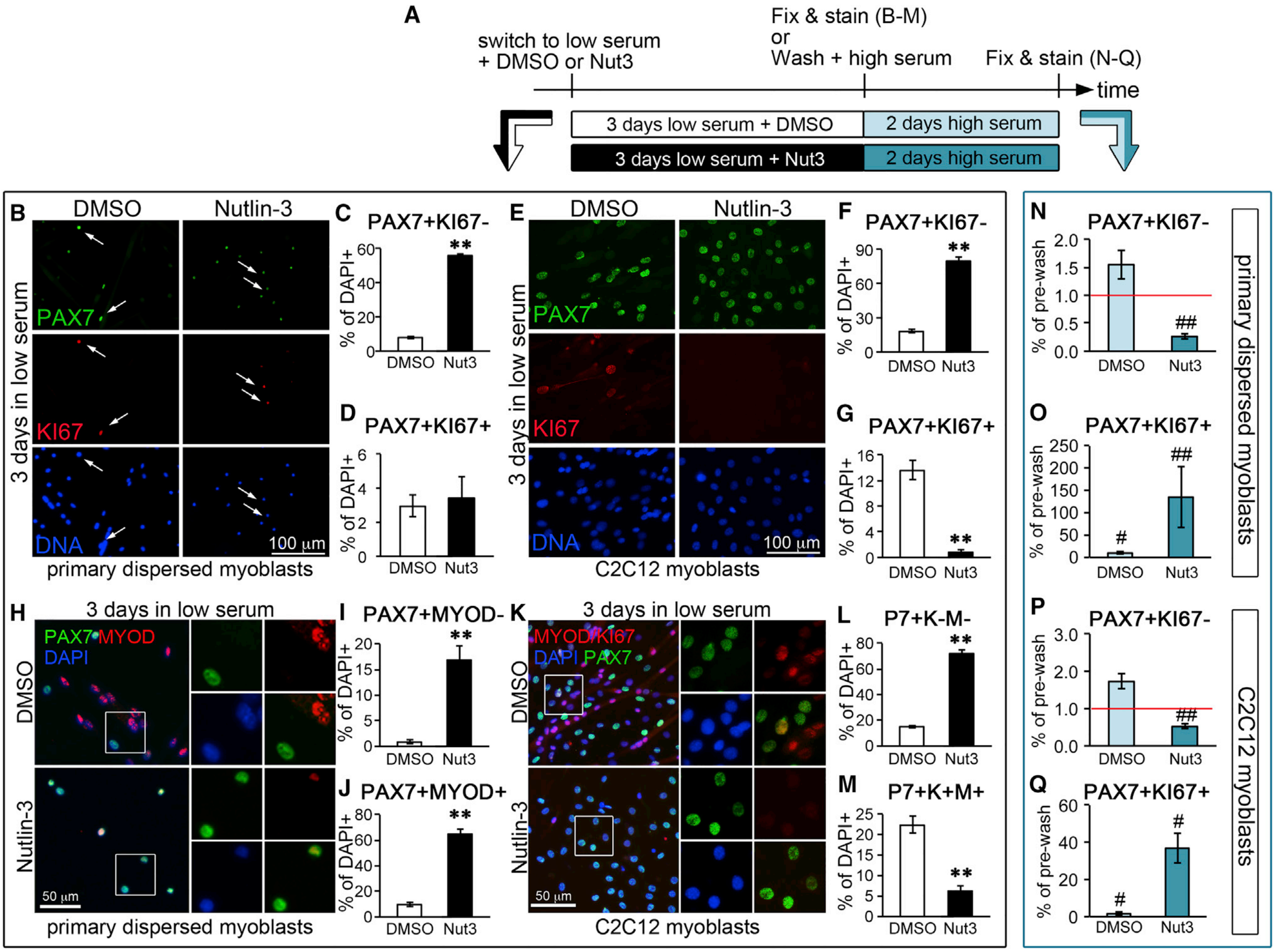

Figure 5. Nutlin-3 Promotes Reserve Cell Generation in Serum-Deprived Myoblasts

(A) Schematic representation of the reserve cell assay experimental design. Primary and C2C12 myoblast cultures were switched to low serum medium to induce cell-cycle exit in the presence of either 20 μM Nutlin-3 (Nut3) or vehicle (DMSO). To distinguish between quiescence and senescence, myoblast cultures that had been maintained in low serum supplemented with either DMSO or Nutlin-3 for 3 days were re-exposed to high serum for 2 days. The experiment was repeated 3 times independently and each time 10–15 technical replicates were scored.

(B–D) Primary myoblast cultures were treated as in (A) and, after 3 days in low serum, fixed, immunostained to detect PAX7 (green), KI67 (red), and DNA (DAPI, blue), and scored for the percentages of PAX7+/KI67− (C) and PAX7+/KI67+ (D) cells. In (B), one representative image for each treatment is shown. In (C) and (D) quantitative analyses of the indicated cell subpopulations across all three independent experiments were plotted as average ± SEM. The arrows in (B) indicate PAX7+/KI67+ cells.

(E–G) C2C12 myoblasts cultures were treated as in (A) and, after 3 days in low serum, fixed, and immunostained as in (B). In (E) one representative image for each treatment is shown. In (F) and (G) quantitative analyses of the indicated cell subpopulations across all three independent experiments were plotted as average ± SEM.

(H–M) Primary (H and J) and C2C12 (K–M) myoblasts that had been maintained in low serum supplemented with either DMSO or Nutlin-3 for 3 days were fixed and immunostained to detect PAX7 (green), KI67, and/or MYOD (red) and DNA (DAPI, blue). In (H) and (K) one representative image for each treatment is shown. Insets are enlarged on the side of the main image to show mutual exclusion or co-localization of PAX7 and MYOD (H) or PAX7 and MYOD+ KI67 (K). In (I), (J), (L), and (M) quantitative analyses of the indicated cell subpopulations across all three independent experiments were plotted as average ± SEM.

(N–Q) In order to quantify true reserve cells, after 3 days in low serum, primary (N and O) and C2C12 (P and Q) myoblast cultures were re-exposed to high serum as depicted in (A) for 2 days, then fixed and immunostained to detect PAX7, KI67, and DNA. The percentages of PAX7+/KI67− (N and P) and PAX7+/KI67+ (O and Q) were calculated and plotted as fold change of the same population in cultures that had been maintained in low serum and then re-exposed to high serum (we will call it “post-wash” for simplicity) versus the same population in cultures maintained for only 3 days in low serum (indicated as “pre-wash”). The average of the post-wash/pre-wash fold change for each

(legend continued on next page)

in a reversible G0 phase, and therefore can be induced to re-enter the cell cycle by adding serum to the culture medium (Yoshida et al., 1998). To discriminate whether the putative reserve cells generated in myoblast cultures in response to Nutlin-3 treatment were truly quiescent or senescent, we exposed again to high serum the cultures that had been maintained for 3 days in low serum supplemented with either Nutlin-3 or DMSO, and 2 days later we measured the percentages of cells that had re-entered the cell cycle (Figure 5A). In both C2C12 and primary myoblast cultures washed and re-exposed to high serum, the percentage of PAX7+/KI67+ cells increased more dramatically if the cultures had been previously treated with Nutlin-3 compared with vehicle (Figures 5O and 5Q). Interestingly, the percentage of PAX7+/KI67– cells did not significantly change upon serum re-exposure in cultures that had been previously treated with vehicle, but significantly decreased in cell cultures that had been previously treated with Nutlin-3 (Figures 5N and 5P). These results indicate that the percentage of *true* reserve cells is normally very low in our reserve cell assay, regardless of the origin of the myoblasts used (primary or C2C12). However, this percentage of *true* reserve cells is dramatically increased by Nutlin-3 treatment.

An Increase in p53 Levels Is Directly Responsible for the Increased Reserve Cell Generation in Response to Nutlin-3

To test whether Nutlin-3 promoted reserve cell generation and inhibited differentiation directly via p53, we transfected C2C12 myoblasts with either a control siRNA or with an anti-p53 siRNA, and 3 hr later we replaced the transfection medium with low serum medium (to induce cell-cycle exit followed by myotube and reserve cell generation) supplemented with either Nutlin-3 or vehicle. Although p53 siRNA transfection did not appear to alter the differentiation capacity of C2C12 myoblasts treated with vehicle, it partly rescued the differentiation levels of cells treated with Nutlin-3 (Figure 6A). More importantly, the percentage of PAX7+/KI67– reserve cells that was increased by Nutlin-3 in cultures transfected with control siRNA, was then rescued by transfection with a specific anti-p53 siRNA (Figures 6B and 6C), further indicating a direct role for p53 in regulating the balance between differentiation and reserve cell generation. Interestingly, the decrease in the percentage of PAX7+/KI67– reserve cells caused by p53 knockdown in Nutiln-3-treated cultures was not due to a rescue of the number of proliferating (PAX7+/KI67+) myoblasts (Figure 6B), but to a decrease in differentiation. This suggests that Nutlin-3 promotes reserve cell generation via p53 by affecting directly the cell fate decision to either differentiate or become quiescent, rather than the earlier decision to either divide or exit the cell cycle.

DISCUSSION

In this study, we show that primary myoblasts cultured *ex vivo* carry out different transcriptional programs to regulate their cell fate transitions according to whether they are cultured in the presence or absence of their native niche. In the absence of the niche, the vast majority of myoblasts activate a transcriptional program dominated by *Erk1/2* downregulation and differentiate. In the presence of the niche, the transcriptional program is dominated by an upregulation of *Trp53*. Validation experiments revealed that a sustained p53 increase in myofiber-associated myoblasts is restricted to a subpopulation of cells that fail to differentiate. These myoblasts showing high levels of p53 are more rare in the absence of the niche, which is likely why p53 upregulation is not significantly detected in the transcriptomic analysis of the dispersed myoblasts. Thus, our data suggest that the presence of the niche leads to a greater percentage of myoblasts showing a sustained increase in p53 levels and associated fail to differentiate. We then used gain- and loss-of-function experiments in a culture context that promotes cell-cycle exit to determine the fate of these high p53 myoblasts that fail to differentiate, and showed that for the most part these myoblasts become quiescent. Our data therefore point toward p53 signaling as a regulator of the balance between differentiation and self-renewal: although a transient increase in p53 levels occurs in the early hours that precede differentiation and is likely necessary for cell-cycle exit, p53 must quickly return to basal levels upon cell-cycle exit in order for differentiation to occur (Figure 7). However, in some cells, p53 levels further increase and remain high over time, leading to differentiation inhibition and promotion of self-renewal (Figure 7). The latter process appears to be promoted when myogenesis occurs in the presence of the SC niche.

subpopulation across 3 independent biological replicates (each one scored for 10–15 technical replicates) was calculated and plotted ± SEM.
In (C) and (D), (F) and (G), (I) and (J), and (L) and (M): $^{**}p < 0.01$, where p is the p value of the average percentage of each cell subpopulation in Nut-3-treated cultures versus DMSO-treated cultures. In (N)–(Q), $^{\#}p < 0.05$ and $^{\#\#}p < 0.01$, where p is the p value of the fold change calculated as "subpopulation percentage in post-wash/subpopulation percentage in pre-wash" within each treatment (DMSO or Nut-3).

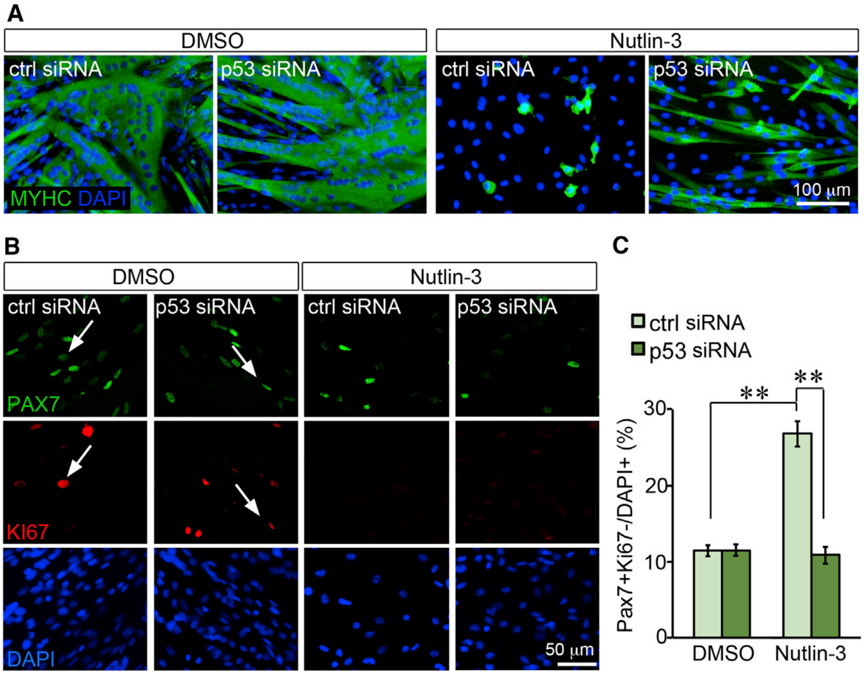

Figure 6. p53 Knockdown Rescues Reduced Differentiation and Increased Reserve Cell Generation in Myoblasts Treated with Nutlin-3

(A) Differentiation of C2C12 myoblasts is reduced by Nutlin-3 treatment and rescued by p53 knockdown. C2C12 myoblasts were transfected with either control or p53 siRNA, then maintained for 3 days in low serum supplemented with either DMSO or 20 μM Nutlin-3 prior to fixation and immunostaining to detect myosin heavy chain (MYHC, green) and DNA (DAPI, blue).

(B) Reserve cell (PAX7+/KI67−) generation is increased by Nutlin-3 treatment and rescued by p53 knockdown. C2C12 myoblasts were transfected and cultured as in (A) prior to fixation and immunostaining to detect PAX7 (green), KI67 (red), and DNA (DAPI, blue). Arrows point to PAX7+/KI67+ cells. All PAX7+ cells not labeled by an arrow are KI67−. All KI67+ cells that are not labeled by an arrow are PAX7−.

(C) Quantification of (B) where the average percentage of PAX7+/KI67− cells (over total DAPI+ cells) is calculated across 10–15 technical replicates for 3 independent experiments (N = 35) and plotted. Error bars are SEM. $^{**}p < 0.01$.

Our findings open up new questions regarding the molecular mechanisms that underlie p53-mediated regulation of the balance between differentiation and self-renewal. One possibility is that p53 regulates such balance via direct inhibition of myogenin expression (Yang et al., 2015) and consequently inhibition of differentiation, upregulation of PAX7, and activation of a program that resembles *in vivo* quiescence (Olguín and Olwin, 2004). By contrast, a direct upregulation of PAX7 by p53 appears unlikely (Yang et al., 2015). Interestingly, mice lacking the p53 target gene PW1 are born with fewer quiescent SCs (Nicolas et al., 2005). Since it is established that neonatal SCs are derived during development form MYOD+ myoblasts (Kanisicak et al., 2009), the finding that PW1 mutants lack quiescent SCs at birth further supports our view that p53 promotes generation of a population of quiescent SCs from proliferating myoblasts.

Although it had been previously shown that p53 inactivation via expression of a dominant-negative mutant decreases myoblast differentiation (Soddu et al., 1996) and that differentiation is decreased in primary *Trp53*$^{-/-}$ myoblasts (Porrello et al., 2000), when we decreased p53 levels in myoblasts by transfecting a specific anti-p53 siRNA we did not observe altered differentiation in the absence of Nutlin-3. This might be due to the fact that we lowered serum levels prior to an effective knock down of p53 (the medium was switched only 3 hr after transfection). Indeed, also reserve cell generation was not affected by p53 knockdown in cultures that had not been treated with Nutlin-3. Thus, it is possible that a transient peak of p53 expression still occurred in cultures that had not been treated with Nutlin-3 prior to effective p53 knockdown by siRNA, thus ensuring a normal distribution between differentiation and quiescence. Vice versa, in cultures treated with Nutlin-3, p53 levels either rapidly increased in response to Nutlin-3 above the normal transient peak and remained high for the entire duration of the experiment (control siRNA), or, in the absence of Nutlin-3, were prevented from increasing further than the normal transient peak by p53 siRNA, and in this second case the distribution between differentiation and quiescence was maintained at basal levels. Thus, our data suggest that, after cell-cycle exit, the levels of p53 in the cell determine whether the cell is going to enter a terminal G0 phase and differentiate (p53 levels returning low; Figure 7), or whether the cell is going to remain in a reversible G0 phase and effectively renew the pool of undifferentiated, quiescent SCs (p53 levels further increasing and remaining high; Figure 7).

Overall our study supports a model whereby myogenic differentiation is the preferred fate choice once SCs have been removed from their anatomical niche and cultured as myoblasts, whereas the presence of the niche provides signals that promote quiescence and self-renewal via multiple mechanisms, one of which leads to p53 upregulation. Further research is necessary in order to identify the molecular mechanisms through which the SC niche regulates

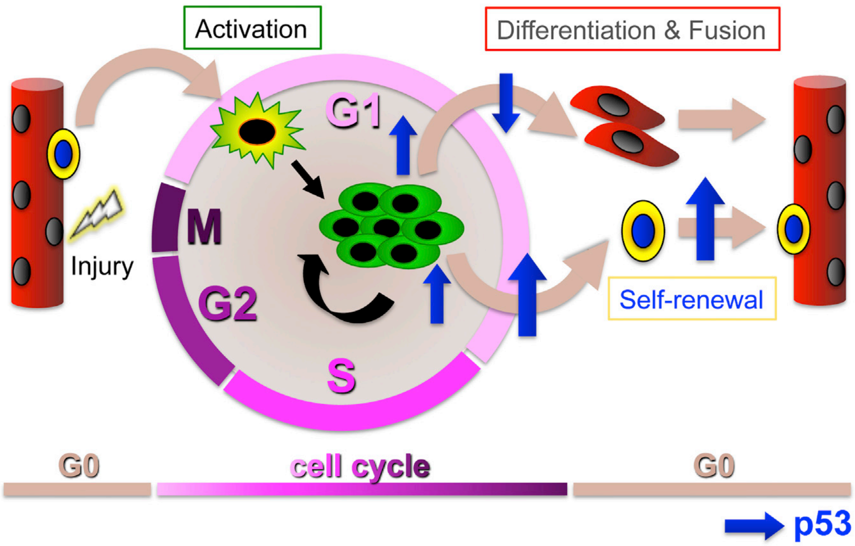

Figure 7. Schematic Representation of the Role of p53 in SC-Mediated Myogenesis

In uninjured muscle (red myofiber on the left), SCs (oval yellow cell on the myofiber) are quiescent, in a reversible G0 phase of the cell cycle. Upon injury (bolt) SCs become activated and re-enter the cell cycle in G1 (yellow/green, star-shaped cell). At each division cycle the two daughter cells (myoblasts, green rounded cells) choose between three main fates: divide again (and complete the cell cycle), or exit the cell cycle and then either differentiate (red elongated cells), or become quiescent (again oval yellow cell). Although a transient increase in p53 levels (upward blue arrow) allows cell-cycle exit, p53 must return to basal levels in order for differentiation to proceed (downward blue arrow). In contrast, a further and sustained increase in p53 levels (larger upward blue arrow) leads to differentiation inhibition and promotion of self-renewal (return to a quiescent, undifferentiated state).

p53 protein levels in myoblasts; however, our results strongly support the notion that p53 plays an important physiological role in muscle stem cell biology.

EXPERIMENTAL PROCEDURES

Mice

Male C57Bl/6J mice were purchased from Charles River and housed until used (at 12–13 weeks of age) in a pathogen-free facility at the University of Liverpool, in accordance with the Animals (Scientific Procedures) Act 1986 and the EU Directive 2010/63/EU, and after local ethical review and approval by the Animal Welfare and Ethical Review Body.

Myofiber Cultures

Single myofibers were isolated and cultured as described previously (Pisconti et al., 2016). Further details can be found in Supplemental Experimental Procedures.

Primary Dispersed Myoblast Cultures

Dispersed myoblasts were prepared and cultured as described previously (Pisconti et al., 2010). Further details can be found in Supplemental Experimental Procedures.

C2C12 Cell Cultures

C2C12 myoblasts (Yaffe and Saxel, 1977) were cultured as described previously (Arecco et al., 2016). Further details can be found in Supplemental Experimental Procedures.

Immunofluorescence

Myofibers, dispersed primary, and C2C12 myoblasts were fixed for either 20 min (myofibers) or 10 min (primary and C2C12 myoblasts) at room temperature with 4% paraformaldehyde prior to being processed for immunofluorescence as described previously (Pisconti et al., 2016). For a list of primary and secondary antibodies, the concentrations used and microscopy details, refer to Supplemental Experimental Procedures.

Isolation of Myoblasts from Myofiber Cultures

Forty-eight and 72 hr after isolation, myofibers were collected, washed twice with PBS, always collecting the supernatant which contained loosely attached myoblasts, then treated with 0.05% trypsin for 10 min at 37°C. The action of trypsin was then stopped by addition of 2 volumes of primary SC growth medium and the myofibers separated from stripped myoblasts by centrifugation (3 min at 100 × *g*): the supernatant was collected and combined with the two previous PBS washes. Myoblasts were then collected by centrifugation at 500 × *g*, the pellet washed once in PBS, and then immediately lysed in RLT buffer (RNeasy Kit, QIAGEN). This method yielded highly pure preparations of myofiber-associated myoblasts (Figures S6A–S6E) and high-quality RNA (Figures S6F and S6G), which did not contain contaminant RNA from the myofiber (see Supplemental Information for details).

RNA Extraction and Quality Control

RNA was extracted using the RNeasy Kit (QIAGEN) according to the manufacturer's instructions. The quality and concentration of the RNA was assessed using a Bioanalyzer (Agilent 2100, Agilent) or a Nanodrop (Thermo Fisher).

Microarrays

Sample labeling and microarray hybridizations were carried out according to Agilent's One-Colour Microarray-Based Gene Expression Analysis Protocol, v.6.6 (manual part number G4140-90040). Further details can be found in the Supplemental Experimental Procedures. Raw microarray data are available from

the GEO public depository under the accession number: GEO: GSE109052.

Microarray Analysis

Raw Agilent text files were read using the read. Agilent function in the marray R package (Yang, Paquet, and Dudoit, Exploratory analysis for two-color spotted microarray data. R package v.1.50.0. http://www.maths.usyd.edu.au/u/jeany/). Data were background corrected and loess normalized (using the normalizeWithinArrays function within the Limma package, followed by a quantile normalization using the normalizeBetweenArrays function) against their respective 48-hr data point to allow for a direct comparison between the two culture systems at both time points. Data were then summarized at the gene level by first averaging identical probes and then selecting a representative probe per gene name that showed the highest average intensity across the dataset. Genes with a median intensity level <5 across all arrays were removed. Differentially expressed genes were identified using the samr package (Tusher et al., 2001) for each of the three comparisons: (1) 72 versus 48 hr myofiber-associated myoblasts (Figure S2A), (2) 72 versus 48 hr dispersed myoblasts (Figure S2B), and (3) 72 hr/48 hr dispersed versus 72 hr/48 hr myofiber-associated myoblasts (Figure S2C). Genes significant at 1% FDR in each comparison were extracted and used for further analysis.

Manual Annotation of Gene Families Involved in Myogenesis

Gene families involved in myogenesis were identified manually via screening of literature published in PubMed between 01/01/1986 and 01/01/2016. A gene family was annotated as being involved in myogenesis when at least one member of the family was described as involved in myogenesis in at least one peer-reviewed publication. This list included but was not limited to: myogenesis regulatory factors, Pax genes, skeletal muscle sarcomeric proteins, various extracellular matrix components, calcitonin receptor, Wnt pathway, Notch pathway, receptor tyrosine kinase pathways (fibroblast growth factor, hepathocyte growth factor, epidermal growth factor, insulin growth factor, etc.), transforming growth factor β family pathways, interleukins, cytokines, chemokines, mitogen-activated protein kinases, p53 family, cyclins, cyclin-dependent kinases, etc. The comparative analysis filtered through this manually curated list is reported in full in Table S3.

Transfection

Myofiber and C2C12 cell cultures were transfected as described previously (Ghadiali et al., 2016). Further details can be found in Supplemental Experimental Procedures.

Western Blotting

Primary and C2C12 myoblasts were lysed in modified RIPA buffer (50 mM Tris-HCl [pH 7.5], 150 mM NaCl, 1% IGEPAL, 0.5% SDS, and 0.1% sodium deoxycholate) and western blotting performed as described previously (Arecco et al., 2016). A list of primary and secondary antibodies and concentrations used can be found in the Supplemental Experimental Procedures. Quantitative analysis of western blots was performed using the "Analyze Gel" function of ImageJ.

Statistical Analysis of Cell and Biochemistry Experiments

Experiments shown in Figures 3, 4, 5, and 6 were statistically analyzed using a t test as the data distributed normally. A p value < 0.05 was considered significant.

SUPPLEMENTAL INFORMATION

Supplemental Information includes Supplemental Experimental Procedures, seven figures, and three tables and can be found with this article online at https://doi.org/10.1016/j.stemcr.2018.01.007.

AUTHOR CONTRIBUTIONS

V.F. designed and performed experiments shown in Figures 1, 2, and S2. R.S.G. designed and performed experiments shown in Figures 4 and S3. P.A. performed the microarray analysis and the CR analysis. A.R. performed experiments shown in Figure 5. J.E.T. contributed to experimental design. A.P. contributed to experimental design and execution throughout the study and drafted the manuscript. All authors contributed to manuscript preparation by providing critical feedback.

ACKNOWLEDGMENTS

This work was funded by a Marie Curie IEF (grant no. 302113) and a Wellcome Trust ISSF to A.P., a BBSRC-DTP PhD studentship to R.S.G., and Natural Environment Research Council grant to P.A. (NE/M01939X/1). We wish to thank Prof. Dave Fernig for the FGF2, Prof. Brad Olwin for the anti-SDC3 antibody, and Ms. Lisa Olohan and Ms. Pia Koldkjaer, University of Liverpool, for their help with the microarrays. We also wish to thank the staff of the Biomedical Services Unit at the University of Liverpool for the superb care they take of the mouse colonies.

Received: October 11, 2016
Revised: January 11, 2018
Accepted: January 12, 2018
Published: February 8, 2018

REFERENCES

Arecco, N., Clarke, C.J., Jones, F.K., Simpson, D.M., Mason, D., Beynon, R.J., and Pisconti, A. (2016). Elastase levels and activity are increased in dystrophic muscle and impair myoblast cell survival, proliferation and differentiation. Sci. Rep. *6*, 24708.

Barouki, R., Coumoul, X., and Fernandez-Salguero, P.M. (2007). The aryl hydrocarbon receptor, more than a xenobiotic-interacting protein. FEBS Lett. *581*, 3608–3615.

Bespalov, M.M., Sidorova, Y.A., Tumova, S., Ahonen-Bishopp, A., Magalhães, A.C., Kulesskiy, E., Paveliev, M., Rivera, C., Rauvala, H., and Saarma, M. (2011). Heparan sulfate proteoglycan syndecan-3 is a novel receptor for GDNF, neurturin, and artemin. J. Cell Biol. *192*, 153–169.

Bjornson, C.R.R., Cheung, T.H., Liu, L., Tripathi, P.V., Steeper, K.M., and Rando, T.A. (2012). Notch signaling is necessary to maintain quiescence in adult muscle stem cells. Stem Cells *30*, 232–242.

Cerone, M.A., Marchetti, A., Bossi, G., Blandino, G., Sacchi, A., and Soddu, S. (2000). p53 is involved in the differentiation but not in the differentiation-associated apoptosis of myoblasts. Cell Death Differ. *7*, 506–508.

Cornelison, D.D., Filla, M.S., Stanley, H.M., Rapraeger, A.C., and Olwin, B.B. (2001). Syndecan-3 and syndecan-4 specifically mark skeletal muscle satellite cells and are implicated in satellite cell maintenance and muscle regeneration. Dev. Biol. *239*, 79–94.

Dumont, N.A., Wang, Y.X., and Rudnicki, M.A. (2015). Intrinsic and extrinsic mechanisms regulating satellite cell function. Development *142*, 1572–1581.

Gangoiti, P., Bernacchioni, C., Donati, C., Cencetti, F., Ouro, A., Gómez-Muñoz, A., and Bruni, P. (2012). Ceramide 1-phosphate stimulates proliferation of C2C12 myoblasts. Biochimie *94*, 597–607.

Ghadiali, R.S., Guimond, S.E., Turnbull, J.E., and Pisconti, A. (2016). Dynamic changes in heparan sulfate during muscle differentiation and ageing regulate myoblast cell fate and FGF2 signalling. Matrix Biol. *59*, 54–68.

Gopinath, S.D., Webb, A.E., Brunet, A., and Rando, T.A. (2014). FOXO3 promotes quiescence in adult muscle stem cells during the process of self-renewal. Stem Cell Reports *2*, 414–426.

Halevy, O. (1993). p53 gene is up-regulated during skeletal muscle cell differentiation. Biochem. Biophys. Res. Commun. *192*, 714–719.

Hoene, M., Runge, H., Häring, H.U., Schleicher, E.D., and Weigert, C. (2013). Interleukin-6 promotes myogenic differentiation of mouse skeletal muscle cells: role of the STAT3 pathway. Am. J. Physiol. Cell Physiol. *304*, C128–C136.

Jones, N.C., Fedorov, Y.V., Rosenthal, R.S., and Olwin, B.B. (2001). ERK1/2 is required for myoblast proliferation but is dispensable for muscle gene expression and cell fusion. J. Cell Physiol. *186*, 104–115.

Kanisicak, O., Mendez, J.J., Yamamoto, S., Yamamoto, M., and Goldhamer, D.J. (2009). Progenitors of skeletal muscle satellite cells express the muscle determination gene, MyoD. Dev. Biol. *332*, 131–141.

Kuang, S., Kuroda, K., Le Grand, F., and Rudnicki, M.A. (2007). Asymmetric self-renewal and commitment of satellite stem cells in muscle. Cell *129*, 999–1010.

Lazaro, J.B., Kitzmann, M., Poul, M.A., Vandromme, M., Lamb, N.J., and Fernandez, A. (1997). Cyclin dependent kinase 5, cdk5, is a positive regulator of myogenesis in mouse C2 cells. J. Cell Sci. *110* (Pt 10), 1251–1260.

Levine, A.J. (1997). p53, the cellular gatekeeper for growth and division. Cell *88*, 323–331.

Mashinchian, O., Pisconti, A., Le Moal, E., and Bentzinger, F.C. (2017). The muscle stem cell niche in health and disease. Curr. Top. Dev. Biol. Published online November 24, 2017. https://doi.org/10.1016/bs.ctdb.2017.08.003.

Nicolas, N., Marazzi, G., Kelley, K., and Sassoon, D. (2005). Embryonic deregulation of muscle stress signaling pathways leads to altered postnatal stem cell behavior and a failure in postnatal muscle growth. Dev. Biol. *281*, 171–183.

Olguín, H.C., and Olwin, B.B. (2004). Pax-7 up-regulation inhibits myogenesis and cell cycle progression in satellite cells: a potential mechanism for self-renewal. Dev. Biol. *275*, 375–388.

Olguín, H.C., and Pisconti, A. (2012). Marking the tempo for myogenesis: Pax7 and the regulation of muscle stem cell fate decisions. J. Cell. Mol. Med. *16*, 1013–1025.

Ono, Y., Boldrin, L., Knopp, P., Morgan, J.E., and Zammit, P.S. (2010). Muscle satellite cells are a functionally heterogeneous population in both somite-derived and branchiomeric muscles. Dev. Biol. *337*, 29–41.

De Palma, C., Falcone, S., Pisoni, S., Cipolat, S., Panzeri, C., Pambianco, S., Pisconti, A., Allevi, R., Bassi, M.T., Cossu, G., et al. (2010). Nitric oxide inhibition of Drp1-mediated mitochondrial fission is critical for myogenic differentiation. Cell Death Differ. *17*, 1684–1696.

Pisconti, A., Cornelison, D.D.W., Olguín, H.C., Antwine, T.L., and Olwin, B.B. (2010). Syndecan-3 and Notch cooperate in regulating adult myogenesis. J. Cell Biol. *190*, 427–441.

Pisconti, A., Banks, G.B., Babaeijandaghi, F., Betta, N.D., Rossi, F.M.V., Chamberlain, J.S., and Olwin, B.B. (2016). Loss of niche-satellite cell interactions in syndecan-3 null mice alters muscle progenitor cell homeostasis improving muscle regeneration. Skelet. Muscle *6*, 34.

Porrello, A., Cerone, M.A., Coen, S., Gurtner, A., Fontemaggi, G., Cimino, L., Piaggio, G., Sacchi, A., and Soddu, S. (2000). p53 regulates myogenesis by triggering the differentiation activity of pRb. J. Cell Biol. *151*, 1295–1304.

Sarker, K.P., and Lee, K.Y. (2004). L6 myoblast differentiation is modulated by Cdk5 via the PI3K-AKT-p70S6K signaling pathway. Oncogene *23*, 6064–6070.

Schmidt, K., Glaser, G., Wernig, A., Wegner, M., and Rosorius, O. (2003). Sox8 is a specific marker for muscle satellite cells and inhibits myogenesis. J. Biol. Chem. *278*, 29769–29775.

Soddu, S., Blandino, G., Scardigli, R., Coen, S., Marchetti, A., Rizzo, M.G., Bossi, G., Cimino, L., Crescenzi, M., and Sacchi, A. (1996). Interference with p53 protein inhibits hematopoietic and muscle differentiation. J. Cell Biol. *134*, 193–204.

Troy, A., Cadwallader, A., Fedorov, Y., Tyner, K., Tanaka, K., and Olwin, B. (2012). Coordination of satellite cell activation and self-renewal by par complex-dependent asymmetric activation of p38alpha/beta MAPK. Cell Stem Cell *11*, 541–553.

Tusher, V.G., Tibshirani, R., and Chu, G. (2001). Significance analysis of microarrays applied to the ionizing radiation response. Proc. Natl. Acad. Sci. USA *98*, 5116–5121.

Vassilev, L.T. (2005). p53 Activation by small molecules: application in oncology. J. Med. Chem. *48*, 4491–4499.

Walsh, E.M., Niu, M., Bergholz, J., and Xiao, Z.-X.J. (2015). Nutlin-3 down-regulates retinoblastoma protein expression and inhibits muscle cell differentiation. Biochem. Biophys. Res. Commun. *461*, 293–299.

Wang, C., Liu, W., Liu, Z., Chen, L., Liu, X., and Kuang, S. (2015). Hypoxia inhibits myogenic differentiation through p53 protein-dependent induction of Bhlhe40 protein. J. Biol. Chem. *290*, 29707–29716.

Wen, Y., Bi, P., Liu, W., Asakura, A., Keller, C., and Kuang, S. (2012). Constitutive Notch activation upregulates Pax7 and promotes the self-renewal of skeletal muscle satellite cells. Mol. Cell Biol. *32*, 2300–2311.

Yaffe, D., and Saxel, O. (1977). Serial passaging and differentiation of myogenic cells isolated from dystrophic mouse muscle. Nature *270*, 725–727.

Yang, Z.J.P., Broz, D.K., Noderer, W.L., Ferreira, J.P., Overton, K.W., Spencer, S.L., Meyer, T., Tapscott, S.J., Attardi, L.D., and Wang, C.L. (2015). p53 suppresses muscle differentiation at the myogenin step in response to genotoxic stress. Cell Death Differ. *22*, 560–573.

Yin, J., Sheng, B., Qiu, Y., Yang, K., Xiao, W., and Yang, H. (2016). Role of AhR in positive regulation of cell proliferation and survival. Cell Prolif. https://doi.org/10.1111/cpr.12282.

Yoshida, N., Yoshida, S., Koishi, K., Masuda, K., and Nabeshima, Y. (1998). Cell heterogeneity upon myogenic differentiation: down-regulation of MyoD and Myf-5 generates "reserve cells". J. Cell Sci. *111* (Pt 6), 769–779.

Yue, F., Bi, P., Wang, C., Li, J., Liu, X., and Kuang, S. (2016). Conditional loss of Pten in myogenic progenitors leads to postnatal skeletal muscle hypertrophy but age-dependent exhaustion of satellite cells. Cell Rep. *17*, 2340–2353.

Stem Cell Reports, Volume *10*

Supplemental Information

The Satellite Cell Niche Regulates the Balance between Myoblast Differentiation and Self-Renewal via p53

Valentina Flamini, Rachel S. Ghadiali, Philipp Antczak, Amy Rothwell, Jeremy E. Turnbull, and Addolorata Pisconti

The satellite cell niche regulates the balance between myoblast differentiation and self-renewal via p53

Valentina Flamini, Rachel S. Ghadiali, Philipp Antczak, Amy Rothwell, Jeremy E. Turnbull and Addolorata Pisconti

Supplementary Information

SUPPLEMENTARY METHODS

Myofibre cultures

Mice were culled under a CO_2 raising gradient and the gastrocnemius muscles dissected, incubated with 400 U/mL collagenase type I (*Worthington*) for 90 min then individual myofibres were picked using a glass Pasteur pipette whose tip had been flame-polished and flushed with fresh primary myoblast growth medium (F12 + 0.4 mM $CaCl_2$ + 15% horse serum + 1% penicillin + 1% streptomycin + 2 mM GlutaMax) in a cell culture incubator (humidified 37 °C, atmospheric O_2 and 5% CO_2). After 3 subsequent passages in fresh primary myoblast growth medium to remove debris and contaminant fibroblasts, FGF2 was added at the final concentration of 2 nM in the last passage. 2nM FGF2 was added again at 24 h and then the medium was changed once at 48 hours supplementing again with 2nM FGF2. Although it is still debated whether SCs derived from different myofibre types are also different and retain these different properties once cultured *ex vivo*, we chose to use the gastrocnemius muscle for myofibre preparations, rather than the most commonly used extensor digitorum longus (EDL) muscle, because the gastrocnemius muscle contains all myofibre types and therefore a myofibre-associated myoblast preparation obtained from the gastrocnemius muscle is more representative of all possible “types” of SCs than if the preparation was obtained from the EDL muscle, which contains almost exclusively fast twitch myofibres. This was especially important because the dispersed myoblast preparations were obtained from whole limb muscles and therefore they too contained all “types” of SCs. The same batch of horse serum was used throughout the work presented in this manuscript.

Myofibre-associated myoblast RNA quality control

We ran preliminary experiments to test for the presence of RNA from myofibre debris in our myofibre-associated myoblast preparations. We re-suspended the final pellet obtained after myoblast stripping into primary myoblast growth medium, plated this cell suspension on gelatin-coated dishes and the day after we extracted RNA from both the adherent cells and the supernatant. If significant amounts of contaminant RNA from the myofibres had been present in the final myoblast pellet, we should have been able to extract it and detect it from the supernatant of the plated myoblasts. Instead we only detected RNA from the adherent cell population not from the supernatant, which, once analysed by Bioanalyzer, showed only non-RNA contaminants (Fig. S7D-E), indicating that the final pellet obtained from myoblast isolation from myofibres is highly pure and does not contain myofibre RNA.

Preparation of dispersed myoblasts

Mice were culled under a CO_2 raising gradient and all the hind-limb muscles dissected, finely minced with scissors and then incubated with 400 U/mL collagenase type I for 60 minutes at 37 °C. After centrifuging at low speed (30 xg) for 3 minutes to pellet larger tissue debris, the supernatant containing mono-nucleated cells was collected, diluted with two volumes of primary myoblast growth medium (F12 + 0.4 mM $CaCl_2$ + 15% horse serum + 1% penicillin + 1% streptomycin + 2 mM GlutaMax) and then centrifuged again at 500 xg to pellet the mono-nucleated cells. Fibroblasts were removed via two subsequent pre-plating steps (2h + 1h, each one on gelatin-coated dishes, at humidified 37 °C, atmospheric O_2 and 5% CO_2). After the second pre-plating the floating cells were plated on gelatin-coated dishes in primary myoblast growth medium, supplemented with 2 nM FGF2 and incubated for 48 or 72 hours, with one medium change at 48 hours. This protocol yielded a preparation of

great purity (over 95% MYF5+/PAX7+ cells). For the microarray experiments, at 48 and 72 h after plating, cells where washed with phosphate buffered saline (PBS) and lysed with RLT buffer (Qiagen) prior to RNA extraction. For the reserve cell assays, 48 h after plating cells were washed twice with F12C (F12 + 0.4 mM $CaCl_2$) and then switched to primary myoblast low serum medium (F12 + 0.4 mM $CaCl_2$ + 3% horse serum + 1% penicillin/streptomycin + 2 mM GlutaMax) for 3 days. After 3 days, cells were either fixed and immunostained or “washed” with F12C and incubated again in primary myoblast growth medium (containing 15% horse serum) for 2 days, prior to being fixed and immunostained. The same batch of horse serum was used throughout the work presented in this manuscript.

C2C12 myoblast cultures

C2C12 myoblasts (Yaffe and Saxel, 1977) were cultured in C2C12 growth medium (DMEM + 10% foetal bovine serum + 2 mM L-glutamine + 1% penicillin + 1% streptomycin) up to 90% confluence then washed twice with DMEM and switched to C2C12 low serum medium (DMEM + 3% horse serum + 2 mM L-glutamine + 1% penicillin + 1% streptomycin) to induce differentiation and reserve cell generation (Yoshida et al., 1998) in the presence/absence of either 20 μM Nutlin-3 (Sigma Aldrich) or an equal volume of vehicle (DMSO, Sigma Aldrich) for an additional 3 days. After 3 days, cells were either immediately fixed and immunostained or “washed” with DMEM and re-incubated in C2C12 growth medium (containing 10% foetal bovine serum) for an additional 2 days prior to being fixed and immunostained. The same batch of foetal bovine serum was used throughout the work presented in this manuscript.

Antibodies

For immunofluorescence, primary antibodies used were: mouse anti-Pax7 (DSHB) 1:100, mouse anti-p53 (Cell Signaling, Cat No. 2524) 1:100, rabbit anti-myogenin (SCBT, Cat. No. sc-576) 1:500, rabbit anti-SDC3 (kindly donated by Prof Brad Olwin, University of Colorado (Cornelison et al., 2001) 1:500, rabbit anti-KI67 (Abcam, Cat. No. ab15580) 1:400, mouse anti-myogenin (DSHB, clone F5D) 1:100, mouse anti-myosin heavy chain (DSHB, clone MF20) 1:100, rabbit anti-MYF5 (SCBT, Cat. No. sc-302) 1:500, rabbit anti-MYOD (SCBT, Cat. No. sc-760) 1:600. Secondary antibodies conjugated to Alexa488, Alexa555 or Alexa647 (Invitrogen) were used at 1:500. DAPI (Invitrogen) was used at 2 μM in PBS.

For western blotting, primary antibodies used were: mouse anti-p53 (Cell Signaling, Cat No. 2524) 1:1,000; mouse anti-GAPDH (Sigma Aldrich, Cat. No. G8795) 1:3,000; mouse anti-myogenin (DSHB, clone F5D) at 1:1,000 and mouse anti-sarcomeric myosin (DSHB, clone MF20) at 1:3,000. Secondary antibodies were from Santa Cruz Biotechnology and used at 1:10,000. Chemiluminescence was detected on a LAS 4,000 (GE) image doc system.

Microscopy

Microscope images were acquired on an EVOS FL imaging system (Life Technologies) with a 10X, a 20X or a 40X objective and consistent imaging parameters.

Microarrays

Fluorescently labelled amplified complementary RNA (cRNA) was generated using a Low Input Quick Amp Labelling kit, One-Colour (Agilent). The method utilises an oligo (dT) primer bearing a T7 promoter, and MMLV-RT, producing double stranded

cDNA from mRNA; the cDNA then serves as template for *in vitro* transcription with T7 RNA Polymerase, which linearly amplifies target material whilst simultaneously incorporating cyanine 3-labelled CTP. For each labelling reaction, 70 ng of total RNA was used as input, along with appropriately diluted Spike Mix from the One-Colour RNA Spike-In kit (Agilent). cRNA was purified using an RNeasy Mini Kit (Qiagen) and quantified on a NanoDrop ND-1000 Spectrophotometer version 3.3.0. For array hybridisation, 600 ng of each cyanine 3-labelled cRNA, was combined with 5 µl of 10X Gene Expression Blocking Agent and 1 µl of 25X Fragmentation Buffer (both from the Agilent Gene Expression Hybridisation kit) in a total volume of 25 µl. Target mixtures were then incubated at 60 °C for 30 min to fragment the RNA to approximately 150 nucleotides. Fragmentation was terminated by cooling on ice for 1 min followed by addition of 25 µl of 2X Hi-RPM Hybridisation Buffer (Agilent). Agilent SurePrint 8X 60K Mouse Gene Expression microarrays (Design ID 028005) were loaded and hybridised using Agilent hardware, namely: gasket slides, SureHyb chambers and hybridisation oven. Hybridisation was carried out at 65 °C with 10 rotations per minute for 17 h. After this time, microarrays were washed using an Agilent Gene Expression Wash Buffer Kit according to the manufacturer's instructions. Arrays were scanned immediately at a resolution of 3 µm using an Agilent DNA Microarray Scanner to generate 20 bit tiff images. Data was extracted and QC reports generated using Agilent Feature Extraction version 11.0.1.1. Array quality was assessed by visual inspection of each tiff and analysis of the associated QC report, which indicates the dynamic range of the experiment, hybridisation and background uniformity, as well as an evaluation of metrics associated with the RNA spike-ins added to the labelling reactions.

Transfection

C2C12 myoblast cultures that had reached 90% confluence or myofibre cultures were washed twice with PBS and switched to OptiMem before receiving a 1:3, volume:volume mixture of siRNA (diluted in OptiMem for a final 30 or 60 nM):Lipofectamine2000. After addition of the siRNA:Lipofectamine2000 mixture, plates where incubated for three hours in a cell culture incubator (humidified 37 °C, atmospheric O_2 and 5% CO_2), then the medium was replaced with low serum medium that contained either 20 μM Nutlin-3 or an equal volume of DMSO and incubated for another day (for western blot analysis of C2C12 cells) or three days (for immunofluorescence analysis of C2C12 cells) or 2 days (for immunofluorescence analysis of myofibre cultures). siRNAs were either control (scrambled) siRNA (Sigma Aldrich) or anti-p53 siRNA (Sigma Aldrich, SASI_Mm02_00310137).

SUPPLEMENTARY FIGURES

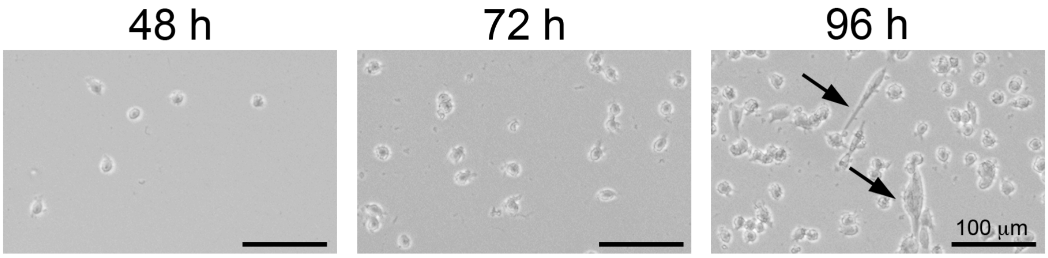

<u>Supplementary Figure S1 – supplementing Fig. 1A-B</u>: Dispersed myoblasts cultured on gelatin in the presence of high serum and FGF2 undergo spontaneous differentiation. Primary SC-derived myoblasts were cultured on gelatin-coated plates in primary myoblast growth medium supplemented with 2 nM FGF2 for 4 days. During the first 2-3 days cells mostly proliferated, around the third day they underwent spontaneous differentiation such that myotubes were visible at day 4 (black arrows).

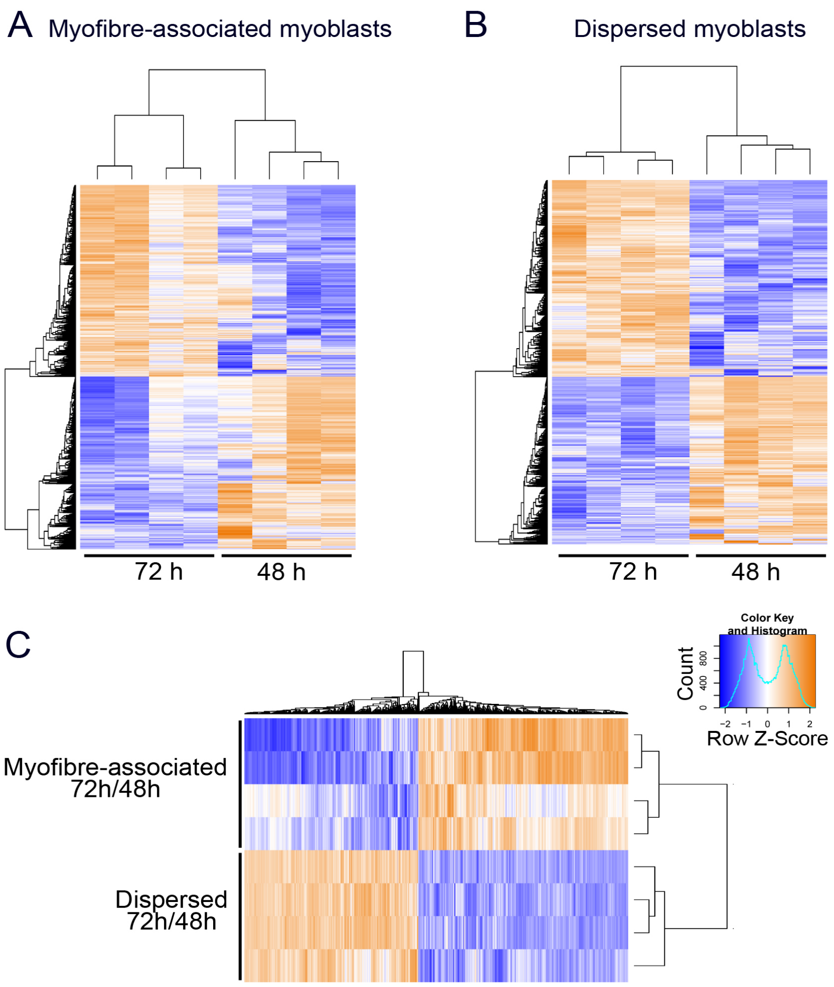

<u>Supplementary Figure S2 – supplementing Fig. 1E</u>: Replicate reproducibility was excellent in both dispersed and myofibre-associated myoblast cultures, which appeared very different in their transcriptome signature during myogenic progression. A-B) Hierarchical clustering of the Top 200 differentially expressed genes between 48 and 72 hours in culture in myofibre-associated (A) and dispersed (B) myoblasts. For each condition the biological replicates clustered together suggesting high reproducibility and high quality of the data. Moreover, as visually shown by the heatmaps, the transcriptomic signature at 48 hours is very different from the transcriptomic signature at 72 hours for both culture conditions. **C)** Gene expression regulation between 48 and 72 hours in myofibre-associated myoblasts and in dispersed myoblasts are different.

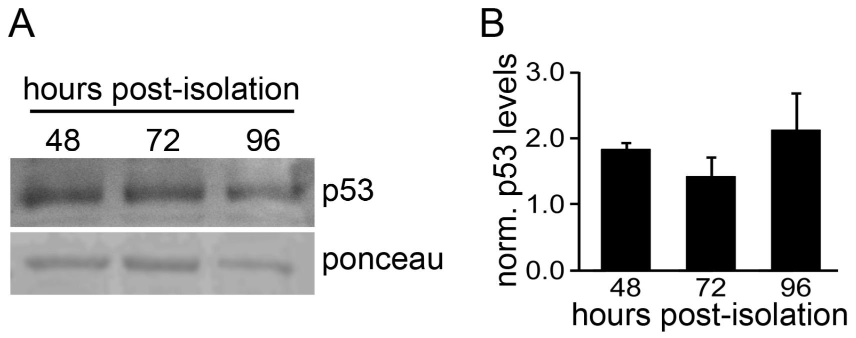

<u>Supplementary Figure S3 – supplementing Fig. 3</u>: p53 protein levels do not significantly increase over time in primary dispersed myoblast cultures. A-B) Primary dispersed myoblasts were cultured for up to 96 hours post-isolation, lysed at three time points: 48, 72 and 96 hours, then total proteins extracted and analysed by western blot to detect p53 levels. Ponceau staining was used as loading control. A representative western blot image is shown in (A) while (B) is the quantification of three independent experiments.

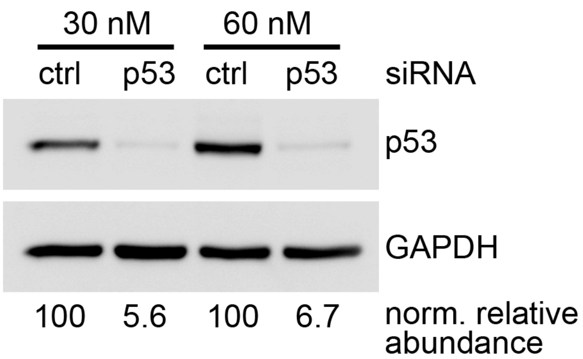

<u>Supplementary Figure S4 – supplementing Fig. 4</u>**: siRNA-mediated p53 knockdown is highly efficient.** C2C12 myoblast were cultured in growth medium until they reached 90% confluence then transfected with either control siRNA (30 nM, lane 1; 60 nM, lane 3) or anti-p53 siRNA (30 nM, lane 2; 60 nM, lane 4), 3 h later switched to low serum medium supplemented with either DMSO or 20 μM Nutlin-3 and cultured for an additional 24 h prior to being lysed and analysed by blot to reveal p53 (top gel) and GAPDH (bottom gel) abundance. The intensity of the p53 bands normalised to the intensity of the GAPDH bands are reported below the gel images as percentage of the control band for each one of the two siRNA concentrations tested. Since the level of knockdown was comparable between 30 nM and 60 nM siRNA, all subsequent experiments were carried out using 30 nM siRNA.

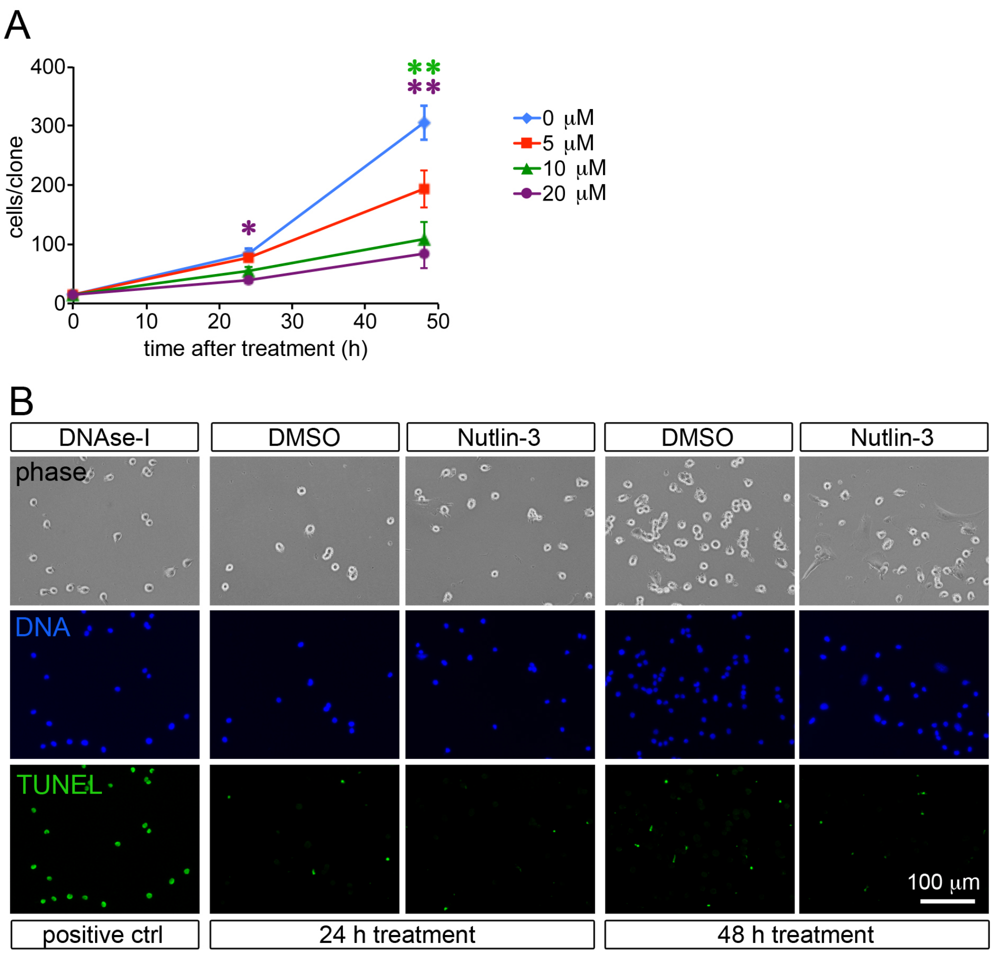

Supplementary Figure S5 – supplementing Fig. 5: Nutlin-3 treatment of primary dispersed myoblasts inhibits proliferation without increasing cell death. A) Primary dispersed myoblasts were expanded for two days prior to being detached and re-plated at clonal density for an additional three days. The day after plating (time 0 treatment) culture medium was supplemented with either DMSO or Nutlin-3 at 3 different concentrations: 5 μM, 10 μM or 20 μM. Samples were fixed either the day after (24 h treatment) or 2 days later (48 h treatment) and the numbers of cells/clone scored and plotted as average across 3 independent experiments ± S.E.M. At 24 h, only 20 μM Nutlin-3 significantly reduces proliferation (* = $p < 0.05$), at 48 h both 10 μM and 20 μM Nutlin-3 significantly reduce proliferation (** = $p < 0.01$). **B)** Primary dispersed myoblasts were plated and expanded for one day before being treated with either DMSO or 20 μM Nutlin-3, then fixed either the day after (24 h treatment) or 2

days later (48 h treatment), processed for TUNEL assay (green) and counter-stained with DAPI to detect DNA (blue). At each time point, a non-treated control was also fixed and treated with DNAse-I before TUNEL assay to induce DNA breakage – this is labelled as DNAse-I/positive control. Although in the positive control all cells are TUNEL+ indicating that the TUNEL assay worked, in both the DMSO and Nutlin-3-treated samples, at both time points, only very few TUNEL+ spots are detected, which do not co-localise with DAPI staining (hence the lack of quantification) and might therefore be an artifact or might be late apoptotic cells whose DNA was so extremely fragmented that no DAPI staining was detectable any more. In any case, there appear to be no difference between the DMSO-treated and the Nutlin-3-treated samples.

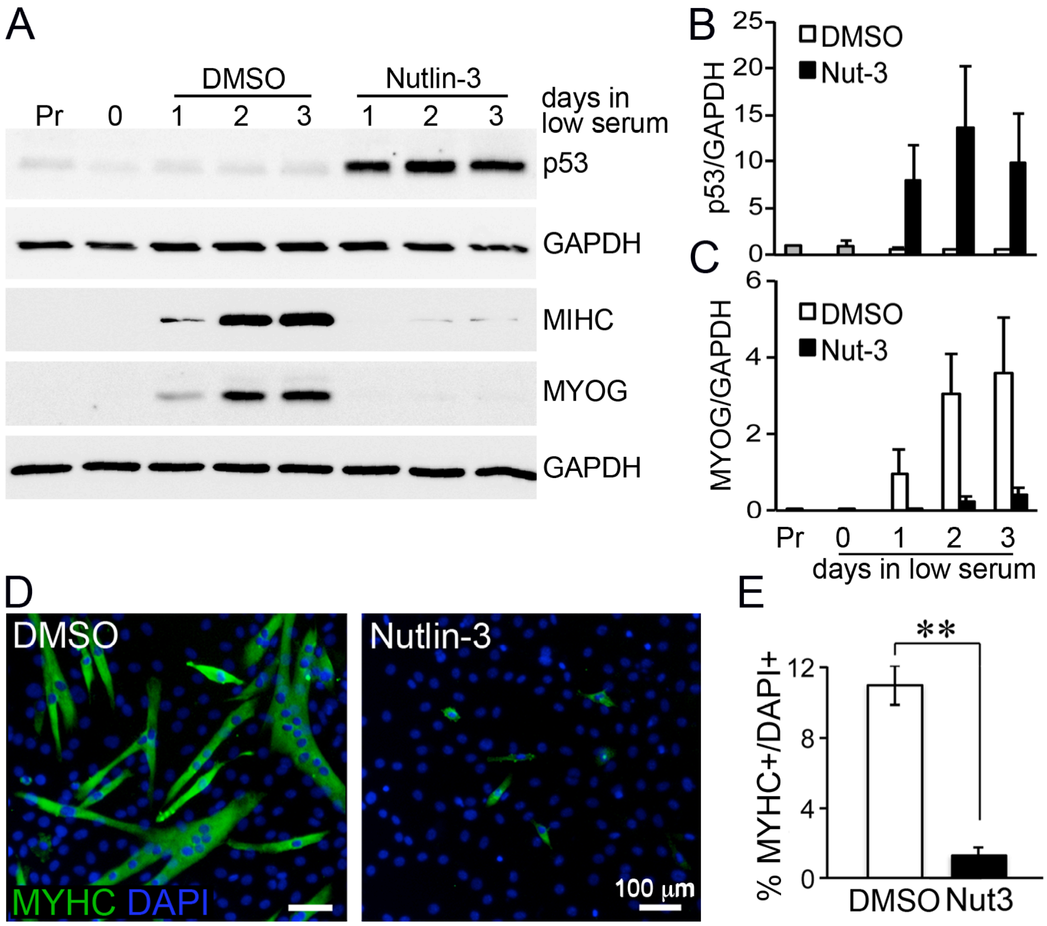

<u>Supplementary Figure S6 – supplementing Fig. 5 and Fig. 6</u>: **A sustained increase in p53 levels reduces C2C12 myoblast differentiation. A-C)** C2C12 myoblast cultures were grown to 90% confluence then switched to low serum medium (DMEM + 3% horse serum + 1% pen/strep + 2 mM L-glutamine) either in the presence of 20 μM Nutlin-3 or in the presence of an equal volume of DMSO and lysed at the following time points: Pr (= proliferating) is the day before reaching 90% confluence; 0 is the day when 90% confluence was reached; 1, 2 and 3 are the days subsequent to medium switch. At each time point cells were lysed and then all time points loaded onto a 10% SDS-PAGE for western blot analysis to reveal the abundance of myosin heavy chain (MYHC), myogenin (MYOG), p53 and GAPDH. Representative gels from three independent experiments are shown in (A) where to top GAPDH gel is related to the p53 gel, while the bottom GAPDH gel is related to

the MYHC and MYOG gel. In (B-C) is the quantification of three independent experiments carried out as in (A) where the average band intensity for p53 (B) or MYHC (C) normalised to the band intensity for its related GAPDH at each time point is plotted. Error bars are S.E.M. across the three independent experiments. **D-E)** Immunofluorescence analysis of C2C12 myoblasts switched to low serum medium when 90% confluence was reached and treated with either 20 μM Nutlin-3 or DMSO for 3 days prior to fixation and immunostaining to detect MYHC (green) and DNA (DAPI, blue) reveals that differentiation is reduced by Nutlin-3 treatment. In (D) one representative of several (> 10) independent experiments is shown. In (D) quantitation of three independent experiments across 10 technical replicates for each one of three biological replicates (N = 30) as the percentage of MYHC+ cells over the total numbers of DAPI+ cells is plotted. Error bars are S.E.M. ** = $p < 0.01$.

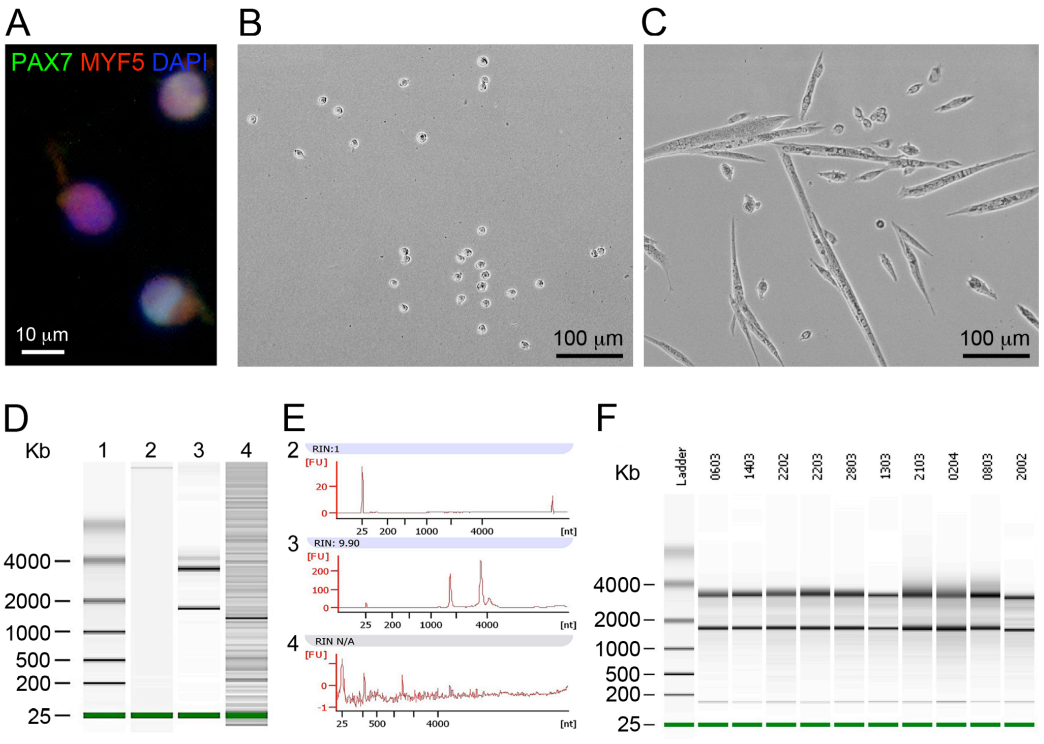

G

Sample name	Myoblas Prep	RNA extraction and purification method	Quantification method	Concentration (ng/ul)	RIN	NanoDrop 260/280 ratio
0603 (72h)	myofibre	Qiagen RNeasy kit	Bioanalyser	32.3	8.2	N.A.
1403 (72h)	myofibre	Qiagen RNeasy kit	Bioanalyser	15.7	8.9	N.A.
2202 (72h)	myofibre	Qiagen RNeasy kit	Bioanalyser	17.3	7.7	N.A.
1303 (72h)	myofibre	Qiagen RNeasy kit	Bioanalyser	8	7.5	N.A.
2203 (48h)	myofibre	Qiagen RNeasy kit	Bioanalyser	3.83	8.6	N.A.
2803 (48h)	myofibre	Qiagen RNeasy kit	Bioanalyser	7.7	8	N.A.
2103 (48h)	myofibre	Qiagen RNeasy kit	Bioanalyser	8.4	8.8	N.A.
0204 (48h)	myofibre	Qiagen RNeasy kit	Bioanalyser	11.6	7.4	N.A.
2002 (48h)	myofibre	Qiagen RNeasy kit	Bioanalyser	4.02	7.4	N.A.
1604 (48h)	myofibre	Qiagen RNeasy kit	Bioanalyser	3.2	8.7	N.A.
S1 (48h)	dispersed	Qiagen RNeasy kit	Nanodrop	12.2	N.A.	2.2
S2 (72h)	dispersed	Qiagen RNeasy kit	Nanodrop	22.0	N.A.	2.2
S3 (48h)	dispersed	Qiagen RNeasy kit	Nanodrop	25.0	N.A.	2.1
S4 (72h)	dispersed	Qiagen RNeasy kit	Nanodrop	32.9	N.A.	2.2
S5 (48h)	dispersed	Qiagen RNeasy kit	Nanodrop	35.6	N.A.	2.1
S6 (72h)	dispersed	Qiagen RNeasy kit	Nanodrop	39.2	N.A.	2.1
S7 (48h)	dispersed	Qiagen RNeasy kit	Nanodrop	47.2	N.A.	2.2
S8 (72h)	dispersed	Qiagen RNeasy kit	Nanodrop	66.6	N.A.	2.1

Supplementary Figure S7 – cited in *Experimental Procedures*: Validation of the protocol to isolate myofibre-associated myoblasts. A) Myofibre-associated myoblasts were stripped off myofibres using trypsin as described in the *Experimental Procedures* section then cytospun and immunostained to detect the SC/myoblast markers PAX7 and MYF5: 100% of the cells cytspun were positive for at least one of these two markers. **B-C)** Isolated myofibre-associated myoblasts were tested for viability and functionality by plating them on gelatin-coated dishes in primary

myoblast growth medium. Three days later isolated myofibre-stripped myoblasts had extensively proliferated in culture (B) and after switching them to low serum medium they underwent differentiation into myotubes (C). **D-E)** Bioanalyzer analysis of the RNA extracted from: negative control (water, lane 2); adherent cells (lane 3); supernatant of isolated myofibre-associated myoblasts plated on gelatin-coated dishes immediately after isolation (lane 4). Please not the massive differences in the Y-axis scales in the chromatograms shown in (E). This analysis reveals that no RNA was present in the supernatant indicating that no RNA was carried over from myofibre debris into the RNA preparations that were used for microarray analysis. **F)** Bioanlyzer analysis of the RNA extracted from all the biological replicates of myofibre-associated myoblasts at 48 hours and 72 hours post-myofibre isolation. Only for one of the 4 replicates of myofibre-associated myoblasts at 48 h, two distinct preparations were pooled to produce one biological replicate. In all other cases each biological replicate corresponded to one independent preparation. **G)** Table reporting, for each RNA sample used in the microarray experiment: sample name, type of myoblast prep, system for preparation, method of quantification, concentration, RIN where applicable and 260/280 ratio where applicable. All RNA samples were eluted in the same volume (14 µL) therefore the concentrations reported are a direct measure of the total amount of RNA extracted.

REFERENCES

Cornelison, D.D., Filla, M.S., Stanley, H.M., Rapraeger, A.C., Olwin, B.B., 2001. Syndecan-3 and syndecan-4 specifically mark skeletal muscle satellite cells and are implicated in satellite cell maintenance and muscle regeneration. Dev Biol 239, 79–94. doi:10.1006/dbio.2001.0416

Yaffe, D., Saxel, O., 1977. Serial passaging and differentiation of myogenic cells isolated from dystrophic mouse muscle. Nature 270, 725–727.

Yoshida, N., Yoshida, S., Koishi, K., Masuda, K., Nabeshima, Y., 1998. Cell heterogeneity upon myogenic differentiation: down-regulation of MyoD and

Myf-5 generates “reserve cells.” J Cell Sci 111 (Pt 6), 769–779.

Supplementary Table 1 – Cited in Results, section 4: Top three GO terms that are most significantly enriched in genes with CR > 2.

Term	p-value	Genes	Fold enrichment
Negative regulation of transport	0.00002	*Lif, Il6, Nos1, Snph, Edn1, Pkia, Adora1, Htr2a*	8.99
Striated muscle development	0.00004	*Musk, Actc1, Myl2, Tnc, Myog, Ttn, Neurl2*	10.48
Taxis	0.00005	*Ccr8, Ccl2, Cxcl3, Cxcl2, Ecscr, Amot, Ccl5, Ccl7, Slit2*	6.68

Supplementary Table 2 – Cited in Results, section 4: Top three GO terms that are most significantly enriched in genes with CR < 0.5.

Term	p-value	Genes	Fold enrichment
Cell adhesion	0.0004	*Col18a1, Selp, Pcdhb7, Pcdhb6, Pcdhb3, Pcdhb4, Itgb4, Cdhr5, Vtn, Pcdh17, Megf10, Dchs1, Cldn15, Aplp1, Wnt7b, Lamb3, Itga6, Lama5, Tro, Otog, Msln*	2.42
Cell morphogenesis involved in differentiation	0.0016	*Col18a1, Ablim1, Sema6a, Notch1, Slc1a3, Rtn4rl1, Cxcr4, Sema3a, Gas1, Ngfr, Cxcl12*	3.35
Cell fate commitment	0.0019	*Notch3, Fgfr4, Notch1, Tbx2, Pax7, Pparg, Cyp26b1, Gas1, Sox8*	3.96

Supplementary Table 3 – *Cited in Experimental Procedures and in Results, section 4*: Comparative analysis of genes that change between 48 and 72 hours according to the formula: $[(72h/48h)_{dispersed}]/[(72h/48h)_{myofibre\text{-}associated}]$ filtered through a manually curated list of genes involved in myogenesis.

Gene Name	Fold Change	q-value
Diras2	0.054013395	0.0000000
Mc4r	0.093310186	0.0000000
Aqp5	0.096597149	0.0000000
Ngfr	0.105205253	0.0000000
Cd5l	0.179134772	0.0000000
Sema6b	0.228863483	0.0000000
Trpc3	0.233711819	0.0235839
Fzd6	0.235680061	0.0000000
Fgfr4	0.236169718	0.0000000
Epha1	0.250161594	0.0000000
Itgb4	0.25121833	0.0000000
Pitx3	0.255210552	0.0000000
Itga6	0.263171022	0.0000000
Notch1	0.277726352	0.0000000
Col18a1	0.278990429	0.0000000
Tmem119	0.290233444	0.0000000
Notch3	0.291949448	0.0000000
Col10a1	0.300396372	0.0000000
Cxcr4	0.315090189	0.0000000
Cdkn2a	0.316454481	0.0000000
Ly6a	0.316960439	0.0000000
Sox8	0.323270186	0.0000000
Tmem44	0.328784523	0.0000000
Megf10	0.335582833	0.0000000
Cdc42ep1	0.337906165	0.0000000
Hes6	0.338134183	0.0000000
Pax7	0.344352698	0.0000000
Hey1	0.345761151	0.0000000
Cxcl12	0.348607325	0.0000000
Sema6a	0.349215321	0.0000000
Rnf152	0.350092944	0.0000000
Tmem191c	0.350341532	0.0235839
Pparg	0.361863203	0.0000000
Rnf144a	0.400662004	0.0000000
Cdkn2b	0.401434376	0.0000000
Lama5	0.412932128	0.0000000
Sema3a	0.41859219	0.0000000
Tcf7	0.423415284	0.0000000
Pdgfc	0.427927325	0.0235839
Tmem176a	0.428260942	0.0235839
Jam2	0.43211217	0.0000000
Pdgfrl	0.436975917	0.0000000
Sdc3	0.437947492	0.0000000

Timp4	0.442403723	0.0000000
Cnnm2	0.442786114	0.0000000
Mmp15	0.449408686	0.0000000
Rnf125	0.454254155	0.0000000
Lamb3	0.455321565	0.0000000
Rnf208	0.463874638	0.0000000
Tnfaip8l1	0.465211468	0.0000000
Tmem30b	0.471209484	0.0235839
Cd200	0.472625308	0.0000000
Fzd2	0.481798531	0.0000000
Ace	0.485118212	0.0000000
Lrp12	0.487980149	0.0000000
Wnt7b	0.491717209	0.0000000
Gper	0.493160426	0.0000000
Creb3l1	0.50032382	0.0000000
Tcf4	0.502701894	0.0000000
Tnfrsf25	0.511265133	0.0235839
Trim62	0.511388518	0.0000000
Megf9	0.512288348	0.0235839
Tmem176b	0.512579672	0.0335022
Chrna1	0.512895398	0.0000000
Ephb4	0.513271937	0.0000000
Cdh15	0.513597528	0.0000000
Igfbp4	0.518688547	0.0000000
Megf6	0.520352228	0.0335022
Tmem195	0.524521151	0.0000000
Cd276	0.526542761	0.0000000
Traf5	0.534764828	0.0235839
Map3k11	0.541734759	0.0000000
Cd2ap	0.550591348	0.0000000
Tmem121	0.56017007	0.0235839
Smad6	0.56064096	0.0000000
Tmem9	0.562422561	0.0000000
Il20rb	0.563193529	0.0000000
Tmem229b	0.571979361	0.0000000
Wnt6	0.579395389	0.0000000
Ctnnb1	0.58218307	0.0235839
Pth1r	0.583389597	0.0000000
Hspa12b	0.58409558	0.0000000
Il16	0.587001345	0.0235839
Cdc42ep4	0.591780882	0.0000000
Cd97	0.595509301	0.0000000
Jak3	0.600945954	0.0000000
Cd38	0.603198555	0.0235839
Tmem86a	0.606710437	0.0000000

Il18rap	0.608983054	0.0335022
Mmp11	0.61109568	0.0000000
F2r	0.618226226	0.0000000
Cdk19	0.618693237	0.0000000
Il34	0.619225504	0.0000000
Mmp17	0.62185378	0.0235839
Map4k2	0.623623769	0.0000000
Cxcr7	0.624860007	0.0000000
Hmgb3	0.624875968	0.0000000
Tmem173	0.630723339	0.0000000
Six2	0.631694902	0.0335022
Ctnnbip1	0.63742738	0.0335022
Fzd3	0.639107577	0.0335022
Socs2	0.64750778	0.0335022
Hmgn5	0.648216068	0.0235839
Stat2	0.658367534	0.0235839
Cdk2ap2	0.659850247	0.0000000
Met	0.665855282	0.0000000
Sdc4	0.667108623	0.0235839
Tcf3	0.668449518	0.0000000
Trim12	0.671382361	0.0335022
Ilkap	0.675550964	0.0335022
Ccna2	0.675932308	0.0235839
Mapk3	0.686093157	0.0000000
Itga3	0.688256775	0.0000000
Ilf2	0.689515315	0.0000000
Grb10	0.691704377	0.0000000
Tmem110	0.694182038	0.0335022
Itm2c	0.701841327	0.0335022
Cd3eap	0.704417076	0.0335022
Rnf38	0.704939689	0.0335022
Nfkbil2	0.705436177	0.0335022
Map3k12	0.711453353	0.0235839
Mybl2	0.712538267	0.0235839
Cdh8	0.733437491	0.0235839
Cdkn2c	0.733704766	0.0235839
Cd151	0.734003656	0.0235839
Casp2	0.740118071	0.0335022
Cdk10	0.742337344	0.0235839
Atoh8	0.743759899	0.0235839
Cdk2ap1	0.759884675	0.0335022
Tmem179b	0.790983217	0.0335022
Tmem50a	0.791326965	0.0335022
Col9a2	0.805013649	0.0235839
Nos2	0.808038002	0.0235839

Rnf160	1.182986633	0.0335022
Ppara	1.226111384	0.0235839
Rnf103	1.265119029	0.0000000
Abl2	1.28007608	0.0235839
Rnf181	1.28600617	0.0335022
Sod2	1.288438354	0.0235839
Tmem126a	1.297795278	0.0335022
Large	1.304309246	0.0335022
Tmem9b	1.31584281	0.0335022
Rnf170	1.32832226	0.0235839
Lamc1	1.338170117	0.0335022
Crybg3	1.352691383	0.0235839
Rnf19b	1.377284374	0.0335022
Nfix	1.428716223	0.0335022
Cd300lg	1.487985697	0.0335022
Itga2b	1.518339592	0.0335022
Cdc42ep3	1.520005509	0.0335022
Trim23	1.53151972	0.0335022
Cdkn1c	1.568324588	0.0235839
Ccng1	1.608196047	0.0335022
Tmem143	1.681525317	0.0000000
Ar	1.706092125	0.0235839
Mef2d	1.71779398	0.0000000
Tmem51	1.720317928	0.0335022
Myc	1.727137888	0.0235839
Tmem14a	1.741341997	0.0000000
Rnf113a2	1.748848333	0.0000000
Traf3	1.749643353	0.0000000
Rnf128	1.758049346	0.0000000
Col4a5	1.764058974	0.0235839
Hes2	1.807606777	0.0335022
Rnf123	1.822805482	0.0335022
Itga5	1.824026319	0.0335022
Tmem65	1.844739221	0.0000000
Mapk14	1.877026513	0.0000000
Tmem25	1.877475243	0.0000000
Tmem170	1.890353302	0.0335022
Myh7	1.904240751	0.0335022
Hdac9	2.039262001	0.0335022
Cd93	2.077909983	0.0335022
Ang2	2.119929417	0.0000000
Fgf10	2.136513435	0.0335022
Rnf39	2.142887692	0.0235839
Rnf115	2.185271495	0.0335022
Tmem74	2.189673882	0.0235839

Wnt5a	2.225643565	0.0000000
Tgfbr3	2.243237532	0.0335022
Nos1	2.244337119	0.0000000
Tnfaip3	2.370856078	0.0335022
Tmem22	2.382163203	0.0335022
Timp3	2.389738255	0.0000000
Grb14	2.450073063	0.0335022
Icam1	2.480875001	0.0000000
Cd34	2.502741332	0.0235839
Fgf13	2.522457048	0.0335022
Tnfrsf26	2.526705886	0.0335022
Cxcl1	2.571387986	0.0335022
Rnf150	2.613735241	0.0000000
Cxcr6	2.666414714	0.0235839
Hspb1	2.671942324	0.0335022
Igf2bp1	2.685073278	0.0335022
Lama4	2.694176556	0.0000000
Col11a1	2.81007029	0.0000000
Il1rl1	2.883004106	0.0235839
Angpt2	2.889406621	0.0000000
Ctgf	2.917124574	0.0000000
Trim55	2.943127311	0.0235839
Sema3c	2.962051241	0.0000000
Itga11	3.097928862	0.0335022
Smad3	3.143103306	0.0335022
Itgb7	3.180213711	0.0000000
Des	3.276458094	0.0335022
Wnt9a	3.473096788	0.0000000
Tmem132e	3.637282601	0.0000000
Tnfaip6	3.705210584	0.0000000
Crym	3.715182105	0.0335022
Pdgfra	3.799874363	0.0000000
Tmem56	3.824028259	0.0000000
Stac3	4.096755699	0.0000000
Itga2	4.151801886	0.0000000
Myl2	4.478819312	0.0000000
F3	4.53209333	0.0000000
Itgb1bp2	5.052382919	0.0000000
Cxcl2	5.21740209	0.0000000
Il6	5.883417321	0.0000000
Cxcl5	7.072889179	0.0335022
Mmp10	8.091003904	0.0000000
Mmp3	9.798800323	0.0000000
Csf2	10.41366572	0.0000000
Postn	13.13746879	0.0000000

Fgf21	13.98192167	0.0000000
Cxcl3	15.22060208	0.0000000
Myl3	18.83977572	0.0000000
Myog	20.03261426	0.0000000
Mmp13	28.20644517	0.0000000
